# Shared genetic variants across substance use disorders implicate common neurobiological pathways, a genome‐wide mixed methods study

**DOI:** 10.1002/gps3.70017

**Published:** 2026-04-20

**Authors:** Børge Holen, Zillur Rahman, Alexey A. Shadrin, Romain Icick, Kevin S. O’Connell, Linn Rødevand, Nadine Parker, Markos Tesfaye, Piotr Jaholkowski, Oleksandr Frei, Anders M. Dale, Srdjan Djurovic, Ole A. Andreassen, Olav B. Smeland

**Affiliations:** ^1^ Centre for Precision Psychiatry Institute of Clinical Medicine University of Oslo Oslo Norway; ^2^ Division of Mental Health and Addiction Oslo University Hospital Oslo Norway; ^3^ INSERM UMR‐S1144 Université Paris Cité Paris France; ^4^ Department of Psychiatry Institute for Genomics in Health SUNY Downstate Health Sciences University Brooklyn New York USA; ^5^ Department of Radiology University of California, San Diego La Jolla California USA; ^6^ Multimodal Imaging Laboratory University of California, San Diego La Jolla California USA; ^7^ Department of Cognitive Science University of California, San Diego La Jolla California USA; ^8^ Department of Psychiatry University of California, San Diego La Jolla California USA; ^9^ Department of Neurosciences University of California, San Diego La Jolla CA USA; ^10^ Department of Medical Genetics Oslo University Hospital Oslo Norway

**Keywords:** alcohol use disorder, cannabis use disorder, common neurobiological pathways, genetic architecture, novel substance use disorder genetic loci, opioid use disorder, shared genetics, substance use disorder

## Abstract

**Background:**

Substance use disorders (SUDs) are highly heritable, but the extent of shared and distinct genetic architecture across different SUDs is unclear.

**Aims:**

To compare the genetic architectures of alcohol use disorder (AUD), cannabis use disorder (CUD) and opioid use disorder (OUD) and to identify shared and unique genetic loci.

**Methods:**

We analysed large‐scale genome‐wide association study (GWAS) summary statistics from individuals of European ancestry recruited in Europe and the USA. The mixture model MiXeR was used to estimate the unique genetic architecture characteristics of each SUD, including its polygenicity, single nucleotide polymorphism (SNP)‐heritability and discoverability, a measure of the distribution of genetic signal across all causal variants. Pairwise conditional/conjunctional false discovery rate (cond/conjFDR) analyses identified shared loci, followed by biological annotation of implicated genes.

**Results:**

AUD demonstrated the highest polygenicity, followed by CUD and OUD. SNP‐based heritability was 0.10 for AUD and OUD and 0.01 for CUD. Discoverability was highest for OUD, followed by AUD and CUD. Currently, genome‐wide significant SNPs explain 2.0% of AUD, 0.3% of CUD and 0.2% of OUD variance. Cond/conjFDR identified 39 novel loci for AUD, 10 for CUD and 1 for OUD. Of implicated genes, most were expressed in the brain, including several involved in gamma‐aminobutyric acid and dopaminergic neurotransmission, opioid neurophysiology, myelination, DNA recombination, apoptosis and ubiquitin‐dependent protein catabolism.

**Conclusions:**

SUDs have polygenic architectures with many shared loci and are similar with regards to some characteristics. However, the level of polygenicity differs across SUDs, with AUD being considerably more polygenic than OUD, whereas CUD is intermediate in terms of its polygenicity. The novel loci implicate genes primarily expressed in the brain, involving a variety of biological functions. The findings expand our view of the aetiology of these disorders, while supporting the hypothesis of a shared set of pleiotropic SUD genes.

## Abstract (highlights)

**What is already known on this topic**

- Although previous analyses of genome‐wide association studies have demonstrated that substance use disorders (SUDs) are positively genetically correlated, only a handful of overlapping genetic variants have been identified to date and little is known about differences in the genetic risk architectures of these disorders.

**What does this study add**

- This study provides new insights into the shared genetic architecture of the SUDs—alcohol use disorder, cannabis use disorder and opioid use disorder—and identifies novel shared genetic variants.

**How might this study affect research, practice or policy**

- These results support the hypothesis of a shared set of pleiotropic SUD genes, providing new insights into the aetiology underlying these disorders.

## INTRODUCTION

Substance use disorders (SUDs) severely impact the health of individuals and represent a considerable societal burden.^1^ The courses of illness are heterogeneous, but are often prolonged and relapsing.^2^, ^3^ Both environmental and genetic factors are involved in SUD development. Twin and family‐based heritability estimates range from moderate to high for alcohol use disorder (AUD) (0.50–0.64),^4^ cannabis use disorder (CUD) (0.40–0.80),^4^ and opioid use disorder (OUD) (∼0.50).^4^

A key feature of SUDs is the overlapping clinical characteristics and high level of comorbidity between each disorder.^1^ SUDs likely share aetiology,^5^, ^6^, ^7^ and a general neuropathology of SUDs can be conceived as a homoeostatic adaptation in the cortico‐striatal and cortico‐limbic systems to exogenous substance intake.^5^ However, the pathobiological mechanisms underlying SUDs remain poorly understood. Genome‐wide association studies (GWASs) have discovered several risk loci for AUD,^8^, ^9^ CUD^10^ and OUD,^11^ some of which are shown to overlap.^12^ Furthermore, GWASs indicate a substantial shared genetic basis between SUDs, with genetic correlations (rg) ranging between 0.55 and 0.79 among AUD, CUD and OUD estimated using linkage disequilibrium (LD) score regression.^4^, ^6^, ^13^ However, only a minor fraction of the genetic architectures of SUDs have been identified to date. Although multivariate and post‐GWAS analyses^12^, ^14^, ^15^, ^16^, ^17^ have identified additional loci, the identification and characterisation of genetic variants for SUDs have been limited by relatively small sample sizes and a deeper understanding of the shared genetic architecture of these SUDs is lacking.

Intriguingly, several lines of evidence implicate both overlapping and distinct biological pathways underlying SUDs. Molecular findings suggest differences in the biology implicated across the different SUD loci. AUD risk loci have implicated genes distinctly involved in alcohol metabolism (e.g. *ADH1B*, *ALDH2*) and neurotransmission via the gamma‐aminobutyric acid (GABA) system.^18^ *CHRNA2*, a gene expressed in the brain, has been robustly associated with CUD risk.^10^, ^19^ The gene codes for the neuronal acetylcholine receptor (nAChR) α2 subunit and is thus involved in acetylcholine neurotransmission. Biological hypotheses include a role of Δ9‐tetrahydrocannabinol, the main psychoactive compound of cannabis, in direct or indirect action at this receptor, potentially affecting downstream dopamine release.^19^, ^20^ OUD loci identified by GWAS and post‐GWAS analyses have implicated the mu‐opioid receptor via the *OPRM1* gene^13^ and *FES*, a tyrosine kinase involved in post‐transcriptional and translational processes, both significantly expressed in the cortico‐striatal and cortico‐limbic circuits.^17^ Other studies provide evidence of overlapping neurobiological underpinnings. Multivariate GWAS have identified loci in or near *DRD2*, *PDE4B*, *FOXP2* and *BDNF* that are associated with multiple SUDs. Genes involved in the regulatory processes of dopamine are often implicated, highlighting broad biological roles in dopaminergic signalling and neurodevelopment.^21^ Further investigation of novel shared loci can advance efforts to elucidate the genetic and pathobiological basis of SUDs and the extensive comorbidity across them.^1^ Such progress can inform the development of risk prediction tools, thereby facilitating earlier and more targeted interventions.

Here, we aimed to further dissect the shared genetic architecture of major SUDs, focusing on AUD, CUD and OUD. We applied univariate mixed modelling (MiXeR)^22^ to quantify the number of common genetic variants influencing these disorders. Moreover, we employed a conditional/conjunctional false discovery rate (condFDR/conjFDR)^23^ approach to boost power to discover novel shared risk loci. Finally, we biologically characterised the identified variants and presented plausible underlying shared and non‐shared mechanisms of AUD, CUD and OUD.

## METHODS

Genetic correlation and multivariate GWAS primarily capture effect direction‐aligned pleiotropy and may therefore miss shared genetic architecture characterised by mixed effect directions. Indeed, the genetic relationship between many complex human phenotypes is shown to be characterised by a large number of overlapping genetic variants with a mixture of concordant and discordant effect directions.^24^, ^25^ As such, the complexity of the genetic relationships between human traits and disorders is not fully captured by genetic correlation estimates or multivariate GWAS. Furthermore, these tools do not capture polygenicity of the phenotype or determine the number of shared variants between two.^26^ The MiXeR framework^22^, ^26^ addresses this limitation by estimating the number of unique and shared genetic variants between two traits, whereas the cond/conjFDR approach^27^ leverages the shared genetic signal between two GWAS, irrespective of the effect directions, to increase statistical power for the discovery of individual genetic variants. Together, these methods provide complementary information on the shared genetic signal between two phenotypes at both global and local scales, beyond what is obtained through standard genetic correlation approaches or multivariate GWAS.

### Genome‐wide association studies samples

**FIGURE 1:**
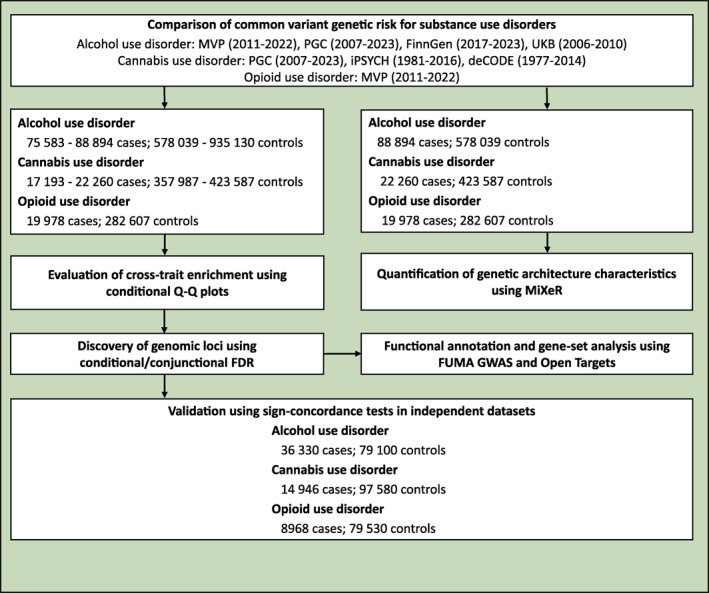
Flowchart of the study. FDR, false discovery rate; FUMA GWAS, Functional Mapping and Annotation of Genome‐Wide Association Studies; MiXeR, bivariate causal mixture model; MVP, Million Veteran Program; PGC, Psychiatric Genomics Consortium; Q‐Q, quantile‐quantile; UKB, UK Biobank.

We obtained publicly available independent GWAS summary statistics for International Classification of Diseases (ICD)^28^ and Diagnostic and Statistical Manual of Mental Disorders (DSM)^29^‐defined AUD, CUD and OUD. For MiXeR analysis of AUD, we utilised the largest possible datasets based on cohorts from the Million Veteran Program and FinnGen (*N*_case_ = 88 894, *N*_control_ = 578 039, publicly available).^30^ For MiXeR analysis of CUD, the largest available dataset was obtained from the Million Veteran Program (MVP) via a study by Levey et al.^31^ and largely the Psychiatric Genomics Consortium (PGC), iPSYCH and deCODE via a study by Johnson et al. (*N*_case_ = 39 453, *N*_control_ = 781 574, publicly available).^10^ For MiXeR and cond/conjFDR analyses of OUD, the largest available GWAS was obtained from the MVP (*N*_case_ = 19 978, *N*_control_ = 282 607, available on request).^32^ All GWAS data in the discovery analyses were from individuals of European ancestry. The OUD sample comprised opioid‐exposed controls only.^32^ To avoid sample overlap, in the cond/conjFDR analyses (described below), we included for AUD, datasets based on cohorts from the MVP, PGC, FinnGen and UK Biobank (*N*_case_ = 75 583, *N*_control_ = 935 130, available upon researcher request)^33^; we excluded PGC data in this AUD dataset in the analysis with CUD (*N*_case_ = 17 193, *N*_control_ = 357 987^10^ and excluded MVP data in the AUD dataset in the analysis with OUD). We ran sign concordance testing (binomial test) in African samples for validation purposes (AUD *N*_case_ = 36 330, *N*_control_ = 79 100; CUD *N*_case_ = 14 946, *N*_control_ = 97 580; OUD *N*_case_ = 8968, *N*_control_ *n* = 79 530). An overview of the samples is shown in table 1, further sample descriptions are presented in Supporting Information S1 and S2 and a flowchart of the study is shown in figure 1. The STREGA reporting guidelines were used in this study.^34^

**TABLE 1:** GWAS data for AUD (included severe acute intoxication, dependence and abuse), CUD (included dependence and abuse) and OUD^a^

Consortia	Phenotype (diagnostics)	*N*_case_	*N*_control_	Ancestry	Analysis	PMID
MVP	AUD (+ severe acute intoxication) (ICD‐9/10)	45 943	221 137	EUR	condFDR/conjFDR	40322774
PGC without FinnGen	Alcohol dependence (DSM‐IV)	11 042	44 030	EUR		
FinnGen	AUD (ICD‐9/10)	10 688	260 405	EUR		
UKB	Alcohol abuse/dependence (ICD‐10)	7910	409 558	EUR		
MVP	AUD (ICD‐9/10)	80 028	368 113	EUR	condFDR/conjFDR and MiXeR	38062264
		36 330	79 100	AFR		
FinnGen	AUD (ICD‐8/9/10)	8866	209 926	EUR		
PGC	Cannabis abuse/dependence (DSM‐III‐R/IV)	8402	24 265	EUR	condFDR/conjFDR	33096046
iPSYCH	Cannabis abuse/dependence (ICD‐10)	2758	53 326	EUR		
deCODE	CUD/cannabis abuse/dependence (DSM‐III‐R/IV/5)	6033	280 396	EUR		
MVP	CUD (ICD‐9/10)	22 260	423 587	EUR	condFDR/conjFDR and MiXeR	37985822
		14 946	97 580	AFR		
MVP	OUD (ICD‐9/10)	19 978	282 607	EUR	condFDR/conjFDR and MiXeR	36171425
		8968	79 530	AFR		

Abbreviations: AFR, African; AUD, alcohol use disorder; condFDR/conjFDR, conditional/conjunctional False Discovery Rate; CUD, cannabis use disorder; DSM, Diagnostic and Statistical Manual of Mental Disorders; EUR, European; GWAS, genome‐wide association study; ICD, International Classification of Diseases; MiXeR, Bivariate causal mixture model; MVP, Million Veteran Program; OUD, opioid use disorder; PGC, Psychiatric Genomics Consortium; PMID, PubMed identifier; UKB, UK Biobank.

^a^ See Supporting Information S1 for further details.

### Polygenic architecture characteristics

To quantify the number of trait‐influencing common genetic variants, we used a probabilistic Gaussian mixture model (univariate MiXeR),^22^, ^35^ which assumes that a given data set can be modelled as a ‘mixture’ of predefined components (zero and non‐zero effect on a trait), each with its own Gaussian distribution and a threshold of 90% heritability to avoid extrapolating model parameters into variants with infinitesimally small effects. The model incorporates the effects of minor allele frequency, sample size, LD structure and genomic inflation due to cryptic relatedness (see ref. 22 for details). We also utilised the model's ability for power calculations^22^ and for estimating discoverability, that is, the ability to discover single nucleotide polymorphisms (SNPs) for a given trait, based on the trait's unique genetic architecture.^36^

Bivariate MiXeR^26^ extends the univariate MiXeR framework by modelling a pair of traits, assuming that common genetic variants can be categorised into four components: shared causal variants, causal variants unique to trait one, causal variants unique to trait two and noncausal variants. Using model parameters derived from univariate MiXeR for each trait, bivariate MiXeR estimates the polygenicity of the shared component, independent of effect direction and correlation of effect sizes. The genome‐wide genetic correlation (rg) and the proportion of shared variants with concordant effects are inferred from these model parameters (see ref. 26 for details).

To assess model fit, the Akaike information criterion (AIC) difference between the best‐fitting MiXeR estimates and a reference model is calculated. A positive AIC difference indicates that the best‐fitting MiXeR estimates are distinguishable from the reference model. In univariate MiXeR, the reference model assumes an infinitesimal model in which all variants are causal. In bivariate MiXeR, the best‐fitting model is compared to scenarios of minimum and maximum possible overlap.

### Single locus identification with conditional/conjunctional false discovery rate

We evaluated cross‐trait enrichment among AUD, CUD and OUD using quantile‐quantile (*Q*‐*Q*) plots. In the presence of cross‐trait enrichment, we subsequently applied condFDR analysis^23^ to improve the discovery of genetic variants, leveraging genetic overlap across the six pairwise conditional combinations of SUD GWAS: AUD|CUD, CUD|AUD, AUD|OUD, OUD|AUD, CUD|OUD and OUD|CUD. The condFDR value can be interpreted as the probability that a given SNP is not associated with the primary trait, given that the SNP is more strongly or as strongly associated with both phenotypes than observed in the original GWAS. Finally, we computed a conjFDR statistic, which is determined as the maximum of the two condFDR statistics and represents the probability that a given SNP is not associated with the primary or secondary trait, given that the SNP is more strongly or as strongly associated with both phenotypes than observed in the original GWAS. An FDR level of 0.01 per pair‐wise comparison was set for condFDR and 0.05 for conjFDR, in line with the previous literature.^27^, ^37^ We excluded SNPs around the extended major histocompatibility complex (MHC) region and chr 8p23.1 (genome build 19 locations chr6:25 119 106–33 854 733 and chr8:7 200 000–12 500 000, respectively) before fitting the FDR model, since their intricate regional LD may bias cond/conjFDR estimation.^38^ More information about the cond/conjFDR methods can be found in the original publications^23^, ^39^ and review.^27^

### Genomic locus definition

We defined independent genetic loci according to the Functional Mapping and Annotation (FUMA) protocol.^40^ Briefly, independent significant genetic variants were identified as variants with conjFDR < 0.05 and LD *r* ^2^ < 0.60 with each other. A subset of these independent significant variants with LD *r* ^2^ < 0.10 was then selected as lead variants. For each lead variant, all candidate variants were identified as those with LD *r* ^2^ ≥ 0.60 to the lead variant. For a given lead variant, the borders of the genetic locus were defined as min/max positional coordinates over all corresponding candidate variants. Loci were then merged if they were separated by less than 250 kb. LD information was calculated from the 1000 Genomes Project European‐ancestry reference panel.^41^ Directional effects of loci were exhibited as concordant or discordant for the traits after comparing z‐scores of the lead SNPs. Concordance in the conjFDR analysis means that a risk allele in a locus is associated with the same directional effect in both traits (increased or decreased risk). We investigated all discovered loci for overlap with previously identified loci in relevant studies (see table S9) and in the National Human Genome Research Institute (NHGRI)‐European Bioinformatics Institute (EBI) Catalogue (see tables S3–S8). Novelty was defined for loci not previously associated with a trait after searching for specific loci where minimum and maximum base pair information was reported. In studies where only the variant position was available ± 1 million base pairs were added as boundaries. In addition, we searched the NHGRI‐EBI Catalogue with a list of terms for presence in relevant traits (see table S10). In table 2, we list novelty status by locus for each trait.

**TABLE 2:** Shared substance use disorder loci

Analysis	Chr:Pos:A1:A2	rsID	ConjFDR	Credibly mapped gene	PMID: Novel for AUD	PMID: Novel for CUD/OUD
AUD | CUD	1:66407352:T:C	rs7528604	4.17 × 10^−3^	*LEPROT*	30643251	37156939
	1:91209986:G:T	rs1526480	6.57 × 10^−3^	*BARHL2*	30940813	37156939
	2:27386676:T:C	rs112932070	4.00 × 10^−2^	*KHK*	34472679	Novel
	2:178087165:A:G	rs2588882	2.75 × 10^−2^	*NFE2L2*	Novel	Novel
	3:49874246:C:G	rs59684465	2.35 × 10^−2^	*RBM6*	38645045	38645045
	3:85596432:G:A	rs1463205	1.55 × 10^−2^	*CADM2*	32451486	37156939
	5:60489247:T:C	rs159544	1.24 × 10^−3^	*ELOVL7*	34472679	37156939
	6:19028788:C:T	rs975303	3.49 × 10^−3^	*N/A*	34472679	Novel
	6:26433329:G:GA	rs1624440	3.66 × 10^−3^	*HMGN4*	38062264	37208114
	6:40593860:C:T	rs2473569	2.84 × 10^−2^	*LRFN2*	Novel	Novel
	7:73006388:C:T	rs13225660	2.37 × 10^−2^	*MLXIPL*	38062264	Novel
	7:114129137:C:A	rs10249234	1.27 × 10^−2^	*FOXP2*	38645045	33096046
	8:2127805:G:A	rs73169501	3.18 × 10^−3^	*MYOM2*	38062264	Novel
	8:27442127:G:A	rs73229090	8.18 × 10^−3^	*EPHX2*	Novel	33096046
	9:81436959:G:A	rs7867205	3.02 × 10^−2^	*N/A*	Novel	Novel
	10:118619529:C:T	rs1637570	2.58 × 10^−2^	*SHTN1*	Novel	30940813
	11:27675712:A:G	rs1519480	2.83 × 10^−2^	*LIN7C*	38062264	37208114
	11:57563991:G:A	rs10896644	4.24 × 10^−3^	*MED19*	34472679	37208114
	11:113477081:G:A	rs11214677	2.32 × 10^−2^	*TMPRSS5*	34472679	30940813
	13:27098237:A:C	rs9507729	4.93 × 10^−2^	*WASF3*	Novel	Novel
	15:83503457:A:G	rs9806482	3.38 × 10^−3^	*GOLGA6L10*	Novel	30940813
	18:53305735:A:G	rs12458015	2.83 × 10^−4^	*TCF4*	38062264	37208114
	19:49244219:A:AT	rs2307018	2.63 × 10^−2^	*IZUMO1*	38062264	37208114
AUD | OUD	2:144250487:T:C	rs13428598	1.04 × 10^−2^	*ARHGAP15*	37156939	37156939
	6:19076417:C:T	rs9350100	3.38 × 10^−2^	*RNF144B*	34472679	35879402
	6:154360797:G:A	rs1799971	6.02 × 10^−3^	*OPRM1*	38062264	32492095
	7:135082751:C:T	rs3812281	7.41 × 10^−3^	*STMP1*	40322774	37156939
	8:93036795:C:T	rs9297901	2.12 × 10^−2^	*RUNX1T1*	38062264	37252880
	10:110604592:C:T	rs2685484	3.36 × 10^−2^	*N/A*	30940813	36171425
	11:112861434:C:T	rs4466874	2.37 × 10^−2^	*NCAM1*	37156939	35879402
	11:113294976:T:C	rs11608185	4.71 × 10^−2^	*TTC12*	37156939	35879402
	16:24759963:C:T	rs200527	3.73 × 10^−2^	*SLC5A11*	38062264	38244365
	16:30107781:G:A	rs73530179	3.98 × 10^−2^	*INO80E*	34472679	Novel
	16:53831146:T:C	rs9922708	3.09 × 10^−2^	*FTO*	30940813	35879402

Abbreviations: A1, Allele 1; A2, Allele 2; AUD, alcohol use disorder; Chr, chromosome; ConjFDR, conjunctional false discovery rate; CUD, cannabis use disorder; OUD, opioid use disorder; PMID, PubMed identifier; Pos, position.

### Functional analysis

We applied the open‐source Open Targets Genetics Variant‐to‐Gene, version 8, to map lead SNPs to genes, which integrates a variety of functional datasets in a machine‐learning approach to identify the most likely causal gene.^42^ We input the lead SNP for each locus and selected the gene with the highest overall score. Next, we employed gene set, cell and tissue expression analysis of disorder‐specific and cross‐disorder genes with FUMA.^40^ We finally investigated each gene separately for cell and tissue type, with the Human Protein Atlas (proteinatlas.org).^43^

## RESULTS

### AUD, CUD and OUD polygenicity and discoverability

Univariate MiXeR analysis indicated that AUD has the highest polygenicity (*n* = 9363 SD = 539), followed by CUD (*n* = 6059 SD = 419) and OUD (*n* = 5066 SD = 989; table S1), reflecting the estimated number of variants that account for 90% of the SNP‐heritability of each SUD (we additionally list estimates of 80% and 100% polygenicity in table S1). SNP‐based heritability estimates were similar (AUD and OUD: 0.10; CUD: 0.01). The most discoverable trait (discoverability is the variance of effect sizes across the associated SNPs, a measure of the distribution of genetic signal across all implicated variants),^36^ was OUD ($\sigma_{\beta }^{2}$ = 3.18 × 10^−5^), followed by AUD ($\sigma_{\beta }^{2}$ = 1.65 × 10^−5^) and CUD ($\sigma_{\beta }^{2}$ = 3.26 × 10^−6^). The Q‐Q plots (figure S1) and positive AIC values indicated a good fit for all univariate models (table S1). We also ran bivariate MiXeR (table S2), which yielded negative AIC values, indicating insufficient model fit for evaluating the extent of shared polygenic fractions, likely due to insufficient GWAS sample sizes.

### Cross‐trait enrichment

Conditional *Q*‐*Q* plots (figure S2) showed clear cross‐trait enrichment of SNP associations as evident by clear separation of the strata between AUD, CUD and vice versa, as well as for AUD, OUD and vice versa. For CUD conditioned on OUD and vice versa, there were no clear indications of cross‐trait enrichment (figure S2, bottom left and right panels). This was likely due to the modest GWAS sample sizes for CUD and OUD.

### Identification of loci associated with AUD, CUD and OUD and shared between SUDs

**FIGURE 2:**
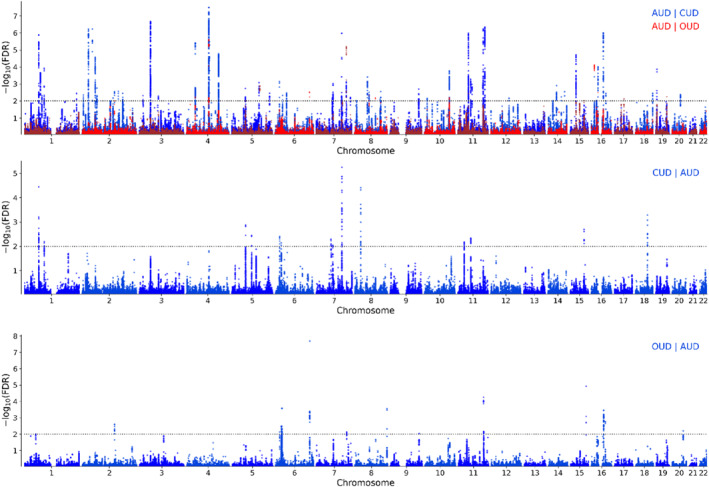
Conditional false discovery rate Manhattan plots. Upper panel: AUD conditioned on CUD (blue) and OUD (red). Middle panel: CUD conditioned on AUD. Bottom panel: OUD conditioned on AUD. AUD, alcohol use disorder; CUD, cannabis use disorder; FDR, false discovery rate; OUD, opioid use disorder.

**FIGURE 3:**
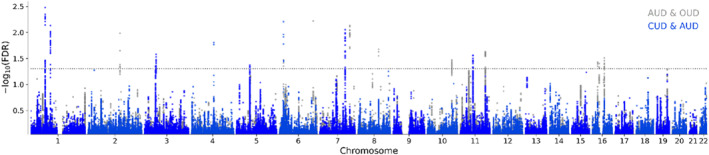
Conjunctional false discovery rate Manhattan plot. AUD with CUD and OUD. AUD, alcohol use disorder; CUD, cannabis use disorder; FDR, false discovery rate; OUD, opioid use disorder.

CondFDR analysis identified 115 loci associated with AUD when conditioned on CUD, 14 loci associated with AUD when conditioned on OUD, 17 loci associated with CUD when conditioned on AUD and 14 loci associated with OUD when conditioned on AUD at condFDR < 0.01 (tables S3–S6, figure 2). ConjFDR (conjFDR < 0.05) analysis revealed 23 shared loci between AUD and CUD and 11 between AUD and OUD (table 2, figure 3), whereas no shared loci were found between CUD and OUD due to limited cross‐trait enrichment (figure S2, bottom panels). Among the identified loci, 39 were novel for AUD, 10 were novel for CUD and 1 was novel for OUD (tables S3–S8). Notably, allelic effect directions were consistent across shared loci, at 96% (22/23) between AUD and CUD and 100% (11/11) between AUD and OUD, indicating shared genetic effects. Across European and African ancestries, lead SNP sign concordance varied between moderate for OUD (6/14, 43%, *p* = 0.172) and AUD (54/110, 49%, *p* < 0.001) to high for CUD (11/17, 65%, *p* = 0.038).

### Tissue and cell type specific enrichment analysis

Enrichment in differentially expressed gene sets in the GTEx v8 54 tissue types (figure S3) revealed that AUD genes (*n* = 108) were significantly upregulated in for example the basal ganglia, cerebellum, cerebral cortex and subcutaneous adipose tissue, whereas significantly downregulated in for example the stomach, heart left ventricle, breast tissue, non‐sun‐exposed skin, lung, spleen, uterus, aorta and testis and overall are significantly overexpressed in multiple brain regions. CUD implicated genes (*n* = 16) were significantly downregulated in lung tissue, whereas OUD implicated genes (*n* = 14) were significantly upregulated in the heart atrial appendage. In figure S4 (top panel), we show the cell type expression signature of the AUD genes, which were significantly associated with midbrain GABA‐ and dopaminergic neurons. A cross‐disorder analysis (loci with conjFDR < 0.05, *n* = 31) of gene overrepresentation showed an involvement of pancreatic delta cells (figure S4, bottom panel). The cell type expression signature analysis of CUD and OUD implicated genes was underpowered.

### Tissue expression of AUD, CUD and OUD implicated shared genes

We further investigated RNA expression tissue specificity of each implicated gene in the Human Brain Atlas^44^ (tables S3–S8). All genes in this study have previously been detected in the brain, except for *ADH1C*, which is enriched for the group ‘Intestine, Liver, Stomach’ identified in our AUD analysis conditioned on OUD. Implicated biological processes include GABA, opioid and dopamine neurophysiology (e.g. *BARHL2*, *OPRM1*, *FOXP2*, *TTC12*, *FTO*), myelination (*SLC5A11*), DNA recombination (*INO80E*), apoptosis and ubiquitin‐dependent protein catabolism (*RNF144B*). We further present tissue and cell type expression of cross‐SUD genes in figures S5 and S6. Of the 31 shared SUD genes, all were expressed in neuronal and glial cells, including the cortico‐striatal and cortico‐limbic circuits.

## DISCUSSION

### Main findings

In the current study, we show that the polygenic architectures of AUD, CUD and OUD were comparable, with similar SNP‐based heritability and discoverability estimates, whereas AUD exhibited a higher polygenicity. In total, we identified 39 novel AUD loci, 10 novel CUD loci and one novel OUD locus (tables S3–S8) and identified 34 overlapping SUD loci. The functional analyses implicated GABA, opioid and dopamine neurophysiology, myelination, DNA recombination, apoptosis and ubiquitin‐dependent protein catabolism. Together, our findings provide new insights into the genetic architectures and possible molecular biological pathways shared across SUDs.

Novel loci implicated genes with relevant SUD biology. Highlighted here is *NFE2L2* at chr 1 that codes for a transcription factor which plays a wide role in neuroprotection from oxidative and electrophilic stress induced by endogenous and exogenous sources^45^ and is novel for AUD and CUD. This finding may indicate a reduced cellular stress capacity in patients with AUD and CUD. Also novel for AUD and CUD is *LRFN2*, which codes for a neuronal membrane protein involved in synaptic transmission.^46^ It was associated with antisocial personality disorder in this trait's first GWAS^47^ and supports the increasing findings of genetic comorbidity between externalising traits.^48^ On chromosome (chr) 6, we identified a locus implicating *OPRM1*, a gene strongly associated with OUD, coding for the mu opioid receptor.^13^ It was recently associated with AUD,^30^ indicating shared molecular substrates for alcohol and OUDs (however, *OPRM1* has long been known to be involved in other alcohol‐related traits^49^). *INO80E*, a gene mapped to a novel locus for OUD, is detected in all tissues, especially widely in the brain and in both glial and neural cells. It is likely involved in DNA recombination.^50^ In our conjFDR analysis, a locus on chr6 implicating the gene *RNF144B* was significantly shared between AUD, CUD and OUD, suggesting a highly relevant SUD locus. *RNF144B* is a mitochondrial membrane transferase involved in apoptosis and ubiquitin‐dependent protein catabolism and is highly expressed in monocytes of the innate immune response and also in keratinocytes, glial and neural cells.^44^ Variants affecting *RNF144B* may thus potentially impact immune and neuronal function through horizontal pleiotropy (variants affect multiple traits independently). Mounting evidence suggests pleiotropy is common with complex traits, such as SUDs, underlining the probable complex downstream mechanistic effects.^14^, ^16^, ^17^, ^33^

We further corroborate previous studies indicating altered dopamine neurotransmission in reward relevant areas via *NCAM1*,^51^ *OPRM1*,^52^ *FOXP2*,^53^ *TTC12* ^54^, ^55^ and *FTO*.^56^ Across traits, there was an overrepresentation of expression of genes in the pancreas delta cells gene set and most implicated shared genes were expressed in neuronal and glial cells of the human brain, including the cortico‐striatal and cortico‐limbic circuits. There was also a high level of non‐specificity in gene expression; however, the brain remained the tissue with a relatively higher number of genes showing an increased expression pattern. In the shared loci of the conjFDR analyses, all loci but one exhibited allelic sign concordance between the traits in EUR ancestry, in line with the correlated genetic risk between the SUDs^4^, ^6^, ^13^; this suggests shared molecular genetic mechanisms and a common genetic basis of SUDs. The variance in the between‐ancestries sign concordance may be due to a genuine difference in biological substrates. There may also be asymmetries in environmental factors across ancestries, biasing health care seeking for issues related to some substances but not for others, influencing SUD phenotypes across ancestries. Current low statistical power in the African samples is likely also a contributing factor.

Interestingly, the implicated genes may differentially map to general addiction liability as opposed to substance‐specific risk.^57^, ^58^ On a cautious note, however, the presently identified genes only represent a fraction of the genetic architectures of the respective SUDs, given current GWAS power. We therefore cannot exclude that some of the genes that currently display SUD‐specific associations may become associated with several SUDs as GWAS power improves. Allostatic models describe addiction as a persistent shift in reward–stress regulation mediated by cortico‐striatal and limbic circuitry.^59^, ^60^ Supporting this model and a general addiction liability is the locus on chromosome 6 implicating *RNF144B*, shared between AUD, CUD and OUD, which acts as an immune signalling modulator, potentially by altering set points for inflammatory responses to drugs, infections, injury or other stressors,^61^ thereby affecting liability to develop or maintain phenotypes such as SUDs, pain or mood symptoms. AUD has been linked to altered oxidative stress responses and stress‐system recruitment.^62^, ^63^, ^64^ This may be consistent with the implication of genes *NFE2L2* and *INO80E*, which are associated with variation in cellular stress and transcriptional regulation.^65^, ^66^, ^67^ Cannabis‐related liability was associated with genes affecting cortical and synaptic regulation (*NCAM1*, *FOXP2*), consistent with altered endocannabinoid modulation of plasticity and stress buffering^68^, ^69^, ^70^; however, these genes are not substance‐specific. Likewise, the well‐established OUD risk locus implicating the *OPRM1* gene alters mu opioid receptor function and has been proposed to influence OUD risk by modulating opioid‐induced reward and relief of pain or negative affect.^71^ However, it is also associated with AUD,^30^ albeit to a weaker degree, strengthening the notion of shared addiction mechanisms underlying SUDs, plausibly through common biological pathways. The observation that the mu opioid receptor antagonists naltrexone and nalmefene are effective against AUD further strengthens this point, beyond shared liability.^72^

**FIGURE 4:**
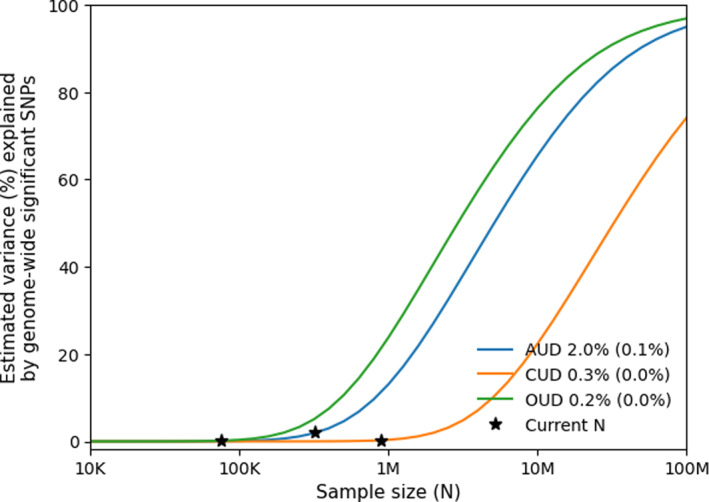
Power curves. Showing power calculations by univariate MiXeR. It displays current sample sizes and projections of the sample sizes required to explain a proportion of narrow‐sense heritability, as captured by genome‐wide significant SNPs for AUD, CUD and OUD. AUD, alcohol use disorder; CUD, cannabis use disorder; K, thousand; M, million; OUD, opioid use disorder; SNPs, single nucleotide polymorphisms.

Our study indicates widespread pleiotropy across SUDs. This is consistent with high genetic correlations across SUDs and the fact that polygenic risk scores (PRS) for one SUD often predict another SUD to a significant degree.^73^ For example, recent work demonstrates that a PRS derived from a general addiction factor (combining genetic effects across AUD, CUD, OUD, etc.) outperforms substance‐specific polygenic scores in predicting broad outcomes such as co‐occurring psychiatric illness and severe addiction phenotypes.^21^ Further, another configuration of traits (e.g. externalizing) similarly converges on overlapping risk with SUDs,^74^ indicating that the conglomeration of genetic risk into biologically meaningful buckets may be of nosological value. However, genetic correlations between SUDs and other constructs do not seem to make clear the full scope of such relationships, as we can see from examples where we have near zero genetic correlation between two traits, however still finding a large genetic overlap (e.g. schizophrenia and education attainment).^26^ Future studies may benefit from methods that go beyond genetic correlation to clarify these relationships. As indicated by the GWAS power plot generated using MiXeR^22^ (figure 4), most variants contributing to the genetic architecture of SUDs have not yet reached genome‐wide significance. This demonstrates the importance of continuing the assembly of large‐scale GWAS for SUDs to ensure further progress in this field. At current GWAS sample sizes for AUD, CUD and OUD, only 2.0% (SD = 0.1%), 0.3% (SD = 0.0%) and 0.2% (SD = 0.0%) of their SNP‐heritabilities, respectively, are estimated to be explained by genome‐wide significant SNPs. Accordingly, the discovery trajectory for SUD GWAS still trails that of other complex phenotypes, such as other mental disorders.^75^ Although we expect to see large increases in the elucidation of SUD genetic architectures as larger GWAS samples are acquired, the present study also demonstrates the value of leveraging advanced statistical methods to improve the yield of existing data in a cost‐efficient manner.^27^, ^76^

### Limitations

Although the lack of cross‐trait enrichment between CUD and OUD could potentially reflect a true lack of shared genetic underpinnings, this would be inconsistent with their known genetic correlation^6^ and clinical comorbidity.^77^, ^78^ Furthermore, taking our power calculations into account (figure 4), in which both GWASs display weak power at the present stage, it is likely that insufficient statistical power rather explains their lack of detectable cross‐trait enrichment. The fact that both the CUD and OUD datasets display cross‐trait enrichment with the larger AUD dataset further strengthens this hypothesis. As such, we expect that assembly of larger samples will address this issue, possibly after substantially increasing the number of cases included in each GWAS.

Identifying true causal variants is confounded by LD and SNP associations. Long‐range LD in the MHC region may bias findings and thus, this region was excluded before fitting the FDR model and prior to MiXeR analysis. Diagnostic harmonisation across all cohorts was assured by including only cases with at least one ICD or DSM trait code. Although MiXeR can handle cohort overlap, we excluded overlapping cohorts in the cond/conjFDR analyses to ensure no systematic bias in the results. However, we cannot exclude the possibility that misdiagnosis of cases may have affected the precision of the estimates, leading to a more heterogeneous genetic signal. Larger sample sizes with precise diagnostics are important for further elucidation of the unique and shared genetic architectures of SUDs. Improvements and synchronisation across consortia are important. Focusing on individuals of European ancestry is a limitation in the field and the need for diverse population studies is present.

### Implications

This work characterises SUDs' genetic architectures quantitatively and identifies specific implicated genetic loci. It harmonises with and expands recent years' work in psychiatric genetics,^24^, ^79^, ^80^ indicating that the genetic susceptibility of SUDs and other mental disorders is continuous with one another and thus dimensional in nature.^16^, ^80^ As such, these findings may be at odds with the categorical classification of disorders as provided by the main diagnostic frameworks, the ICD and DSM. These current descriptive diagnostic systems do not account for the large degree of shared genetic influences or clinical features between SUDs and may therefore propagate misconstrued views about the inherent boundaries between diagnostic categories. For example, in the present study, we have identified several shared variants that appear to confer risk to multiple SUDs, indicating that a given individual with a heightened risk for one SUD is likely to be at increased risk also for other SUDs. Although much work remains in order to operationalise this knowledge into the clinic in a systematic way and clarify the actual shared biology involved, these findings may ultimately inform novel approaches to develop classification frameworks that better align with the underlying pathogenesis, including transdiagnostic models such as The Research Domain Criteria and the Hierarchical Taxonomy of Psychopathology.^81^

## AUTHOR CONTRIBUTIONS

Børge Holen and Olav B. Smeland were responsible for the study concept and design. Alexey A. Shadrin, Kevin S. O’Connell, Ole A. Andreassen and Olav B. Smeland contributed to the acquisition of summary data. Zillur Rahman, Børge Holen, Olav B. Smeland and Ole A. Andreassen assisted with data analysis and interpretation of findings. Zillur Rahman and Børge Holen prepared figures. Børge Holen drafted the manuscript. Ole A. Andreassen and Olav B. Smeland provided critical revision of the manuscript for important intellectual content. All authors critically reviewed the content and approved the final version for publication.

## CONFLICT OF INTEREST STATEMENT

Ole A. Andreassen has received speaker honoraria from Sunovion, Lundbeck and Janssen and is a consultant for Cortechs.ai and Precision Health. Anders M. Dale is a founder of and holds equity interest in CorTechs Labs and serves on its scientific advisory board. He is also a member of the Scientific Advisory Board of Healthlytix and receives research funding from General Electric Healthcare (GEHC). The terms of these arrangements have been reviewed and approved by the University of California, San Diego in accordance with its conflict‐of‐interest policies. The remaining authors have no conflicts of interest to declare.

## ETHICS STATEMENT

The Regional Committee for Medical Research Ethics South East Norway has evaluated the current protocol and found that no additional institutional review board approval was needed, as individual data were not used.

## Supporting information

Supporting Information S1

Supporting Information S2

## Data Availability

Cond/conjFDR analyses were run with MATLAB 2018b and code is available at https://github.com/precimed/pleiofdr. MiXeR software version 2.2.1 was used and code is available at https://github.com/precimed/mixer. Version 1.8.1 of the FUMA web tool was used (https://fuma.ctglab.nl/updates). We used version 8 of the OpenTargets tool (https://genetics.opentargets.org/) and the clean_sumstats pipeline to standardize summary statistics (https://github.com/precimed/GWAS_SUMSTAT).
