# Supplementary material for: Shared genetic variants across substance use disorders implicate common neurobiological pathways, a genome‐wide mixed methods study
Source: Gen Psychiatr. 2026 Apr 20;39(2):e70017. doi: 10.1002/gps3.70017 (PMC13095382; doi:10.1002/gps3.70017)
Supplement: Supplementary file 1 — Supporting Information S1 [file GPS3-39-e70017-s001.docx]

## Shared genetic variants across substance use disorders implicate common neurobiological pathways, a genome-wide mixed methods study.

## SUPPLEMENTARY METHODS

### **Alcohol use disorder (AUD) datasets**

Million Veteran Program (MVP)

Pre-release 4. Participants included one inpatient or two outpatient ICD-9/10 codes for AUD and or severe acute intoxication. Diagnoses were based on validated electronic health records. We accessed AUD data from the dbGaP website with accession number phs001672.v9.p1. The summary statistics were not available without intoxication cases, which only represented 0.3% of the sample (N=226). Cases (45 943) and controls (221 137) were of European ancestry. See original publication for further information^1^

Release 4 summary statistics were available online from (<https://medicine.yale.edu/lab/gelernter/stats/>). All participants included had at least one inpatient or two outpatient ICD-9/10 codes for AUD. Controls had no ICD codes for AUD. Participants with one outpatient diagnosis were excluded. In total, 80 028 cases and 368 113 controls of European ancestry and 36 330 cases and 79 100 controls of African ancestry were included^2^.

FinnGen

Data freeze 5 summary statistics were available online from (<http://r5.finngen.fi/>) and 8866 cases and 209 926 controls of European ancestry defined by ICD-8/9/10 codes were included^3^.

*Cond/conjFDR analysis with OUD (No MVP)*

FinnGen

The R6 public release (<https://www.finngen.fi/en/access_results>)^3^ using ICD-9/10 abuse/dependence AUD diagnoses included 10 688 cases based on validated electronic health records from in- or outpatient care and 260 405 controls without AUD diagnoses of European ancestry.

UK Biobank (UKB)

AUD diagnosed through ICD-10 abuse/dependence criteria was based on validated electronic health records from inpatient and primary care settings in the United Kingdom (accession number 27412) and included 7910 cases and 409 558 controls of European ancestry.

Psychiatric Genomics Consortium (PGC) minus FinnGen

Cases (11 042) were diagnosed from trained clinicians’ ratings or semi-structured interviews as DSM-IV alcohol dependence (deemed equivalent to severe DSM-5 AUD) and were not publicly available without a FinnGen cohort and required a request to the PGC (<https://pgc.unc.edu/for-researchers/download-results/>). Number of controls were 44 030. Cases and controls were of European ancestry.

### **Cannabis use disorder (CUD) datasets**

MVP

Data release version 4, including ICD codes for cannabis dependence or cannabis abuse (together, CUD) was queried in EHRs. At least one Veterans Affairs (VA) US inpatient or outpatient visit for CUD was used as case definition. Controls were defined as participants with no VA EHR ICD codes for cannabis dependence, cannabis abuse or cannabis use (ICD-9 codes included: 305.29 and ICD-10 codes included: F12.90, F12.920, F12.921, F12.922, F12.929, F12.93, F12.950, F12.951, F12.959, F12.980, F12.988 and F12.99). This included 22 260 cases and 423 587 controls of EUR ancestry in the discovery analysis and 14 946 cases and 97 580 controls in the validation sample. Please see original publication for further details^4^.

Johnson et al 2020^5^

PGC cases were defined with clinician ratings or semi-structured interviews according to DSM-IV/III-R lifetime diagnosis of cannabis abuse or dependence. iPSYCH cases were defined as ICD-10 F12.1 (cannabis abuse) and F12.2 (cannabis dependence) and controls defined as without these diagnoses. deCODE cases were defined with DSM-III-R/IV/5 and included lifetime cannabis abuse, dependence or DSM-5 cannabis use disorder, with population controls. Participants were of EUR ancestry and comprised 17 193 cases and 357 987 controls. Cannabis exposure was not a criterion for controls overall, due to lack of data.

### **Opioid use disorder (OUD) dataset**

MVP

The definition of OUD required at least one inpatient or outpatient diagnostic code for OUD based on ICD-9/10 codes 304.0, 304.7, 305.5, F11.1, or F11.2 in EHRs. Controls were defined as having at least one outpatient opioid prescription filled minus an OUD diagnosis code documented in the EHRs. Please see ^6^ for further descriptions. The discovery analysis included cases (19 978) and controls (282 607) of European ancestry, while the validation sample included cases (8968) and controls (79 530) of African ancestry.

## SUPPLEMENTARY FIGURES

### Figure S1


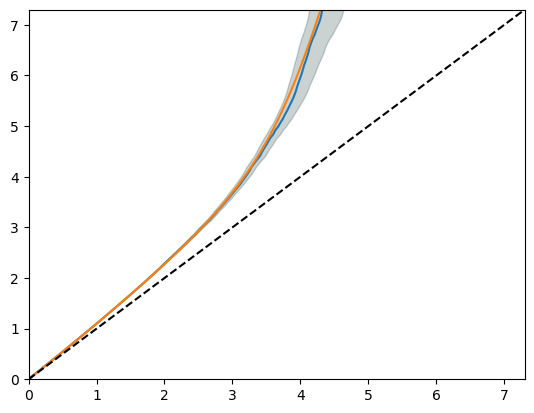

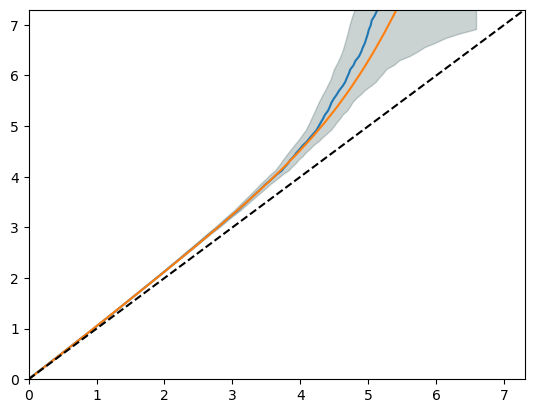

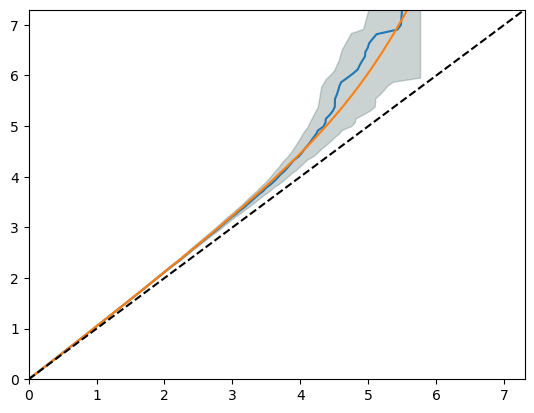


Figure S1. Univariate Q-Q plots of SNPs, showing observed alcohol use disorder top left, cannabis use disorder top right and opioid use disorder bottom. GWAS p-values (in blue) and model prediction (in orange). Grey shading indicating 95% confidence interval.

### Figure S2


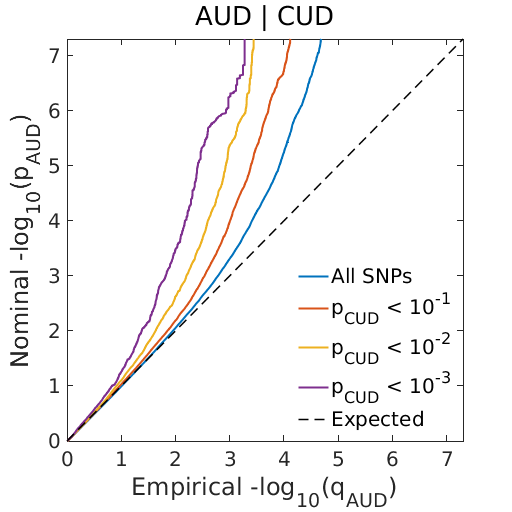

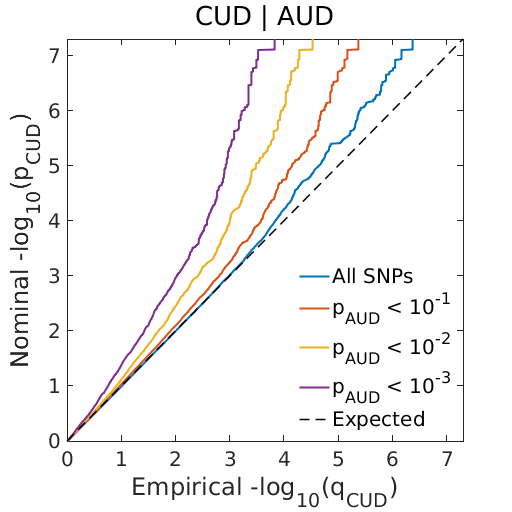

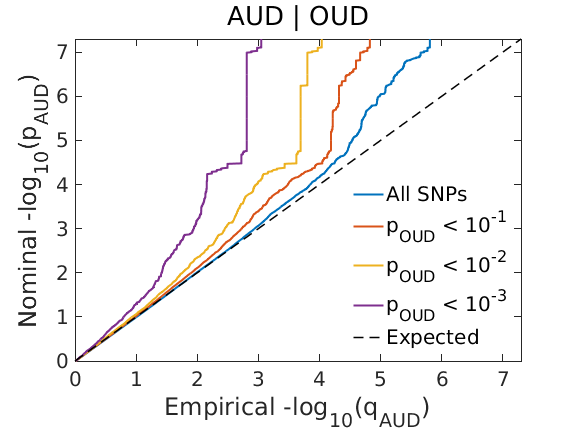

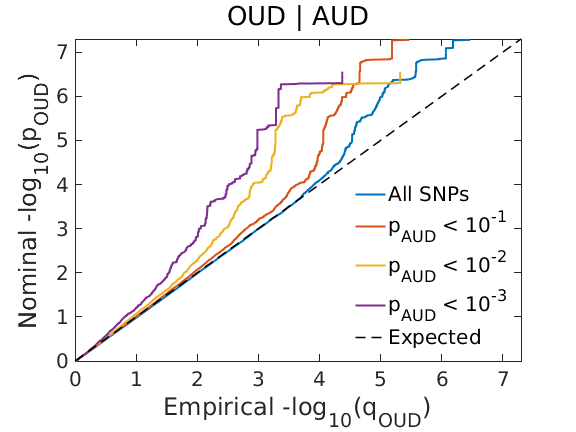

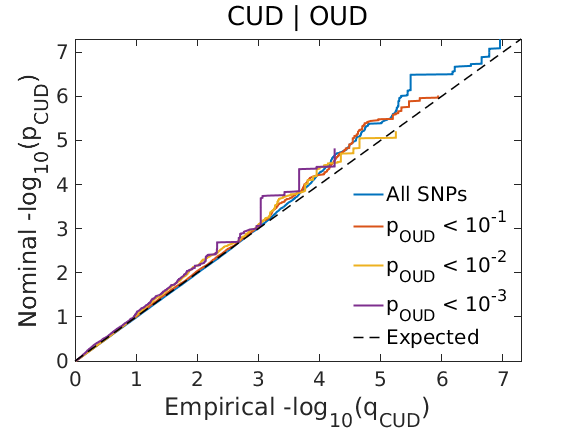

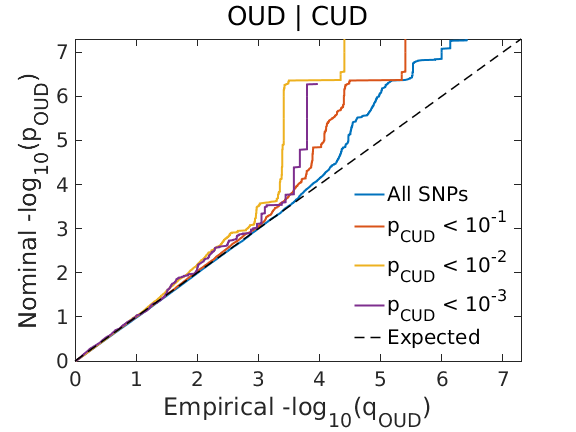


Figure S2. Conditional Q-Q plots of nominal versus empirical −log10 p-values (corrected for inflation) for trait one below the standard GWAS threshold of p<5×10^−8^ as a function of the significance of the association with trait two at the level of p≤0.1, p≤0.01, p≤0.001, respectively. The blue lines illustrate the standard enrichment for all SNPs irrespective of their association p-value in the second phenotype. The dashed line shows the null hypothesis. Successive leftward deflection for declining nominal p-values from the dashed line of no association, indicates that the proportion of non-null SNPs in trait one increase with higher levels of association with trait two, and vice versa.

### Figure S3


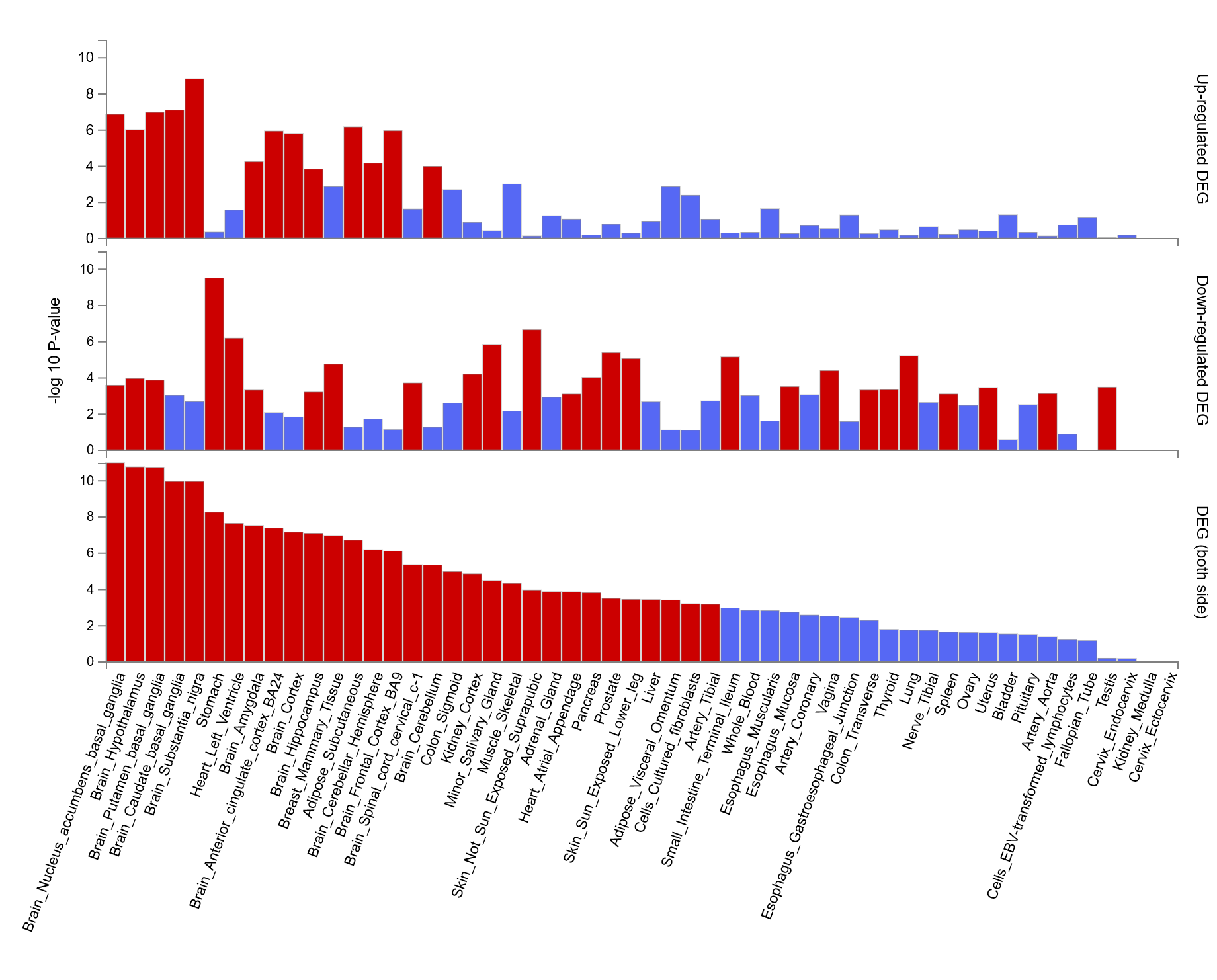


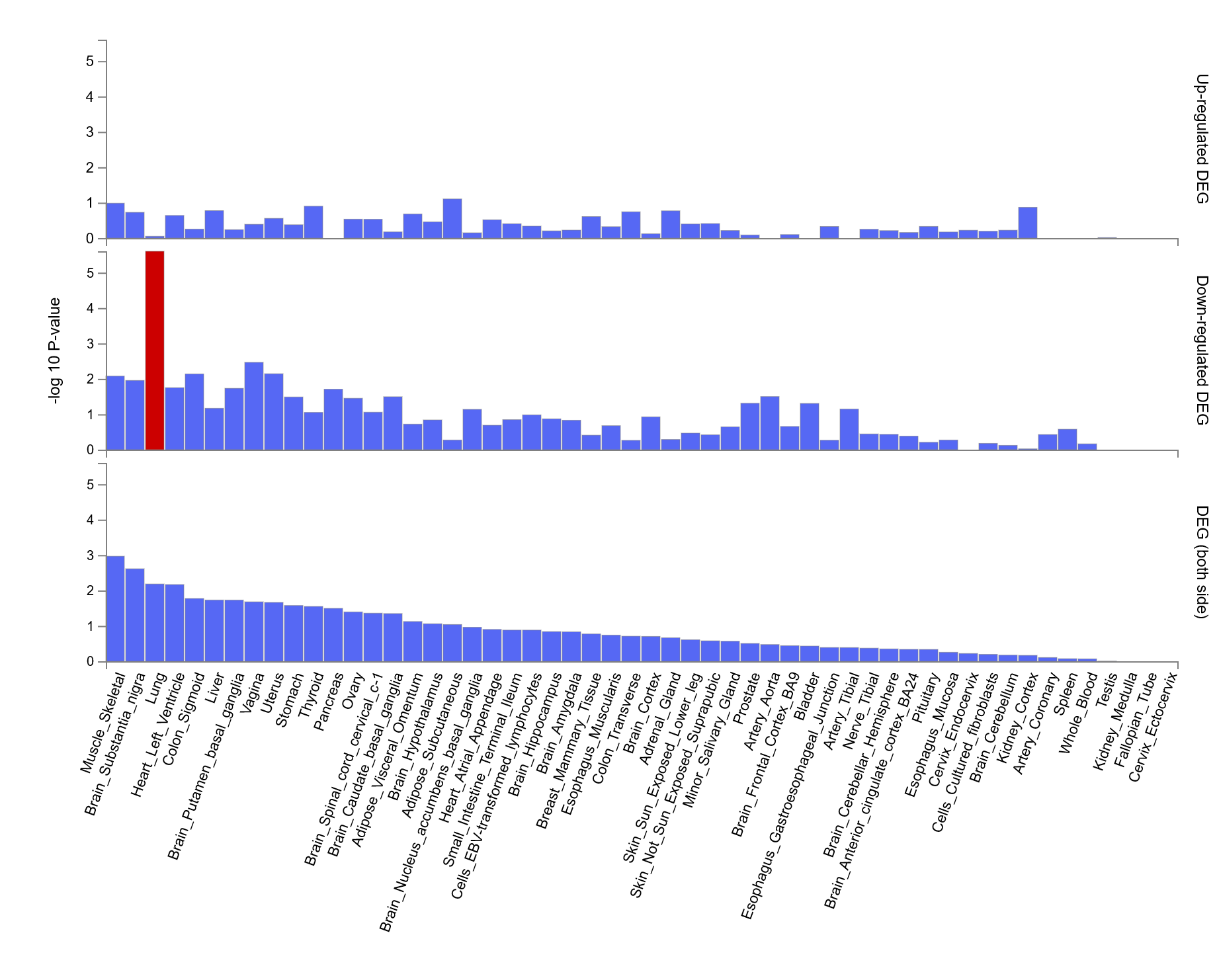


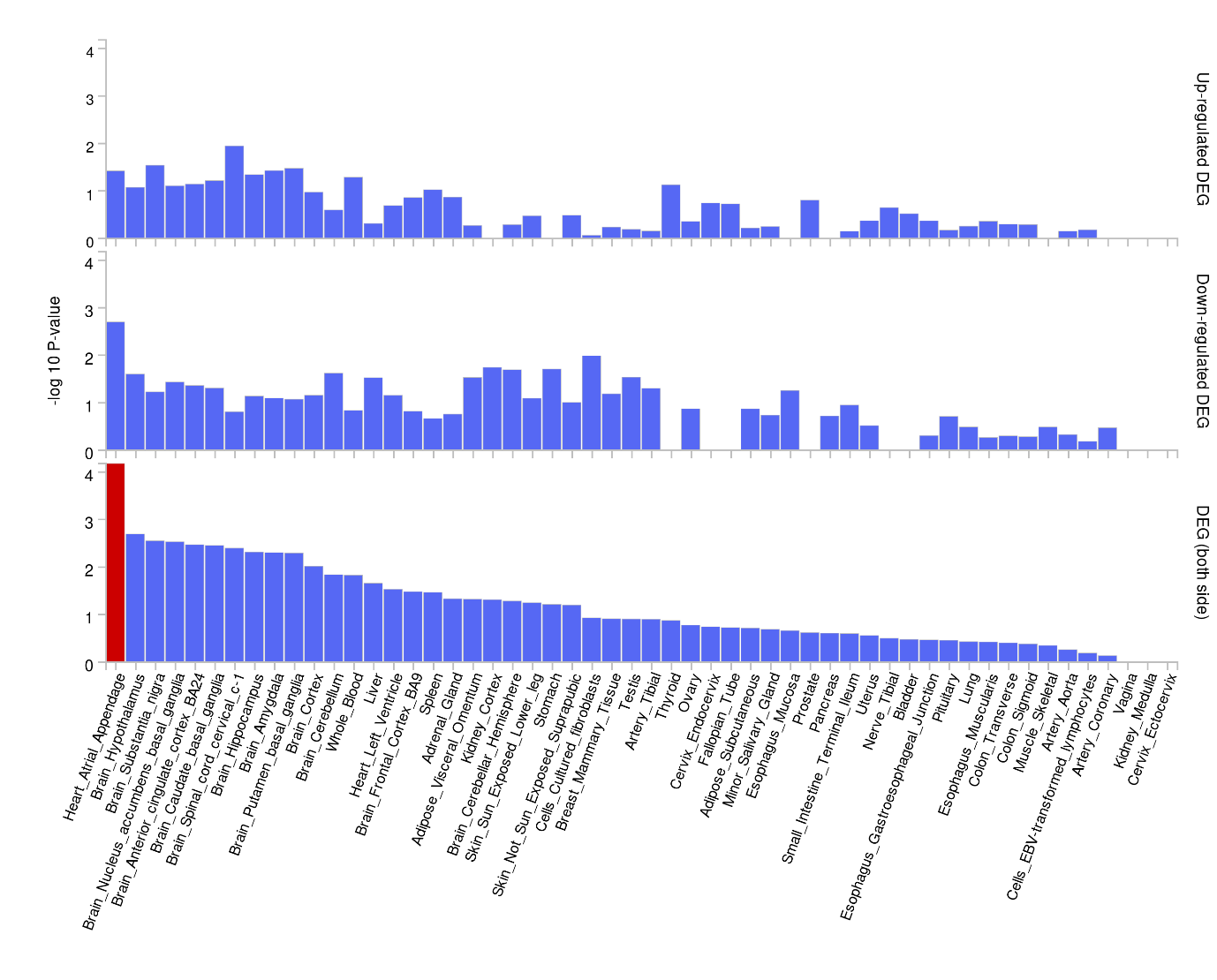


Figure S3. Enrichment in differentially expressed genes in the GTEx v8 54 tissue types. The top panel shows enriched tissue types in alcohol use disorder (AUD) from conditional analysis with cannabis use disorder (CUD) and opioid use disorder (OUD). The middle panel shows tissues enriched in CUD from conditional analysis with AUD and the bottom panel shows enriched tissues in OUD conditioned on AUD.

### Figure S4


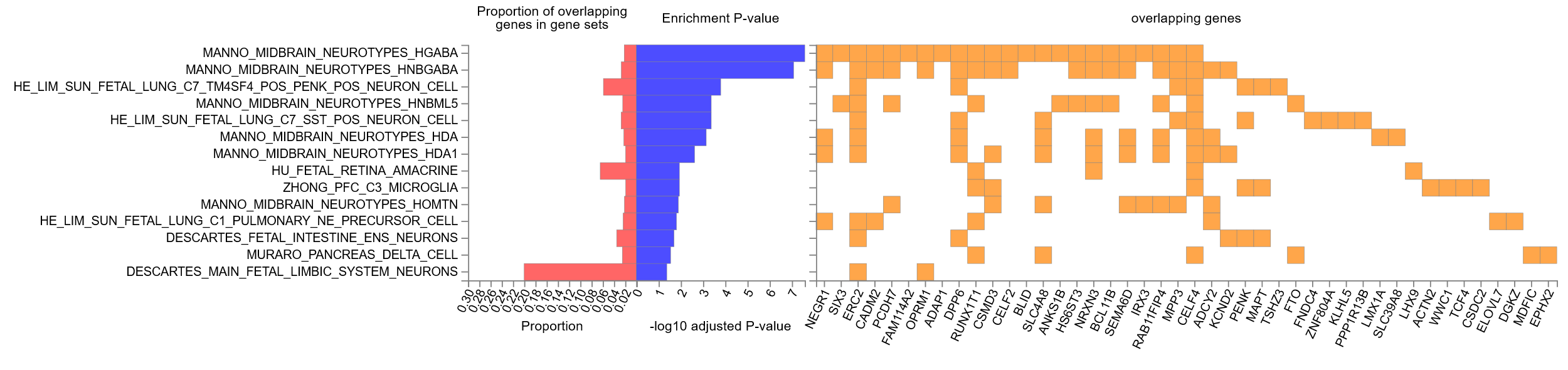


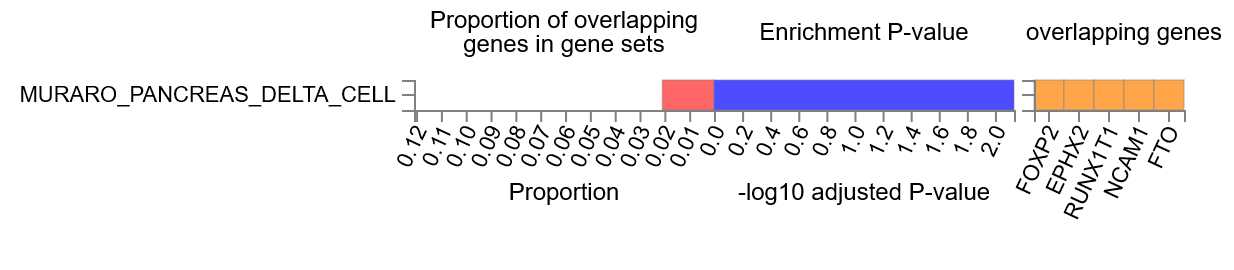


Figure S4. Cell type expression signatures from conjunctional analyses of alcohol use disorder genes (top panel) and of cross-substance use disorder genes (bottom panel).

### Figure S5


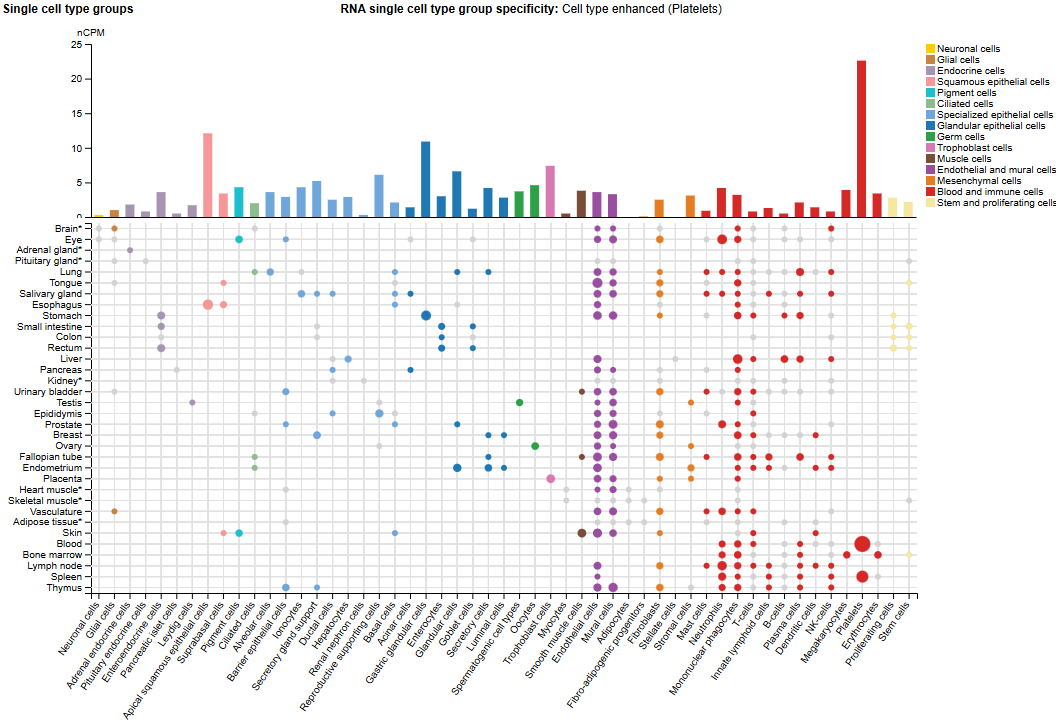

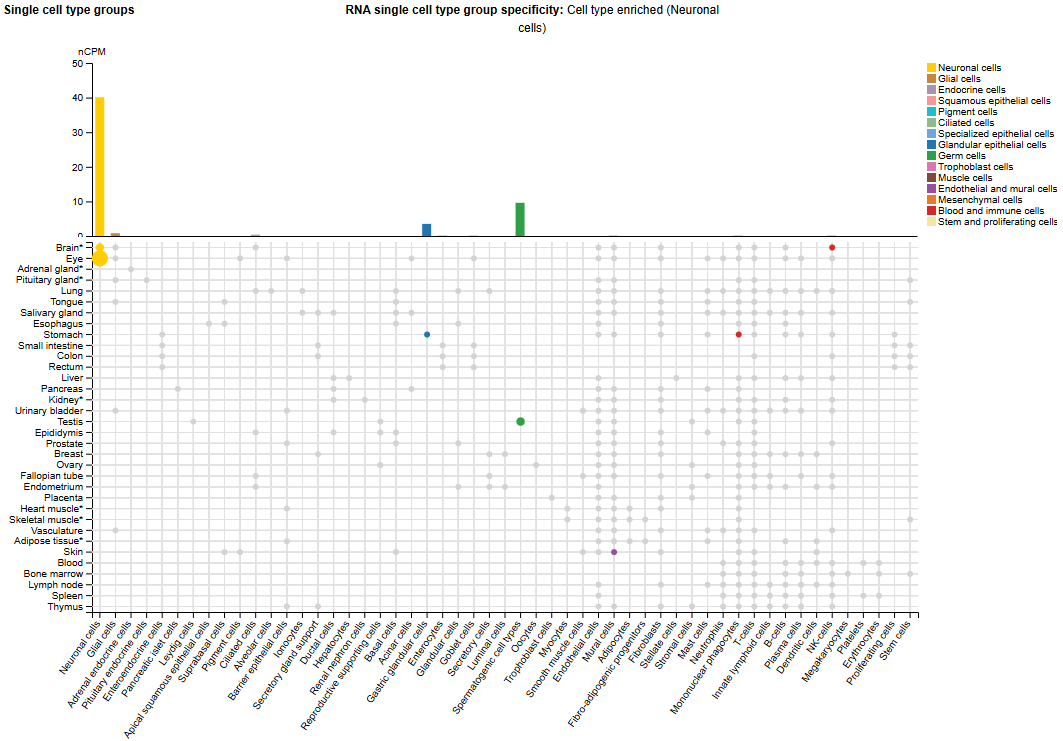


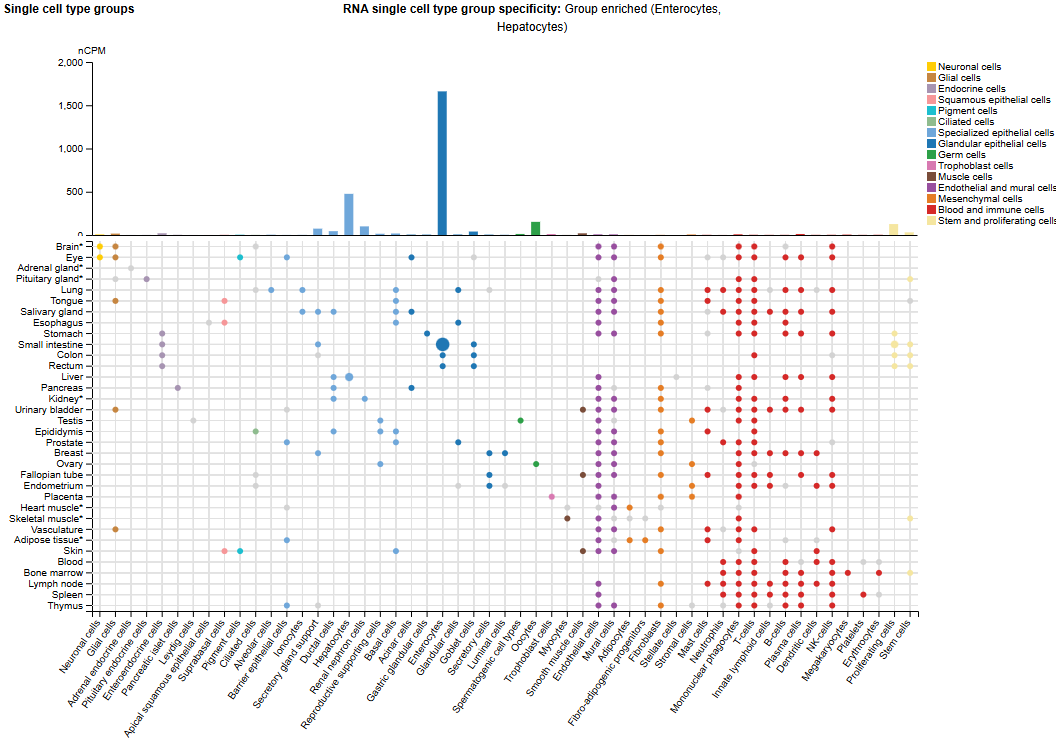

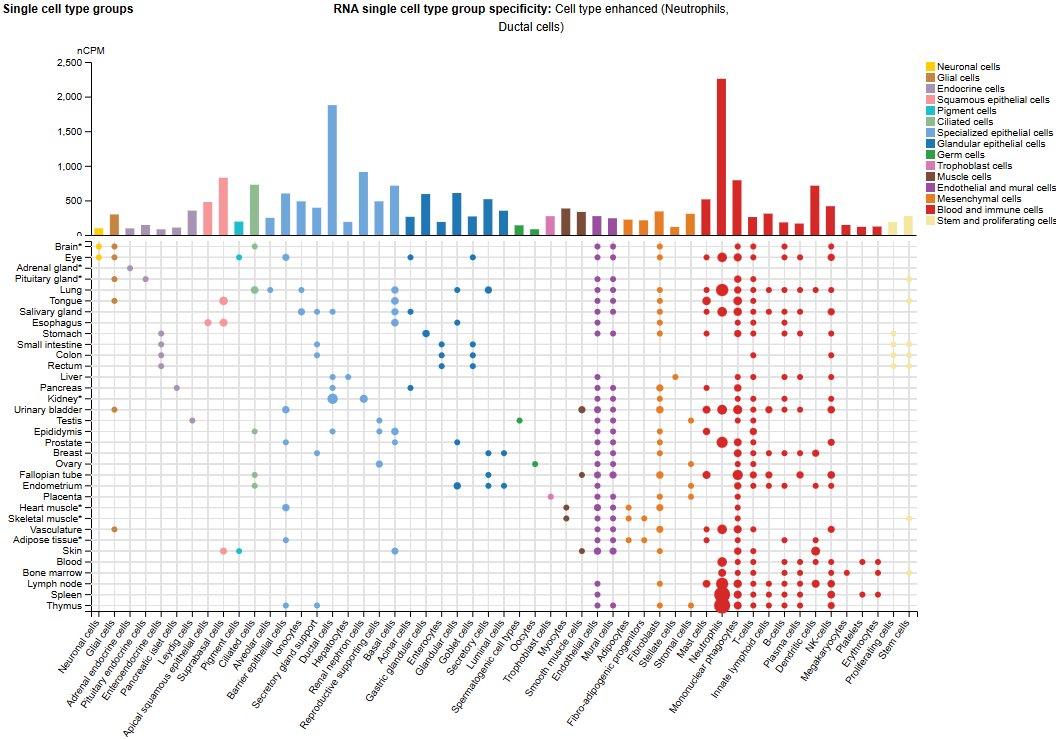


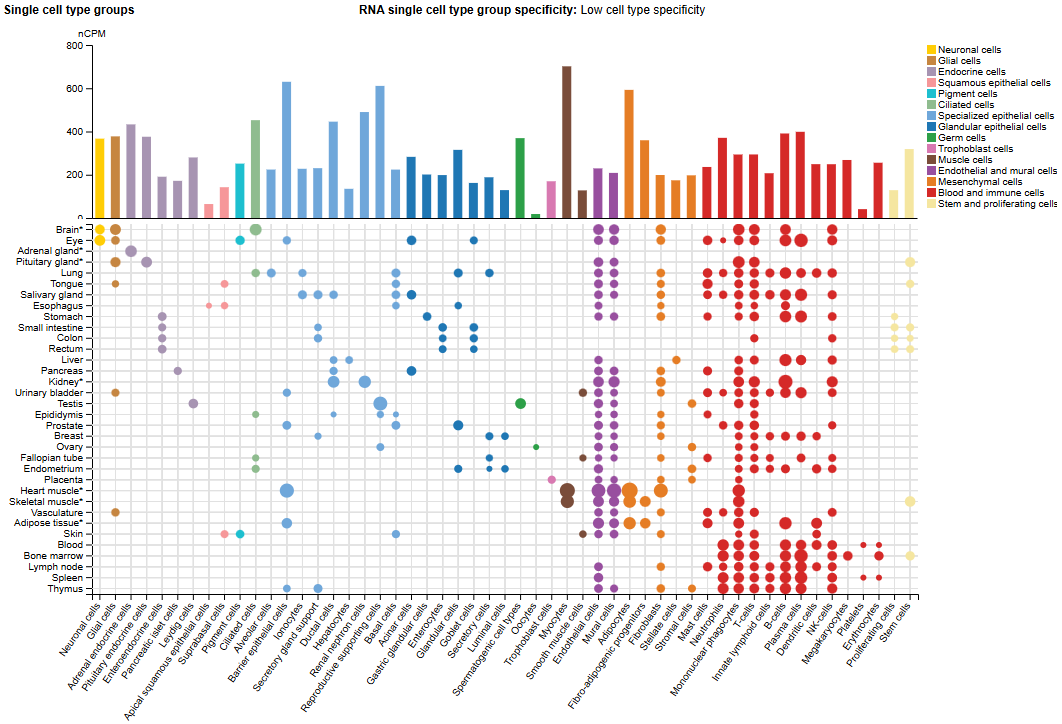


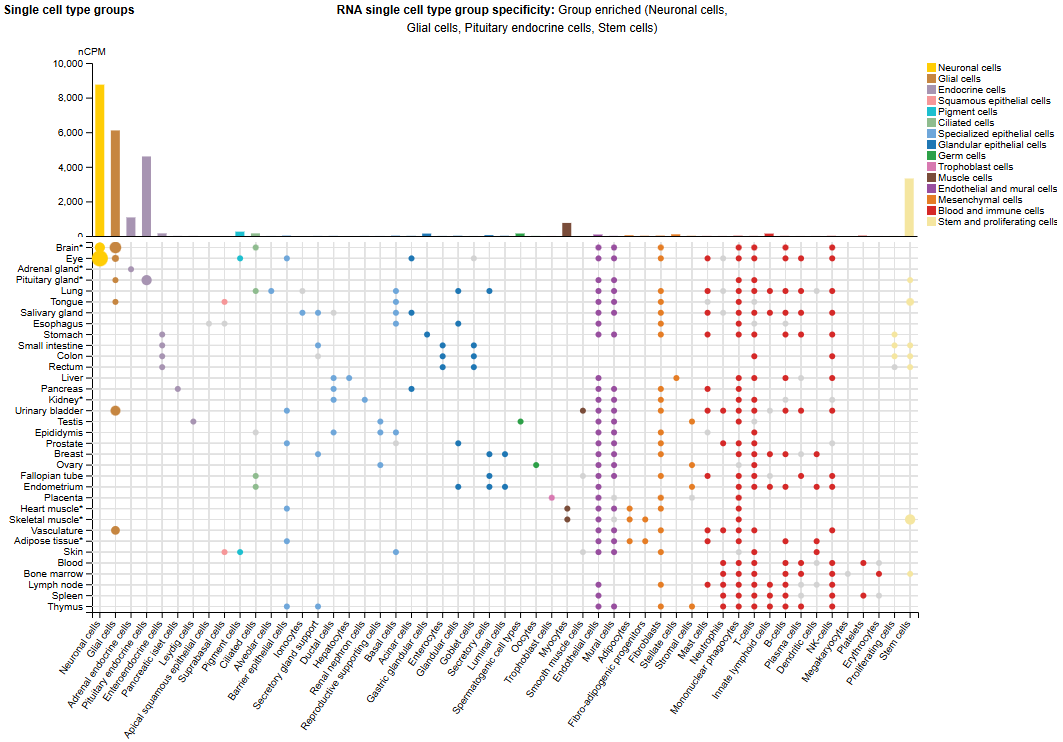


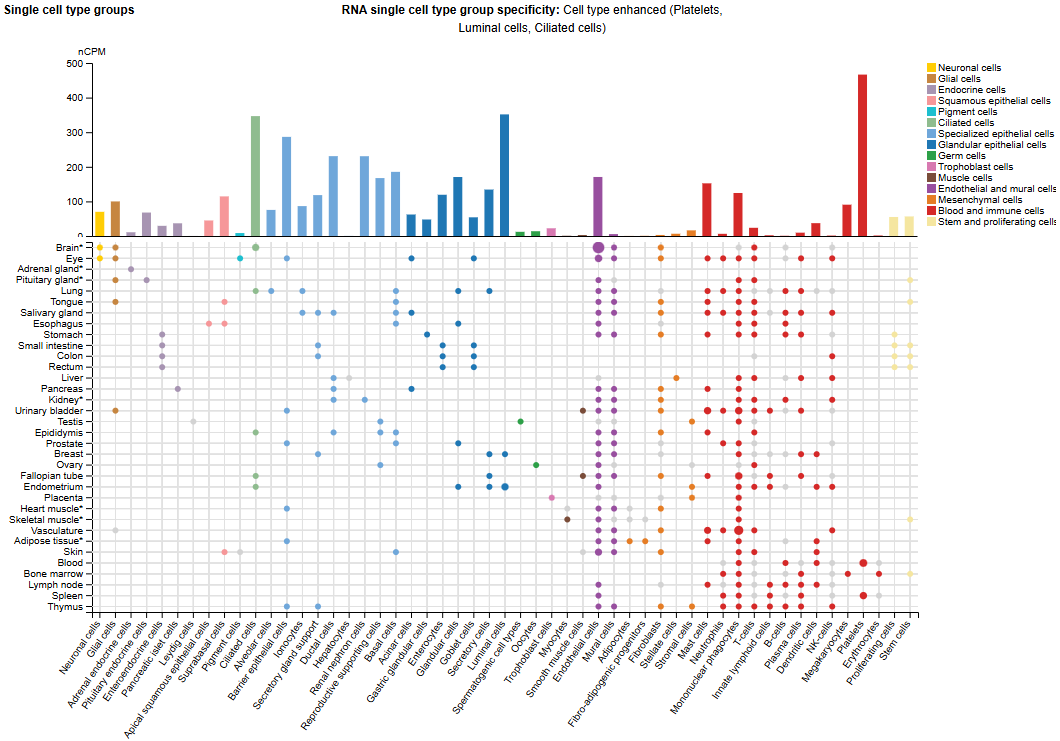


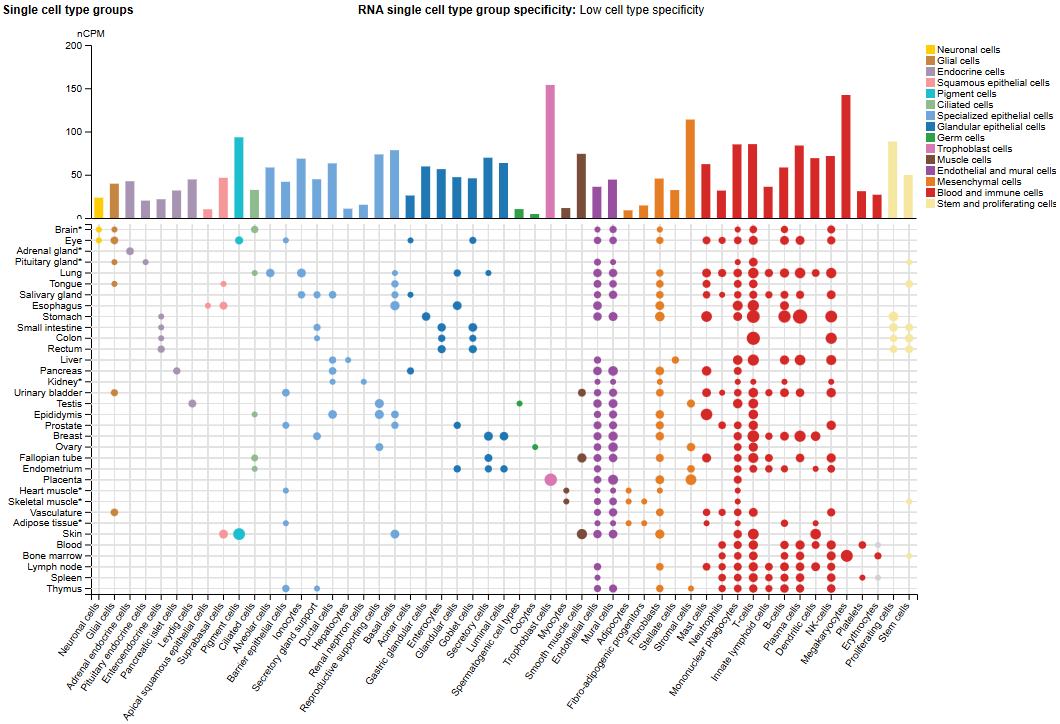


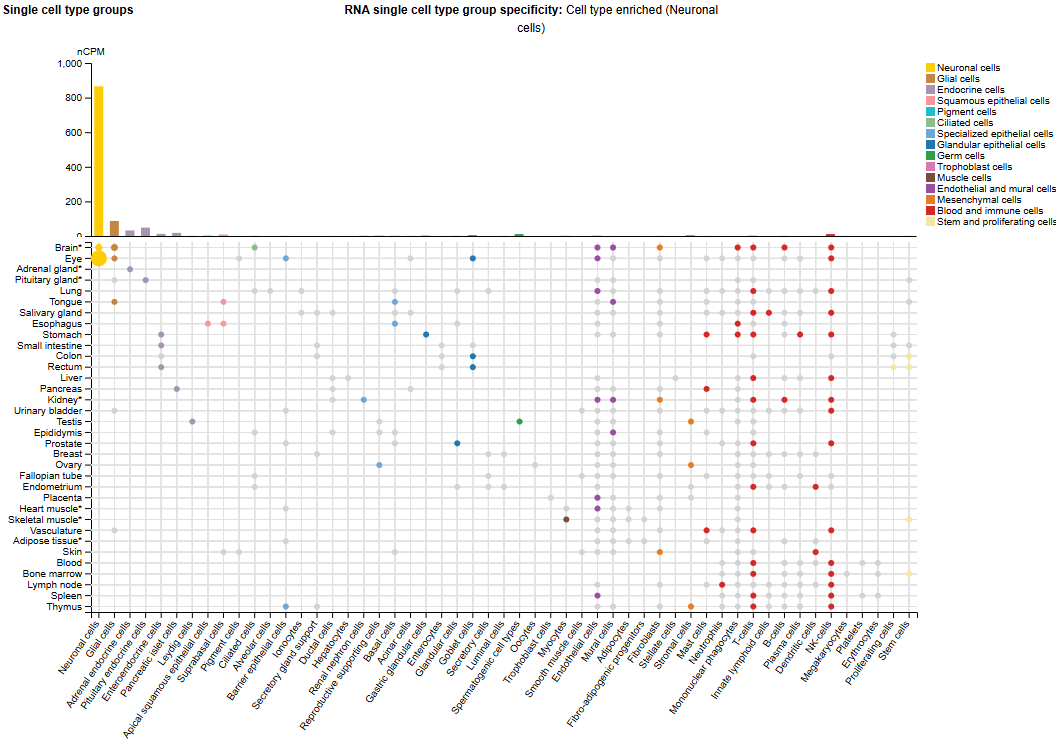


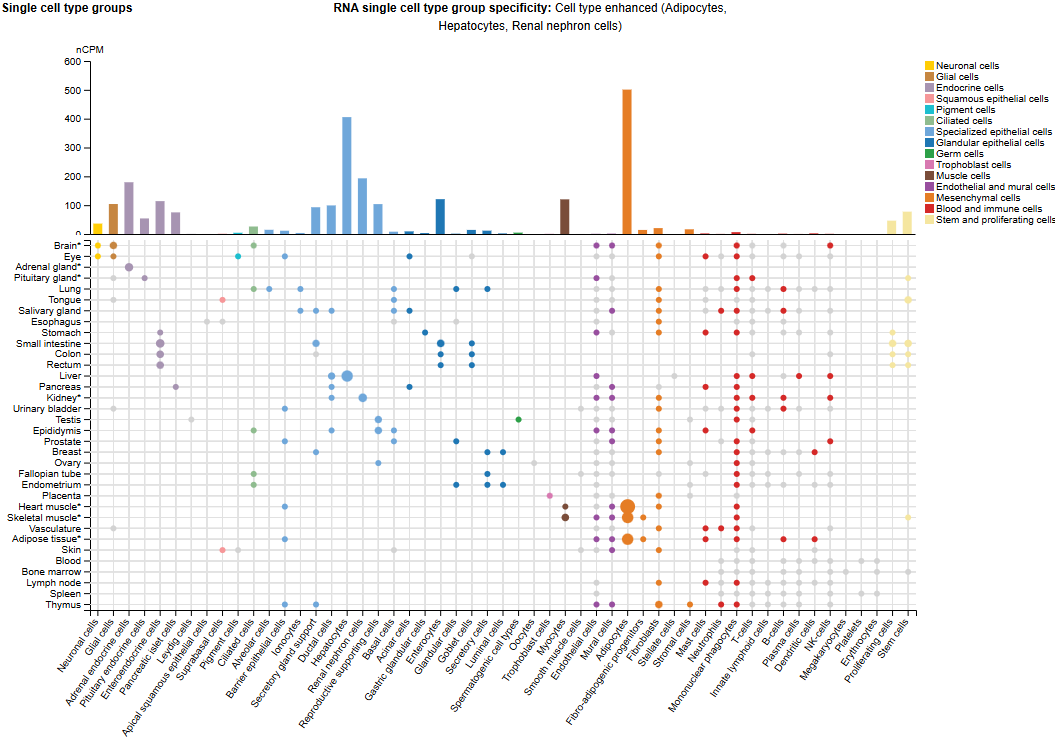


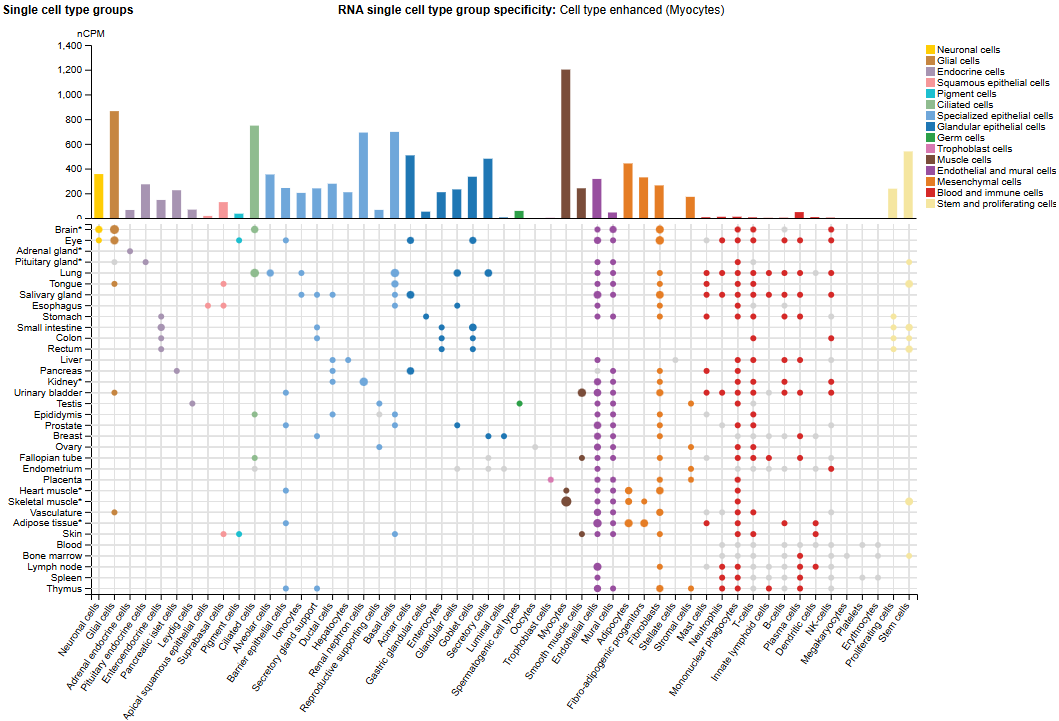


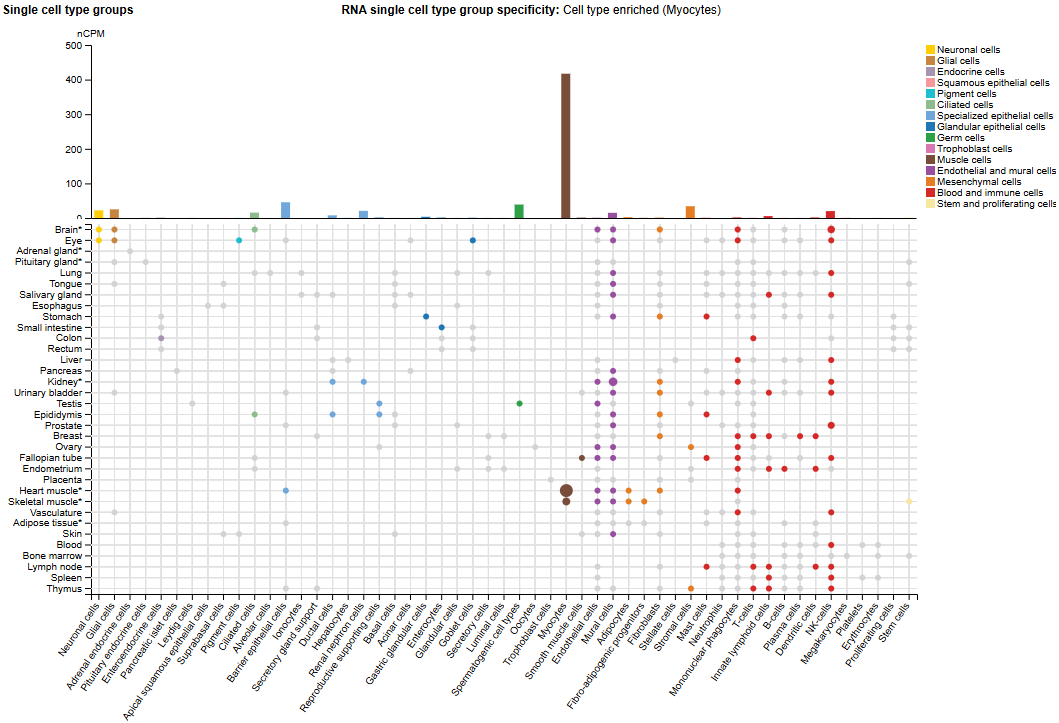


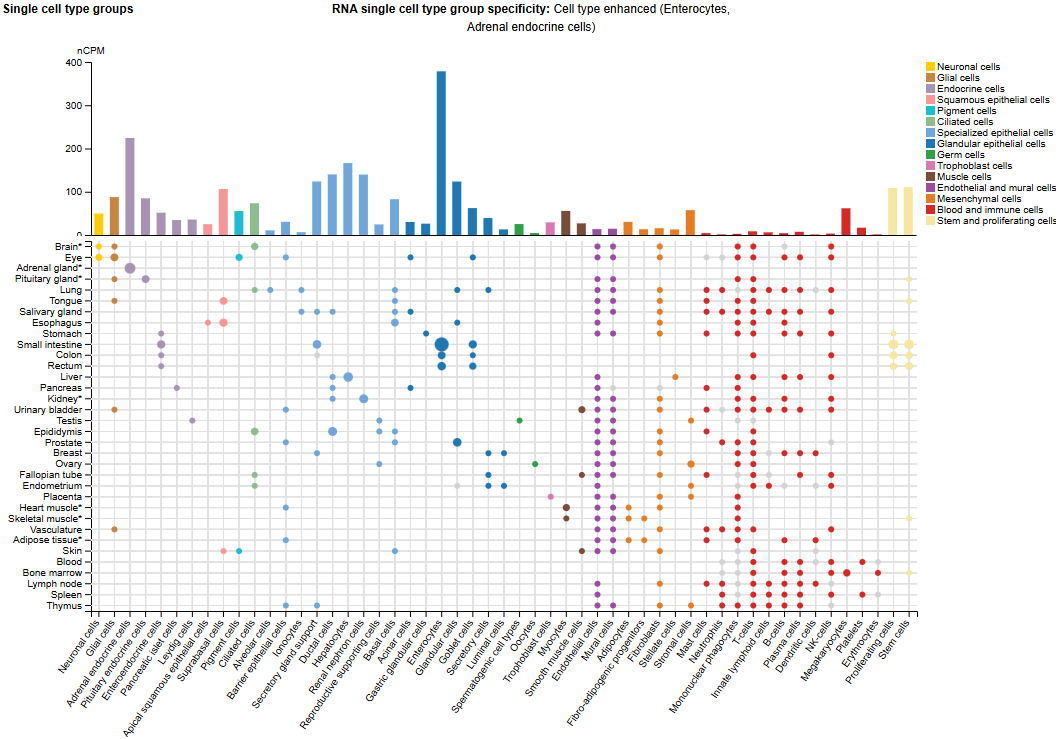


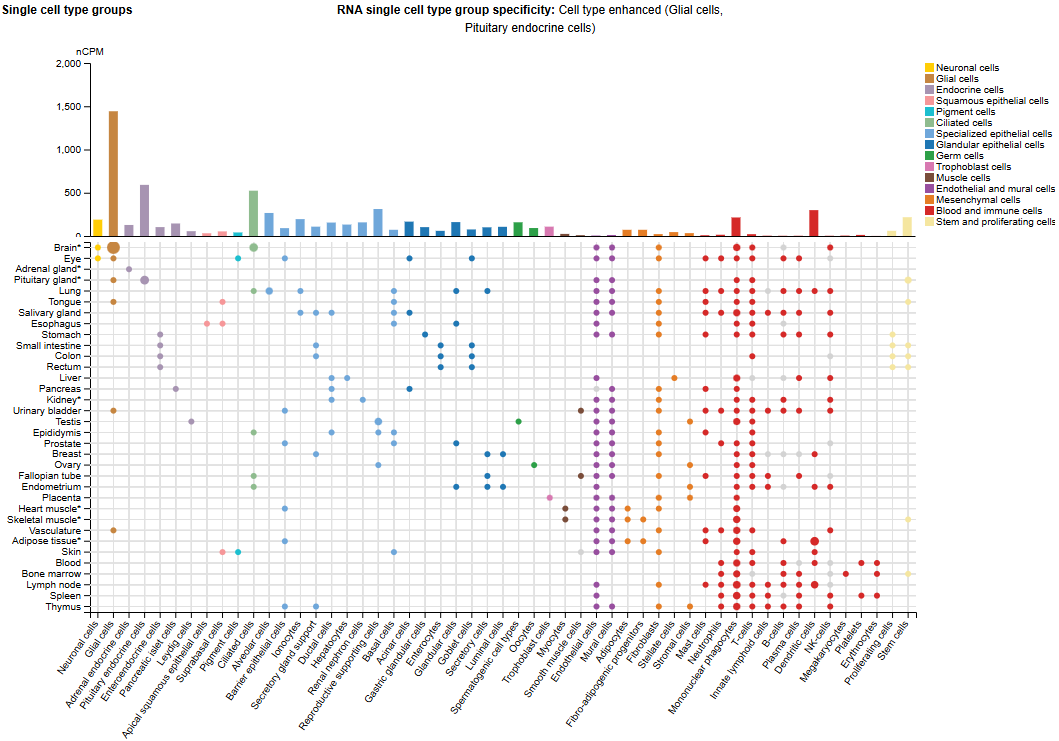


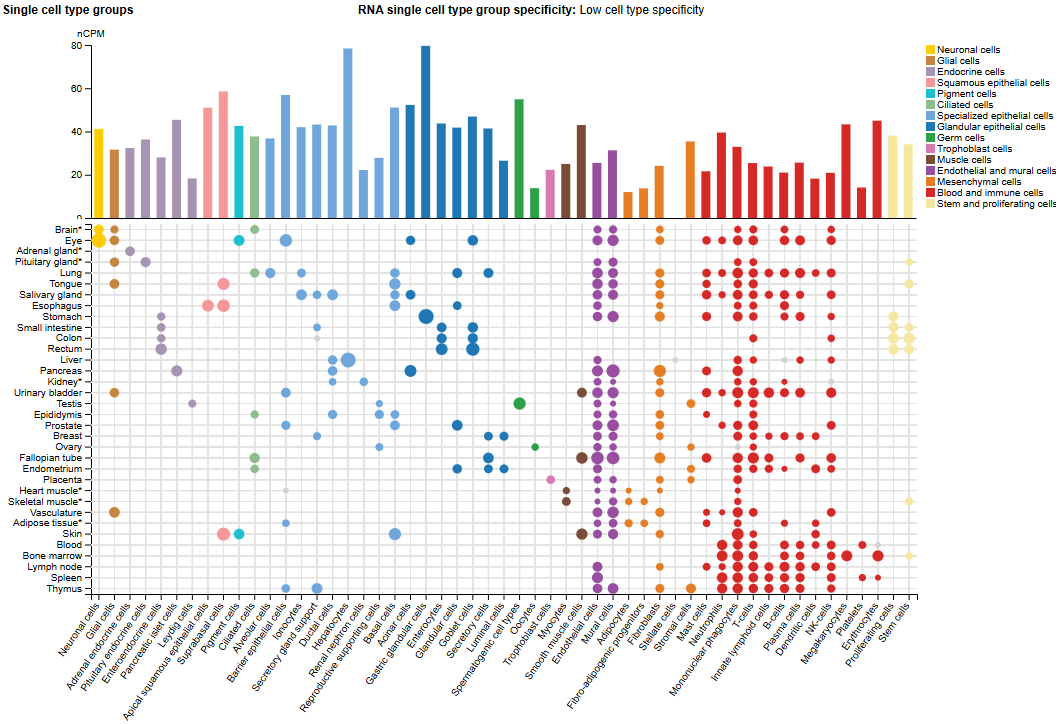


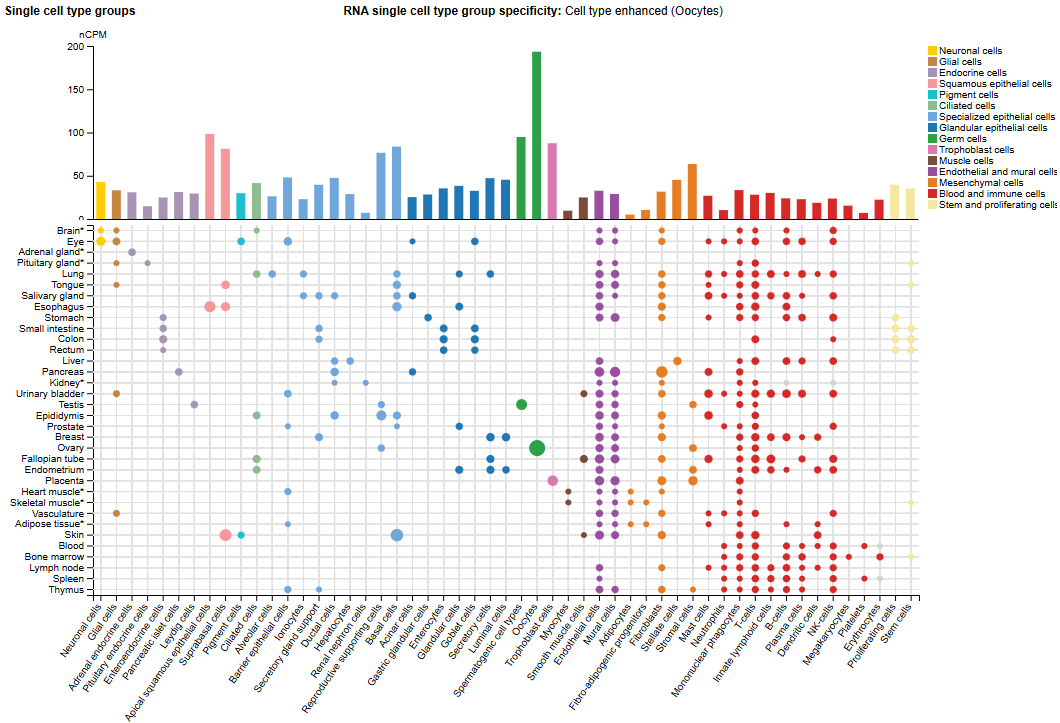


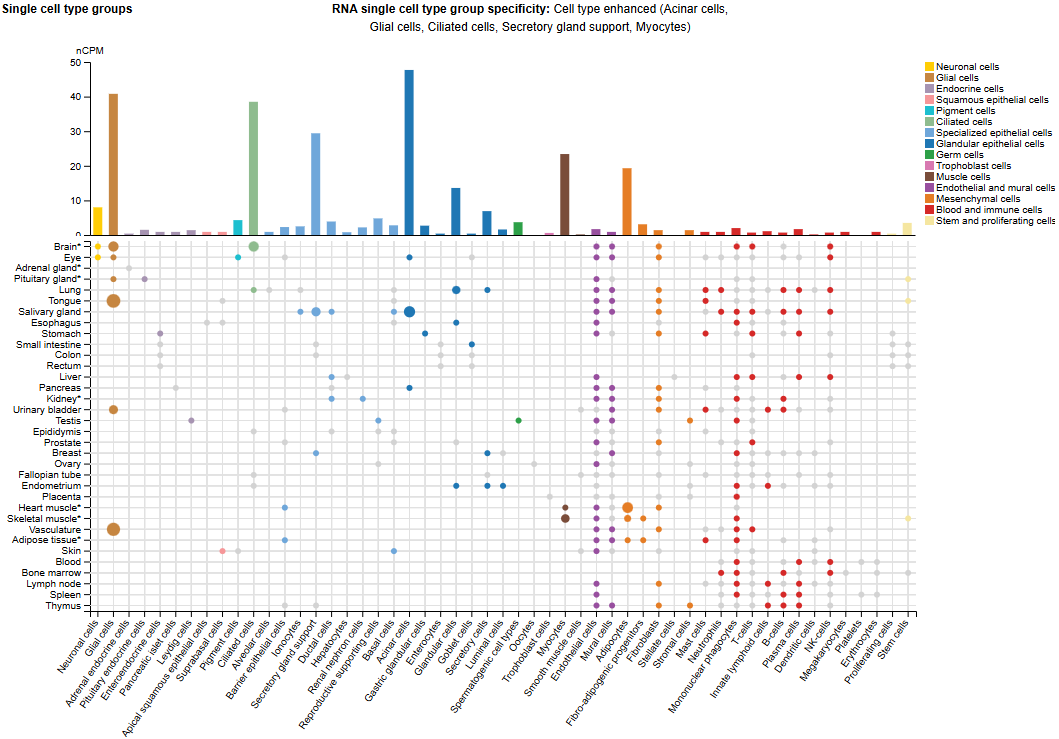


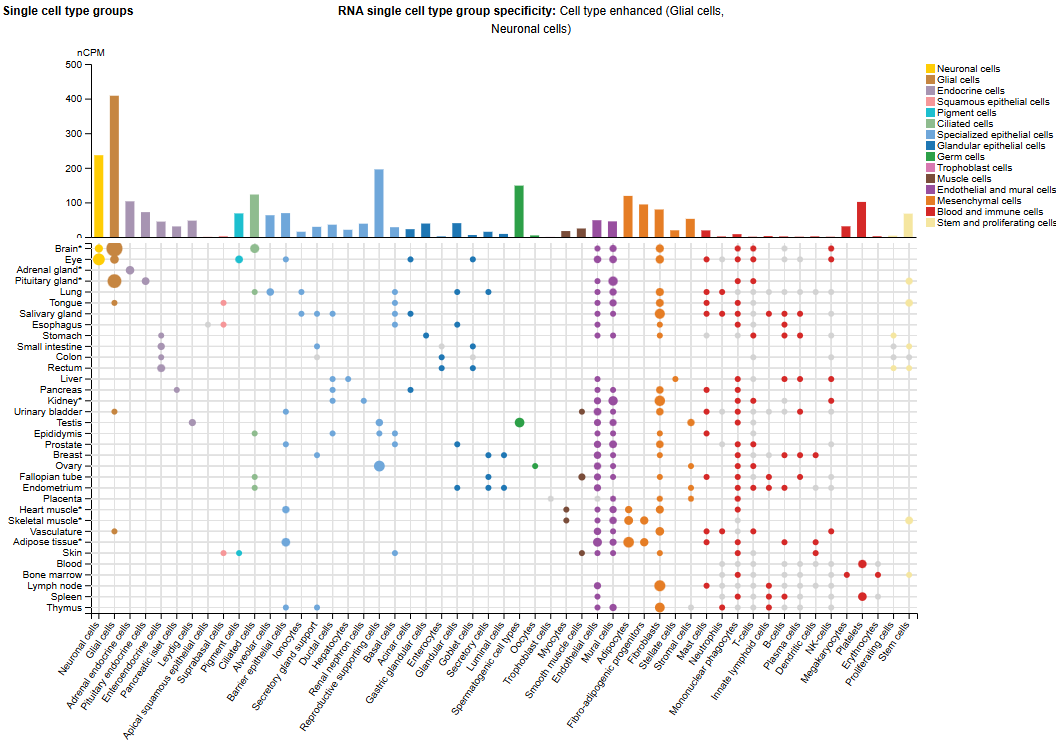


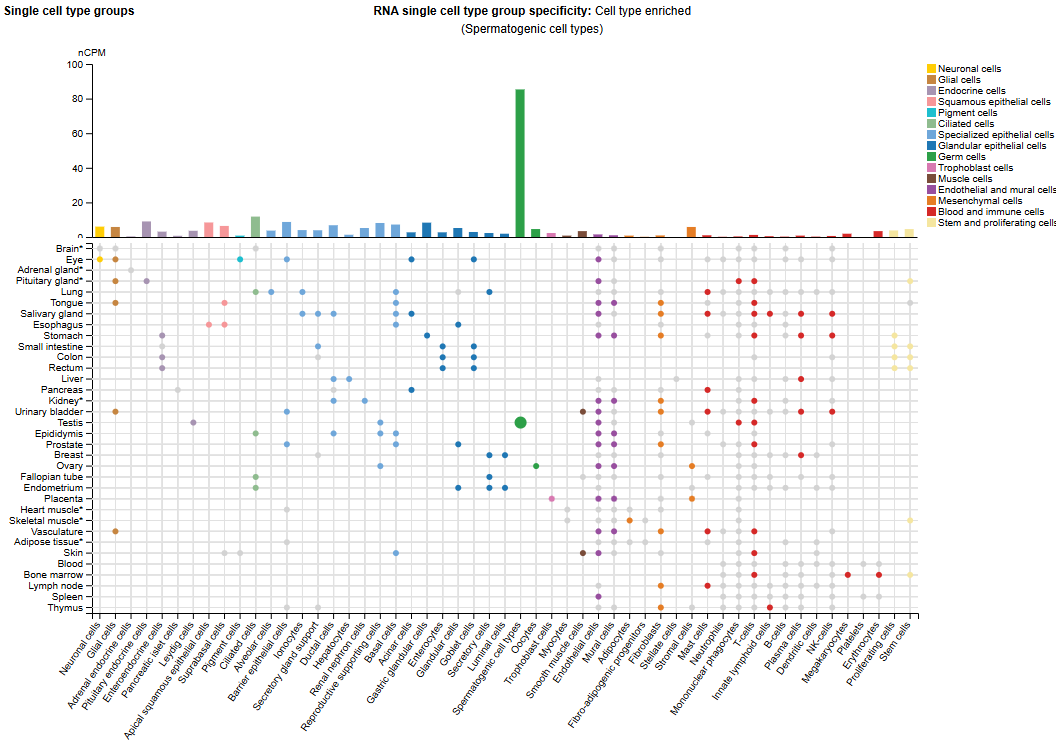

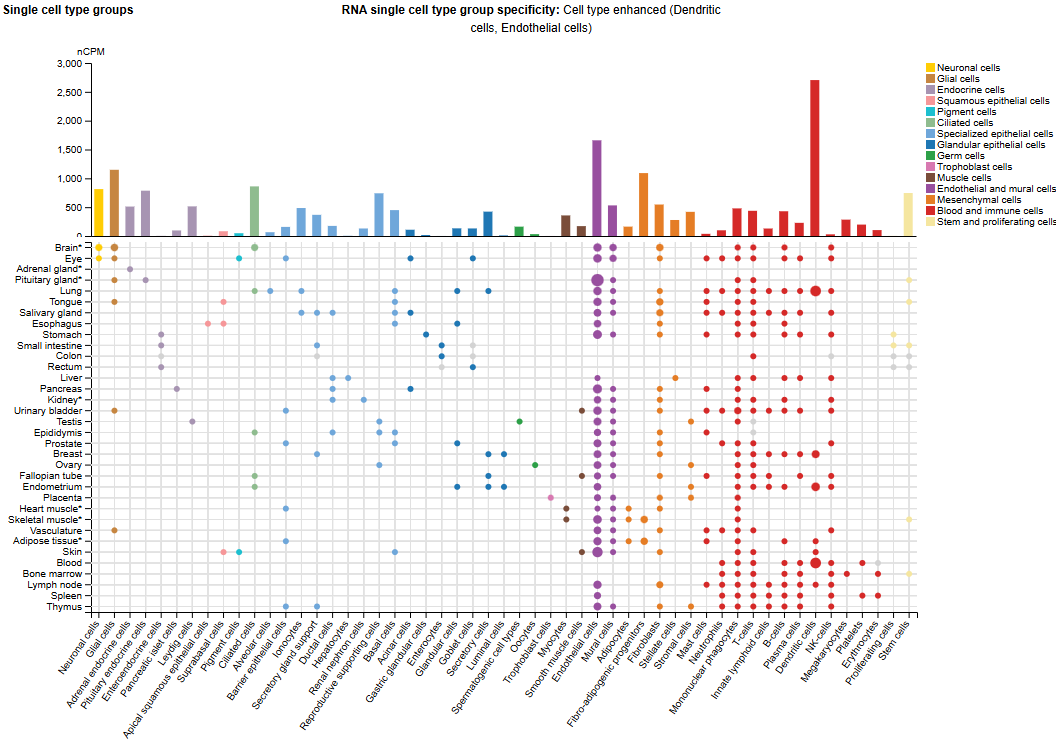

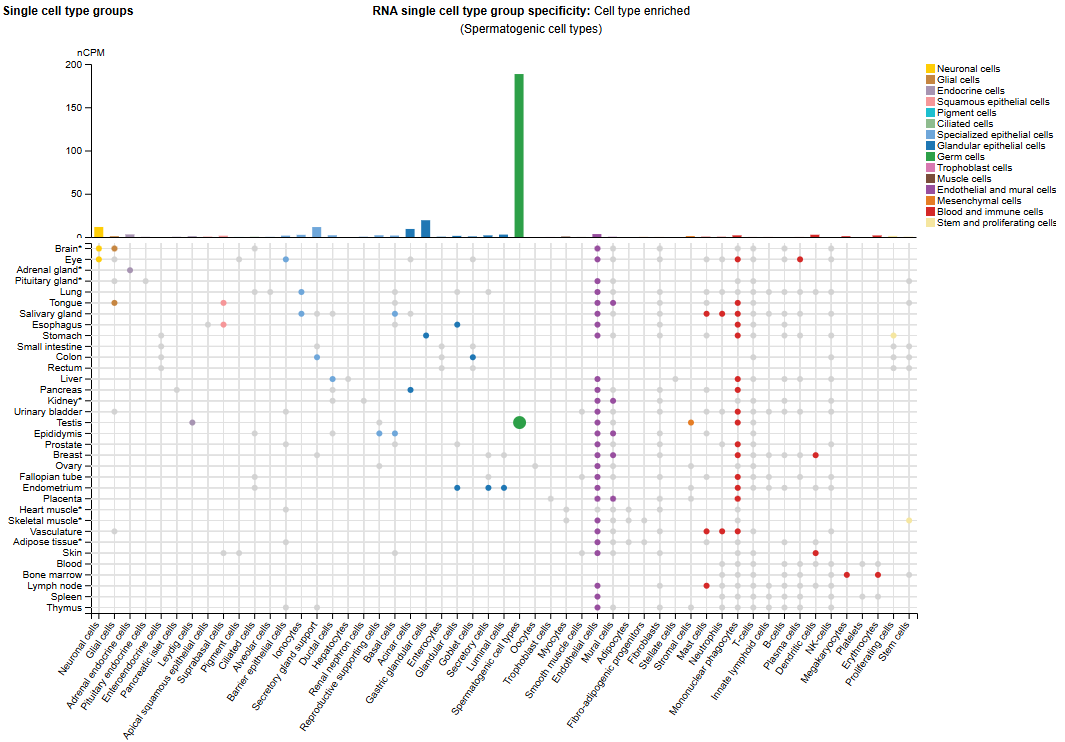


Figure S5. Tissue and cell expression of genes in conjunctional analysis of alcohol use disorder with cannabis use disorder. From top panel *LEPROT, BARHL2, KHK, NFE2L2, RBM6, CADM2, ELOVL7, HMGN4, LRFN2, MLXIPL, FOXP2, MYOM2, EPHX2, SHTN1, LIN7C, MED19, TMPRSS5, WASF3, GOLGA6L10, TCF4, IZUMO1.*

### Figure S6


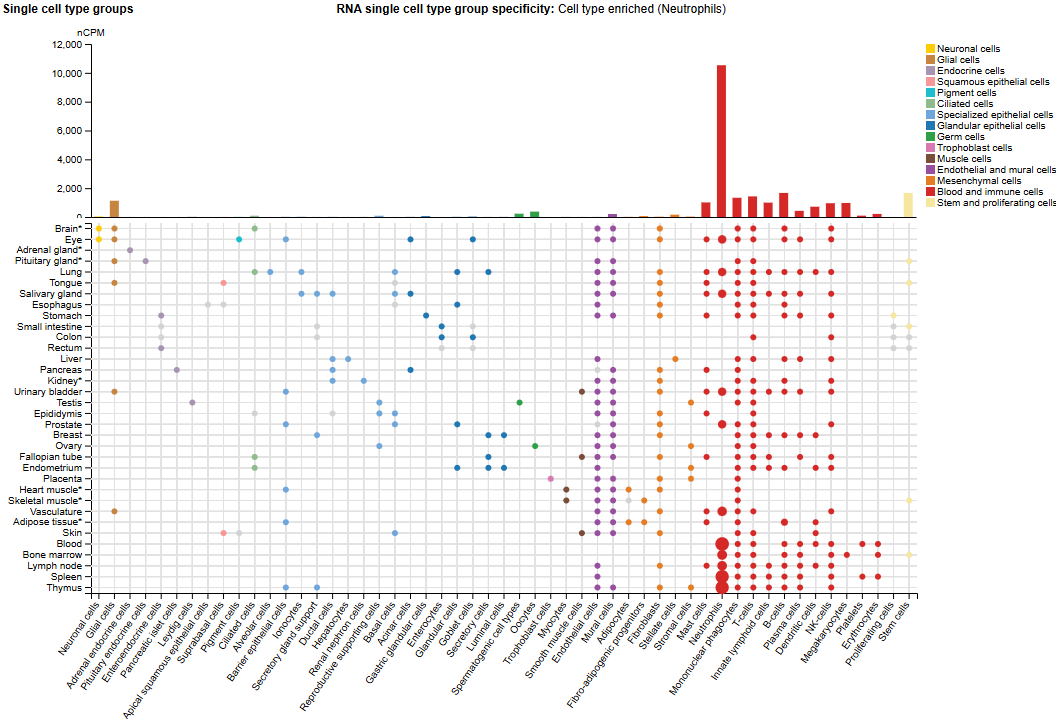

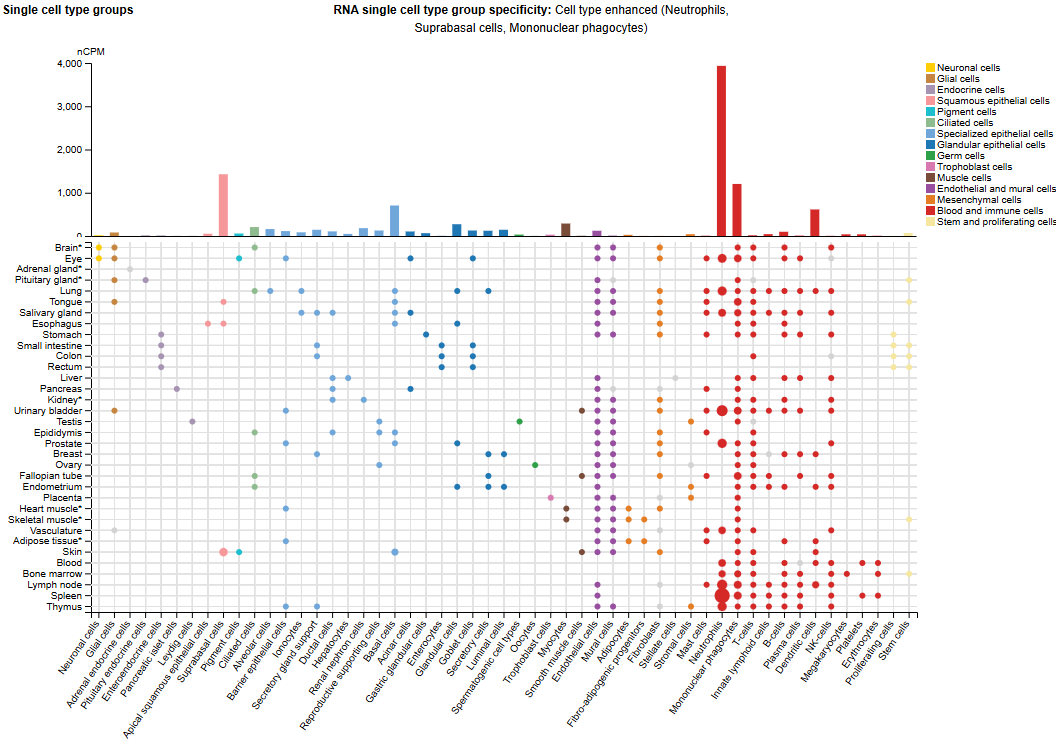

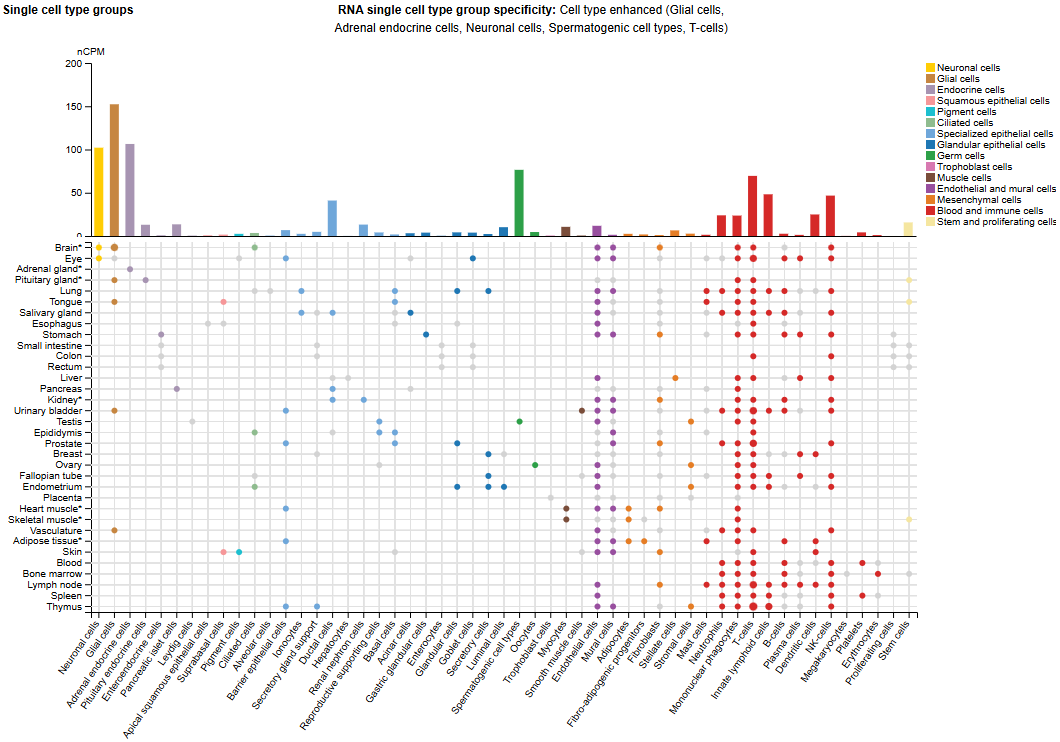

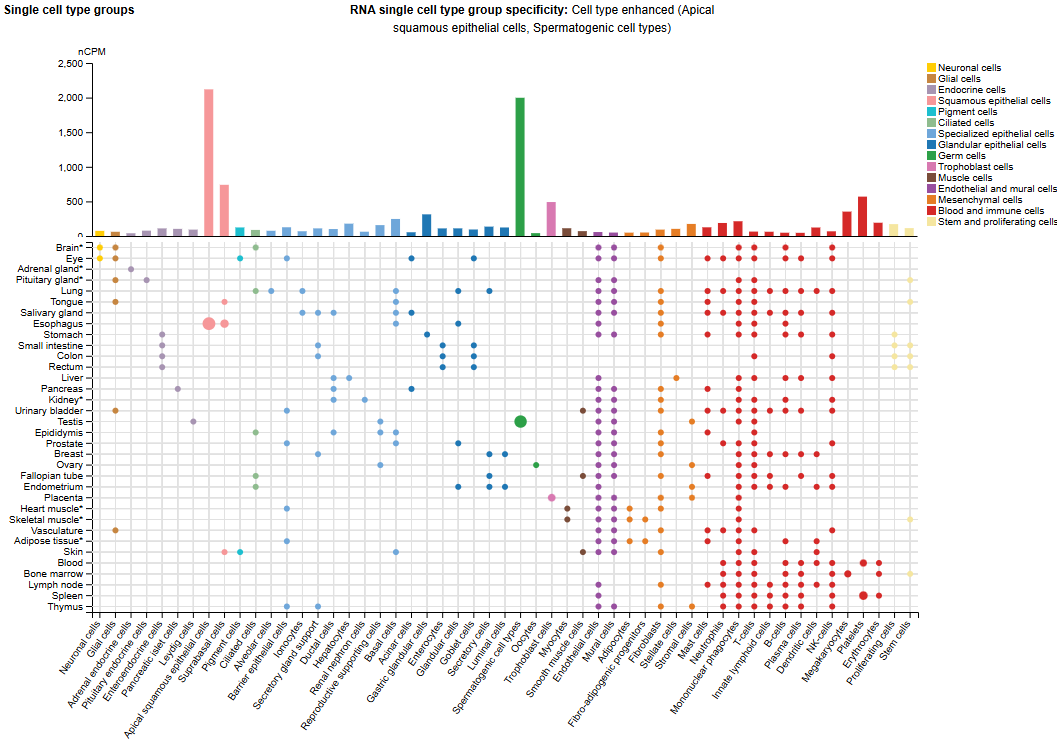

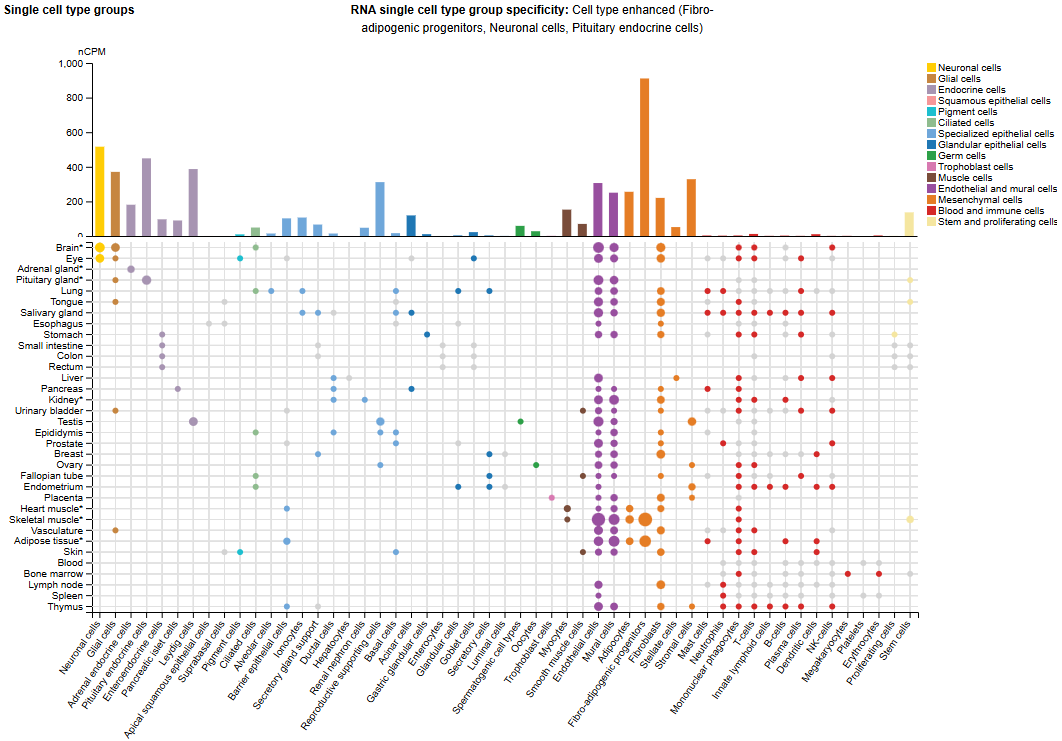

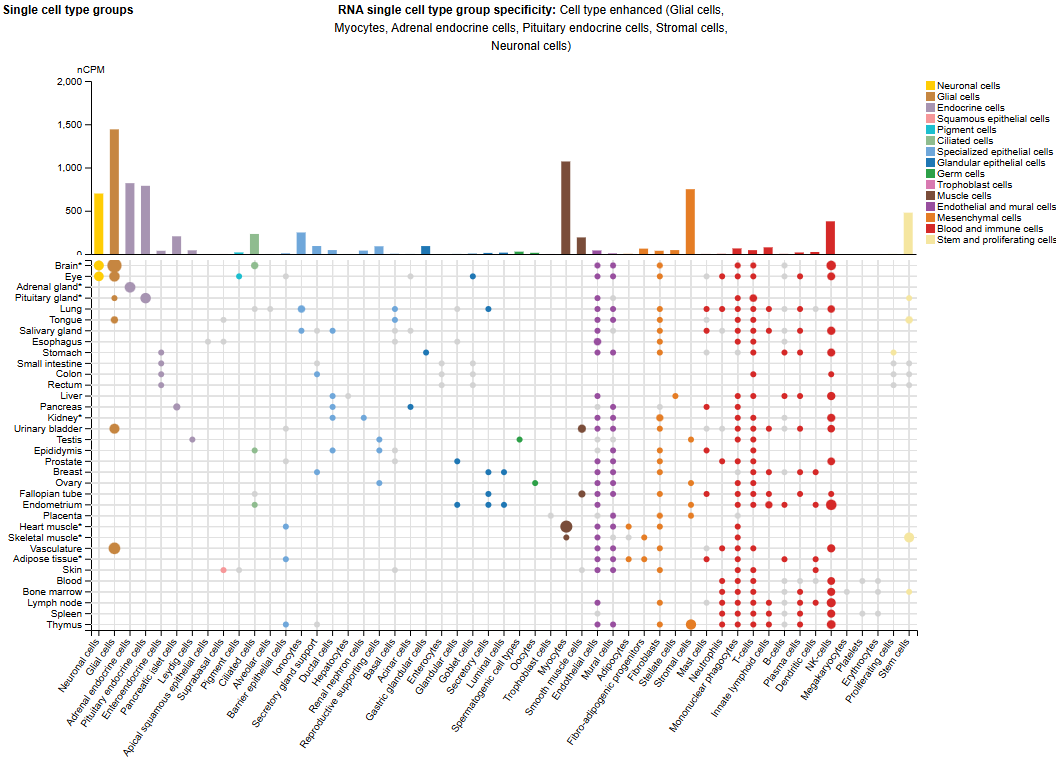


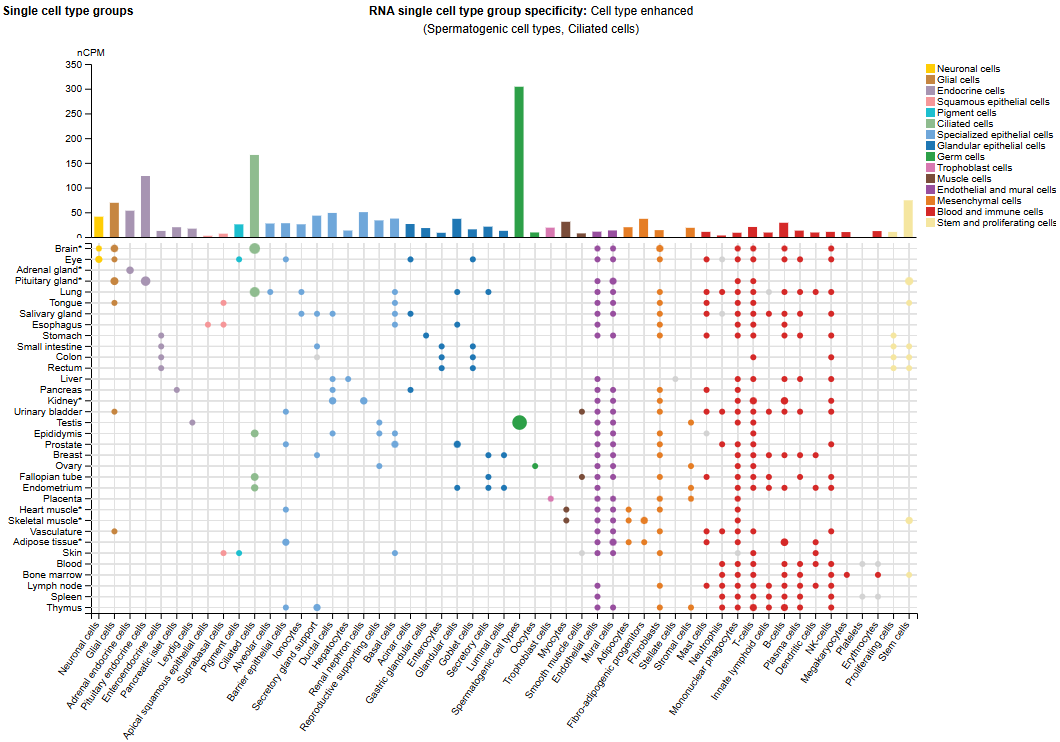

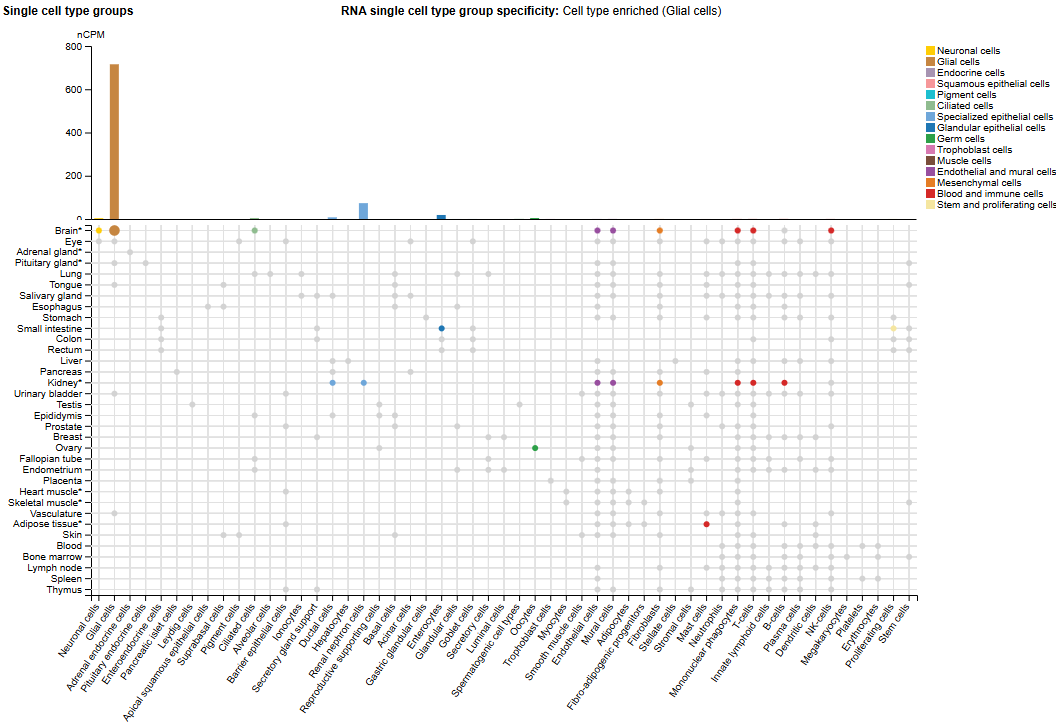

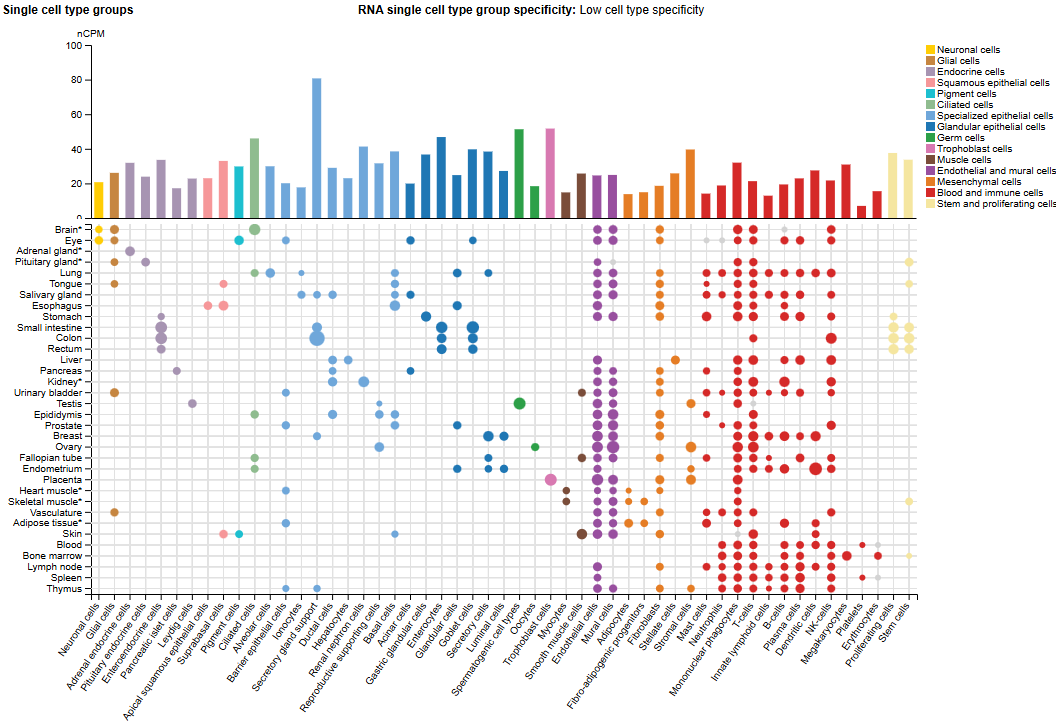

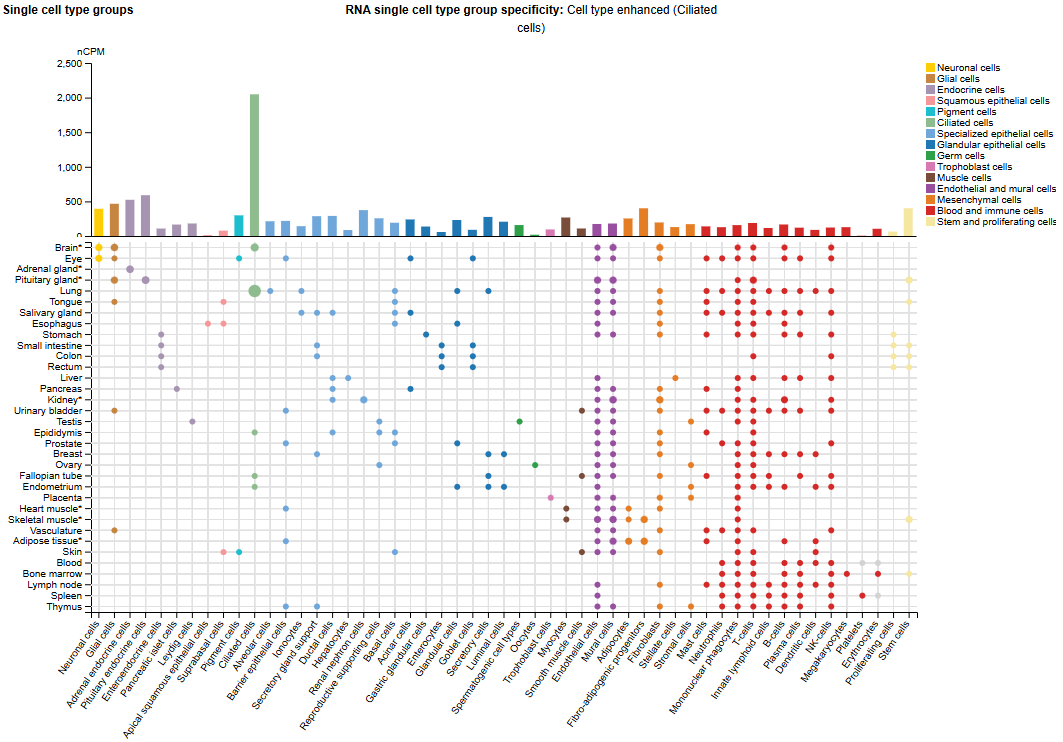


Figure S6. Tissue and cell expression of genes in conjunctional analysis of alcohol use disorder with opioid use disorder. From top panel ARHGAP15, RNF144B, OPRM1, STMP1, RUNX1T1, NCAM1, TTC12, SLC5A11, INO80E, FTO.

### References

1. Icick R, Shadrin A, Holen B, et al. Identification of risk variants and cross-disorder pleiotropy through multi-ancestry genome-wide analysis of alcohol use disorder. *Nature Mental Health*. Published online January 8, 2025. doi:10.1038/s44220-024-00353-8

2. Zhou H, Kember RL, Deak JD, et al. Multi-ancestry study of the genetics of problematic alcohol use in over 1 million individuals. *Nature Medicine*. 2023;29(12):3184-3192. doi:10.1038/s41591-023-02653-5

3. Kurki MI, Karjalainen J, Palta P, et al. FinnGen provides genetic insights from a well-phenotyped isolated population. *Nature*. 2023;613(7944):508-518. doi:10.1038/s41586-022-05473-8

4. Levey DF, Galimberti M, Deak JD, et al. Multi-ancestry genome-wide association study of cannabis use disorder yields insight into disease biology and public health implications. *Nature Genetics*. 2023;55(12):2094-2103. doi:10.1038/s41588-023-01563-z

5. Johnson EC, Demontis D, Thorgeirsson TE, et al. A large-scale genome-wide association study meta-analysis of cannabis use disorder. *The Lancet Psychiatry*. 2020;7(12):1032-1045. doi:10.1016/S2215-0366(20)30339-4

6. Kember RL, Vickers-Smith R, Xu H, et al. Cross-ancestry meta-analysis of opioid use disorder uncovers novel loci with predominant effects in brain regions associated with addiction. *Nature Neuroscience*. 2022;25(10):1279-1287. doi:10.1038/s41593-022-01160-z
