# Supplementary material for: Shared genetic variants across substance use disorders implicate common neurobiological pathways, a genome‐wide mixed methods study
Source: Gen Psychiatr. 2026 Apr 20;39(2):e70017. doi: 10.1002/gps3.70017 (PMC13095382; doi:10.1002/gps3.70017)
Supplement: Supplementary file 2 — Supporting Information S2 [file GPS3-39-e70017-s002.docx]

| **Index** | |
| --- | --- |
| Table S1 | Univariate MiXeR estimates for AUD, CUD and OUD |
| Table S2 | Model fit for bivariate AUD, CUD and OUD analysis with MiXeR |
| Table S3 | Loci associated with AUD conditional on CUD at condFDR<0.01 |
| Table S4 | Loci associated with CUD conditional on AUD at condFDR<0.01 |
| Table S5 | Loci associated with AUD conditional on OUD at condFDR<0.01 |
| Table S6 | Loci associated with OUD conditional on AUD at condFDR<0.01 |
| Table S7 | Shared loci between AUD and CUD at conjFDR<0.05 |
| Table S8 | Shared loci between AUD and OUD at conjFDR<0.05 |
| Table S9 | GWAS tested for novelty |
| Table S10 | Previous associations with related substance use traits |

| **Table S1. Univariate MiXeR estimates for AUD, CUD and OUD** | | | | | | | | | | | | |
| --- | --- | --- | --- | --- | --- | --- | --- | --- | --- | --- | --- | --- |
| Univariate MiXeR results. h^2^_SNP_: SNP-based heritability estimate. Polygenicity_80, 90, 100_: The number of causal variants with strongest effects required to explain 80, 90 and 100% of SNP-based heritability. AIC and BIC are indices of model fit. Abbreviations: AIC, Akaike information criterion; BIC, Bayesian information criterion; AUD, alcohol use disorder; CUD, cannabis use disorder; OUD, opioid use disorder. | | | | | | | | | | | | |
|  | h2SNP | | \| 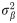Discoverability ( ) \| \| --- \| | | Polygenicity80 | | Polygenicity90 | | Polygenicity100 | |  |  |
| Phenotype | Mean | SD | Mean | SD | Mean | SD | Mean | SD | Mean | SD | AIC | BIC |
| AUD | 0.10 | 1.38E-03 | 1.65E-05 | 8.71E-07 | 6663.58 | 383.52 | 9362.91 | 538.88 | 29350.84 | 1689.27 | 128.93 | 119.42 |
| CUD | 0.01 | 2.90E-04 | 3.26E-06 | 2.01E-07 | 4312.09 | 298.50 | 6058.86 | 419.42 | 18993.30 | 1314.80 | 28.76 | 19.16 |
| OUD | 0.10 | 4.38E-03 | 3.18E-05 | 5.01E-06 | 3605.68 | 703.60 | 5066.29 | 988.62 | 15881.81 | 3099.14 | 9.25 | 0.39 |

| **Table S2. Model fit for bivariate AUD, CUD and OUD analysis with MiXeR** | | | | | |
| --- | --- | --- | --- | --- | --- |
| Results from Akaike information criterion (AIC) and Bayesian information criterion (BIC), calculated for the full versus reduced bivariate MiXeR model, constrained to minimal feasible polygenic overlap (“best vs min.”) or to the complete polygenic overlap (“best vs max.”). A positive AIC or BIC value provides evidence for the polygenic overlap. AUD, alcohol use disorder; CUD, cannabis use disorder; OUD, opioid use disorder. | | | | | |
|  |  | AIC | | BIC | |
| Trait 1 | Trait 2 | best vs min. overlap | best vs max. overlap | best vs min. overlap | best vs max. overlap |
| AUD | CUD | -2.00 | -1.99 | -11.50 | -11.50 |
| AUD | OUD | -1.83 | -1.73 | -10.69 | -10.58 |
| CUD | OUD | -0.59 | -0.87 | -9.44 | -9.72 |

| **Table S3. Genomic loci associated with alcohol use disorder (AUD) at condFDR<0.01 given association with cannabis use disorder (CUD)** | | | | | | | | | | | | | | | | | | | | |
| --- | --- | --- | --- | --- | --- | --- | --- | --- | --- | --- | --- | --- | --- | --- | --- | --- | --- | --- | --- | --- |
| The most strongly associated lead SNPs in independent genomic loci are shown after merging regions <250 KB apart into a single locus. Also shown are p-values and effect sizes (z-scores) from the original summary statistics. zAUD=z-value in AUD, zCUD=z-value in CUD, ce=concordant effect, pAUD=p value in AUD, pCUD=p value in CUD, ogs= overall gene score, nlAUD=novel loci in AUD, nlCUD=novel loci in CUD, lgc=loci in GWAS catalog, lgcrt=loci_in_GWAScatalog_related_trait, eb=expressed in brain, rs1146751 (A1=TA), rs11628909 (A1=GA), rs7596419 (A2=TCACACA), rs7610118 (A2=ATTAT), rs13107325 (A2=AT), rs61057384 (A2=TCAC), rs2718167 (A2=GATAA), rs1921035 (A2=GA), rs12919399 (A2=TA), rs973579 (PMID:32451486, lgcrt=alcohol consumption (drinks per week) (MTAG)), lts=low tissue specificity, te=tissue enhanced, ten=tissue enriched, ge=group enriched, m.=muscle, tis=tissue. Skel.=Skeletal, gl.=gland, Pit.=Pituitary, Epi.=Epididymis, Par.=Parathyroid, Int.=Intestine, Lym.=Lymphoid, Pla.=Placenta, Adr.=Adrenal, Eso.=Esophagus, T.=Tongue, R.=Retina, H.=Heart, BM=Bone marrow, Pr.=Prostate, B.=Brain, Br.=Breast, Ova.=Ovary. | | | | | | | | | | | | | | | | | | | | |
| **chr** | **lead SNP** | **leadbp** | **minbp** | **maxbp** | **fdr** | **A1** | **A2** | **zAUD** | **zCUD** | **cf** | **pAUD** | **pCUD** | **gene** | **ogs** | **nlAUD** | **nlCUD** | **lgc** | **lgcrt** | **tissue specificity** | **eb** |
| 1 | rs2022028 | 44606565 | 44589058 | 44830070 | 7.17E-05 | C | A | -6.39 | -1.12 | Yes | 1.71E-10 | 2.67E-01 | DMAP1 | 0.15 | No | No | No | No | lts | Yes |
| 1 | rs2310819 | 66440096 | 66334518 | 66547212 | 9.72E-08 | T | C | -6.74 | -5.98 | Yes | 1.54E-11 | 2.53E-09 | LEPROT | 0.07 | No | No | No | No | lts | Yes |
| 1 | rs34305371 | 72733610 | 72029085 | 72919499 | 5.17E-03 | T | C | 4.87 | 0.82 | Yes | 1.13E-06 | 4.10E-01 | NEGR1 | 0.18 | No | No | No | No | te (B.) | Yes |
| 1 | rs6681595 | 73827012 | 73766431 | 73991651 | 5.39E-05 | T | G | 5.89 | 5.04 | Yes | 3.95E-09 | 4.17E-07 | N/A | N/A | No | No | No | No | N/A | N/A |
| 1 | rs2172794 | 79997655 | 79844443 | 80091154 | 1.88E-03 | C | G | 4.74 | 3.18 | Yes | 2.10E-06 | 1.44E-03 | ADGRL4 | 0.03 | Yes | Yes | No | No | te (Adipose tis.) | Yes |
| 1 | rs372701525 | 80860383 | 80784642 | 80871734 | 6.28E-04 | C | T | -5.64 | -4.36 | Yes | 1.66E-08 | 1.28E-05 | N/A | N/A | Yes | Yes | No | No | N/A | N/A |
| 1 | rs1526480 | 91209986 | 91189731 | 91227215 | 1.72E-03 | T | C | -4.75 | -6.18 | Yes | 2.03E-06 | 5.91E-10 | BARHL2 | 0.06 | No | No | No | No | ten (B.) | Yes |
| 1 | rs4949953 | 97904780 | 97895762 | 97926839 | 1.91E-04 | C | T | 5.85 | 3.98 | Yes | 4.90E-09 | 6.60E-05 | DPYD | 0.07 | No | Yes | No | No | te (Liver) | Yes |
| 1 | rs1343546 | 165137227 | 165128148 | 165169159 | 5.19E-03 | G | A | 5.25 | 0.62 | Yes | 1.53E-07 | 5.35E-01 | LMX1A | 0.04 | No | Yes | No | No | ten (Choroid plexus) | Yes |
| 1 | rs12125739 | 197529577 | 197312162 | 197813032 | 5.84E-04 | C | G | 5.05 | 2.34 | Yes | 4.39E-07 | 1.93E-02 | LHX9 | 0.11 | Yes | Yes | No | No | ten (Ovary) | Yes |
| 1 | rs12138531 | 236901414 | 236881403 | 236905585 | 2.15E-03 | C | T | -5.07 | -2.57 | Yes | 3.90E-07 | 1.05E-02 | ACTN2 | 0.13 | Yes | Yes | No | No | ge (H. m., Skel. m., T.) | Yes |
| 2 | rs780093 | 27742603 | 27242761 | 28113911 | 5.16E-07 | T | C | -9.23 | 0.04 | No | 2.66E-20 | 9.69E-01 | FNDC4 | 0.30 | No | Yes | No | No | te (Adr. gl, Liver) | Yes |
| 2 | rs4953152 | 45170153 | 45121466 | 45175585 | 2.24E-06 | G | A | -8.49 | -2.99 | Yes | 2.02E-17 | 2.89E-03 | SIX3 | 0.09 | No | No | No | No | te (B., pit. gl, R.) | Yes |
| 2 | rs2312143 | 58155829 | 57942987 | 58505679 | 1.28E-05 | A | G | -6.52 | -3.31 | Yes | 7.01E-11 | 9.03E-04 | VRK2 | 0.09 | No | No | No | No | lts | Yes |
| 2 | rs146542417 | 61730787 | 61388164 | 61799575 | 7.23E-03 | G | C | 4.86 | 1.63 | Yes | 1.17E-06 | 1.06E-01 | SANBR | 0.32 | Yes | Yes | No | No | te (R.) | Yes |
| 2 | rs7574268 | 73904872 | 73548787 | 74051758 | 7.32E-04 | G | A | 5.88 | 2.67 | Yes | 4.10E-09 | 7.80E-03 | TPRKB | 0.33 | No | Yes | No | No | lts | Yes |
| 2 | rs7596419 | 101247441 | 101232647 | 101317319 | 2.60E-03 | T | *T* | -4.95 | -2.75 | Yes | 7.34E-07 | 6.07E-03 | PDCL3 | 0.10 | Yes | Yes | No | No | lts | Yes |
| 2 | rs13428598 | 144250487 | 144144663 | 144356509 | 3.14E-06 | C | T | 7.90 | 3.83 | Yes | 2.85E-15 | 1.33E-04 | ARHGAP15 | 0.14 | No | Yes | No | No | ge (BM, Lymph. tis.) | Yes |
| 2 | rs79228308 | 146648278 | 146521658 | 146655593 | 9.21E-03 | C | G | -4.67 | -1.23 | Yes | 3.05E-06 | 2.17E-01 | N/A | N/A | No | No | No | No | N/A | Yes |
| 2 | rs72859290 | 147981914 | 147886582 | 147981914 | 4.97E-04 | G | A | -5.97 | -2.52 | Yes | 2.32E-09 | 1.18E-02 | N/A | N/A | No | Yes | No | No | N/A | Yes |
| 2 | rs57761252 | 161865998 | 161865901 | 162095003 | 1.25E-03 | T | C | 5.61 | 2.47 | Yes | 2.03E-08 | 1.37E-02 | TANK | 0.15 | No | No | No | No | lts | Yes |
| 2 | rs2970931 | 162873188 | 162796517 | 162891848 | 2.71E-03 | A | T | 5.26 | 2.43 | Yes | 1.42E-07 | 1.50E-02 | DPP4 | 0.13 | No | No | No | No | Te (Int., Par. gl, Pla., Pr.) | Yes |
| 2 | rs75139511 | 175193863 | 175008676 | 175289259 | 5.82E-03 | G | A | 4.94 | 2.24 | Yes | 8.02E-07 | 2.55E-02 | CIR1 | N/A | Yes | Yes | No | No | lts | Yes |
| 2 | rs2251139 | 178068159 | 178011652 | 178146269 | 6.69E-03 | T | G | 4.36 | 3.81 | Yes | 1.31E-05 | 1.41E-04 | NFE2L2 | 0.30 | Yes | Yes | No | No | lts | Yes |
| 2 | rs2059924 | 185785947 | 185538930 | 185915220 | 1.62E-04 | G | A | 5.52 | 3.74 | Yes | 3.34E-08 | 1.80E-04 | ZNF804A | 0.06 | No | Yes | No | No | te (B., R.) | Yes |
| 2 | rs13034702 | 200719755 | 200705623 | 201042954 | 8.19E-03 | C | A | 4.82 | -1.31 | No | 1.43E-06 | 1.92E-01 | MAIP1 | 0.19 | Yes | Yes | No | No | lts | Yes |
| 2 | rs2713540 | 227140486 | 227020853 | 227181683 | 8.72E-03 | A | G | 5.27 | 0.33 | Yes | 1.35E-07 | 7.45E-01 | IRS1 | 0.07 | No | Yes | No | No | lts | Yes |
| 3 | rs7610118 | 16849374 | 16716571 | 16879840 | 5.89E-04 | A | *A* | 5.82 | 3.33 | Yes | 5.89E-09 | 9.18E-04 | PLCL2 | 0.19 | No | No | No | No | te (Skel. m.) | Yes |
| 3 | rs1865741 | 49362892 | 48412246 | 49890967 | 6.52E-08 | A | G | 6.83 | 6.72 | Yes | 8.49E-12 | 1.46E-11 | AMT | 0.29 | No | No | No | No | te (Liver) | Yes |
| 3 | rs9842128 | 56014117 | 55936417 | 56403636 | 1.50E-03 | G | A | -5.06 | -4.16 | Yes | 4.13E-07 | 3.30E-05 | ERC2 | 0.10 | No | No | No | No | ten (B.) | Yes |
| 3 | rs6804453 | 81873269 | 81675255 | 81985189 | 2.58E-03 | A | G | -5.46 | -4.07 | Yes | 4.71E-08 | 4.73E-05 | GBE1 | N/A | Yes | Yes | No | No | te (Skel. m.) | Yes |
| 3 | rs1146751 | 84889597 | 84841679 | 84951716 | 2.96E-05 | *T* | T | 6.21 | 5.04 | Yes | 5.37E-10 | 5.45E-07 | CADM2 | 0.12 | No | No | No | No | ge (B., R.) | Yes |
| 3 | rs11921010 | 85434260 | 85404030 | 85671909 | 1.48E-05 | T | G | 5.83 | 5.50 | Yes | 5.43E-09 | 4.13E-08 | CADM2 | 0.13 | No | No | No | No | ge (B., R.) | Yes |
| 3 | rs827162 | 158023087 | 157829953 | 158329139 | 6.59E-03 | C | T | -5.21 | -1.55 | Yes | 1.91E-07 | 1.21E-01 | RSRC1 | 0.28 | No | Yes | No | No | lts | Yes |
| 4 | rs7441967 | 30693576 | 30590113 | 30811774 | 1.54E-03 | T | C | -5.41 | -1.79 | Yes | 6.39E-08 | 7.30E-02 | PCDH7 | 0.07 | Yes | Yes | No | No | te (Blood vessel) | Yes |
| 4 | rs13149518 | 39406987 | 39153836 | 39425248 | 3.08E-06 | G | A | -9.14 | 1.49 | No | 6.18E-20 | 1.35E-01 | KLHL5 | 0.18 | No | Yes | No | No | lts | Yes |
| 4 | rs7438336 | 99729451 | 99656465 | 100155470 | 2.04E-07 | C | T | 7.30 | -1.67 | No | 2.77E-13 | 9.39E-02 | EIF4E | 0.05 | No | Yes | No | No | lts | Yes |
| 4 | rs13107325 | 103188709 | 102321252 | 103387161 | 2.67E-06 | A | *A* | 12.00 | 4.64 | Yes | 3.76E-33 | 3.36E-06 | SLC39A8 | 0.22 | No | No | No | No | te (Lung) | Yes |
| 4 | rs1460686 | 136211250 | 136047869 | 136244789 | 4.70E-03 | C | T | 4.93 | 2.51 | Yes | 8.13E-07 | 1.20E-02 | N/A | N/A | Yes | Yes | No | No | N/A | N/A |
| 4 | rs4546191 | 139847208 | 139831318 | 139875686 | 6.76E-06 | C | T | 6.26 | 2.03 | Yes | 3.74E-10 | 4.31E-02 | NOCT | 0.15 | No | Yes | No | No | te (BM) | Yes |
| 4 | rs13102826 | 143602404 | 143487451 | 144130103 | 2.16E-04 | T | G | -6.14 | -3.54 | Yes | 8.38E-10 | 4.07E-04 | INPP4B | 0.20 | No | Yes | No | No | lts | Yes |
| 5 | rs61057384 | 7394979 | 7371573 | 7440086 | 1.23E-03 | T | *T* | 5.45 | 2.47 | Yes | 5.10E-08 | 1.36E-02 | ADCY2 | 0.13 | Yes | Yes | No | No | ge (B., Skel. m., T.) | Yes |
| 5 | rs159544 | 60489247 | 60069057 | 60843706 | 1.24E-03 | T | C | 4.84 | 4.80 | Yes | 1.32E-06 | 1.77E-06 | ELOVL7 | 0.24 | No | No | No | No | lts | Yes |
| 5 | rs6449591 | 61535659 | 61398053 | 61808620 | 4.47E-04 | C | G | -6.01 | -2.28 | Yes | 1.81E-09 | 2.27E-02 | DIMT1 | 0.15 | No | No | No | No | lts | Yes |
| 5 | rs1599408 | 153296017 | 153266100 | 153356566 | 2.86E-03 | G | A | 5.35 | 1.68 | Yes | 8.98E-08 | 9.27E-02 | FAM114A2 | 0.23 | No | Yes | No | No | lts | Yes |
| 5 | rs17069646 | 167452251 | 167403035 | 167461166 | 3.88E-03 | A | G | -4.94 | -3.45 | Yes | 7.72E-07 | 5.45E-04 | WWC1 | 0.03 | No | No | No | No | te (Salivary gl) | Yes |
| 6 | rs12524640 | 11492676 | 11470114 | 11597638 | 1.99E-03 | A | G | -5.23 | -2.27 | Yes | 1.69E-07 | 2.27E-02 | TMEM170B | 0.14 | No | No | No | No | te (BM) | Yes |
| 6 | rs1322537 | 19023726 | 19023726 | 19191459 | 3.17E-03 | C | T | 4.58 | 3.19 | Yes | 4.60E-06 | 1.44E-03 | N/A | N/A | No | Yes | No | No | N/A | N/A |
| 6 | rs766406 | 26319588 | 26254271 | 26338697 | 9.01E-07 | G | A | 6.39 | 2.76 | Yes | 1.67E-10 | 5.74E-03 | H4C8 | 0.19 | No | No | No | No | te (Lymph. tis., Skel. m.) | Yes |
| 6 | rs57437999 | 41345126 | 41336101 | 41371275 | 9.12E-03 | A | G | 4.72 | 0.39 | Yes | 2.33E-06 | 6.94E-01 | NCR2 | 0.06 | Yes | Yes | No | No | Not detected | Yes |
| 6 | rs2784867 | 163840529 | 163794814 | 164006012 | 6.83E-04 | T | C | 5.49 | 1.43 | Yes | 4.04E-08 | 1.53E-01 | QKI | N/A | Yes | Yes | No | No | te (B., T.) | Yes |
| 7 | rs10253153 | 1001974 | 998802 | 1019536 | 1.82E-03 | A | G | 5.75 | 0.95 | Yes | 8.87E-09 | 3.41E-01 | ADAP1 | 0.30 | Yes | Yes | No | No | te (B.) | Yes |
| 7 | rs13235543 | 73013901 | 72854549 | 73058017 | 5.61E-04 | C | T | -5.04 | 2.35 | No | 4.72E-07 | 1.88E-02 | MLXIPL | 0.37 | No | Yes | No | No | ten (Liver) | Yes |
| 7 | rs17685 | 75616105 | 75607155 | 75813576 | 7.95E-04 | G | A | 5.28 | 3.82 | Yes | 1.28E-07 | 1.39E-04 | POR | 0.55 | No | Yes | No | No | te (Liver) | Yes |
| 7 | rs1155397 | 114953597 | 114940159 | 115098951 | 6.70E-06 | G | A | 6.58 | 3.23 | Yes | 4.64E-11 | 1.21E-03 | MDFIC | 0.01 | No | No | No | No | lts | Yes |
| 7 | rs10270624 | 119686018 | 119610438 | 119752599 | 7.19E-03 | A | G | 5.03 | 2.80 | Yes | 4.78E-07 | 5.30E-03 | KCND2 | 0.03 | Yes | Yes | No | No | ten (B.) | Yes |
| 7 | rs2718167 | 135093445 | 135050259 | 135221170 | 1.68E-07 | G | *G* | 7.45 | 1.26 | Yes | 9.12E-14 | 2.09E-01 | STMP1 | 0.14 | No | No | No | No | lts | Yes |
| 7 | rs2098112 | 153487944 | 153451274 | 153497853 | 3.25E-04 | C | A | -5.92 | -3.41 | Yes | 3.19E-09 | 6.24E-04 | DPP6 | 0.05 | No | Yes | No | No | te (B., Endometrium) | Yes |
| 8 | rs73169501 | 2127805 | 2104643 | 2214925 | 1.50E-03 | A | C | -4.79 | -3.71 | Yes | 1.70E-06 | 2.16E-04 | MYOM2 | 0.18 | No | Yes | No | No | ge (H. m., Skel. m.) | Yes |
| 8 | rs13276082 | 21823184 | 21770372 | 21872853 | 3.97E-04 | -1 | -1 | 5.86 | 2.95 | Yes | 4.56E-09 | 3.36E-03 | GFRA2 | 0.19 | No | Yes | No | No | te (Testis, Thyroid gl) | Yes |
| 8 | rs73229090 | 27442127 | 27412605 | 27453579 | 8.18E-03 | G | A | 4.29 | 6.63 | Yes | 1.75E-05 | 3.24E-11 | EPHX2 | 0.22 | Yes | No | No | No | te (Liver) | Yes |
| 8 | rs2576589 | 57425647 | 57221079 | 57437154 | 1.73E-04 | C | T | 6.09 | 2.59 | Yes | 1.14E-09 | 9.13E-03 | PENK | 0.30 | No | Yes | No | No | ge (Adr. gl, B.) | Yes |
| 8 | rs1822717 | 64956228 | 64700949 | 65073605 | 2.30E-05 | C | T | -6.63 | -1.49 | Yes | 3.32E-11 | 1.35E-01 | N/A | N/A | No | No | No | No | N/A | N/A |
| 8 | rs72671424 | 93056264 | 92976563 | 93180965 | 2.72E-03 | T | G | 4.92 | 2.39 | Yes | 8.66E-07 | 1.69E-02 | RUNX1T1 | 0.08 | No | No | No | No | te (B.) | Yes |
| 8 | rs6469450 | 114179599 | 114053798 | 114222232 | 8.31E-03 | C | G | -5.07 | -3.16 | Yes | 3.92E-07 | 1.55E-03 | CSMD3 | 0.17 | No | Yes | No | No | te (B.) | Yes |
| 9 | rs10986606 | 127933895 | 127780246 | 128149249 | 5.82E-05 | T | A | 6.02 | 6.28 | Yes | 1.73E-09 | 2.97E-10 | PPP6C | 0.37 | No | No | No | No | lts | Yes |
| 9 | rs456205 | 136911146 | 136876021 | 136925744 | 3.67E-03 | A | G | -5.50 | -2.22 | Yes | 3.72E-08 | 2.54E-02 | BRD3 | 0.22 | No | Yes | No | No | lts | Yes |
| 10 | rs1291854 | 11110195 | 11082192 | 11128578 | 1.60E-03 | G | T | -5.60 | -0.71 | Yes | 2.15E-08 | 4.78E-01 | CELF2 | 0.08 | No | Yes | No | No | te (BM) | Yes |
| 10 | rs10748819 | 104068496 | 103931931 | 104170204 | 7.54E-03 | T | G | 5.20 | 1.27 | Yes | 2.04E-07 | 2.03E-01 | GBF1 | 0.08 | Yes | No | No | No | lts | Yes |
| 10 | rs79068167 | 110554726 | 110461674 | 110758956 | 1.02E-06 | A | G | 7.27 | 3.29 | Yes | 3.52E-13 | 9.56E-04 | N/A | N/A | No | Yes | No | No | N/A | N/A |
| 11 | rs10835372 | 28643913 | 28591168 | 28709434 | 3.32E-03 | A | T | 5.12 | 5.54 | Yes | 2.99E-07 | 3.03E-08 | N/A | N/A | No | No | No | No | N/A | N/A |
| 11 | rs7127254 | 46308549 | 46304368 | 46345665 | 2.79E-03 | A | G | -5.41 | -1.81 | Yes | 6.28E-08 | 6.98E-02 | DGKZ | 0.18 | No | Yes | No | No | te (B.) | Yes |
| 11 | rs12419692 | 47624714 | 47372377 | 47946836 | 4.39E-05 | C | G | 6.09 | 0.28 | Yes | 1.15E-09 | 7.84E-01 | MTCH2 | 0.38 | No | Yes | No | No | lts | Yes |
| 11 | rs10792112 | 57565800 | 57404779 | 57756568 | 5.98E-08 | G | A | -6.91 | -4.72 | Yes | 4.80E-12 | 2.46E-06 | MED19 | 0.31 | No | No | No | No | lts | Yes |
| 11 | rs61886926 | 64133552 | 64004237 | 64212852 | 2.35E-03 | T | C | 5.16 | 4.32 | Yes | 2.44E-07 | 1.64E-05 | PPP1R14B | 0.32 | No | No | No | No | lts | Yes |
| 11 | rs10736470 | 113418371 | 113317745 | 113451229 | 4.84E-08 | G | C | -7.87 | -4.76 | Yes | 3.51E-15 | 2.17E-06 | DRD2 | 0.06 | No | No | No | No | ge (B., pit. gl) | Yes |
| 11 | rs529373 | 119793573 | 119793024 | 119816512 | 4.36E-03 | G | A | 5.23 | 1.50 | Yes | 1.74E-07 | 1.35E-01 | NECTIN1 | 0.04 | Yes | Yes | No | No | te (Eso., Skin) | Yes |
| 11 | rs7106615 | 121632466 | 121569680 | 121661507 | 5.88E-08 | C | T | -7.67 | -4.31 | Yes | 1.78E-14 | 1.46E-05 | BLID | N/A | No | No | No | No | Not detected | Yes |
| 12 | rs10783446 | 51881725 | 51799968 | 51919819 | 2.57E-04 | A | C | 6.04 | 1.63 | Yes | 1.56E-09 | 1.03E-01 | SLC4A8 | 0.35 | No | Yes | No | No | ge (B., pit. gl, R., Testis) | Yes |
| 12 | rs1921035 | 81602449 | 81376497 | 81673764 | 9.79E-05 | G | *G* | 5.99 | 2.88 | Yes | 2.11E-09 | 4.16E-03 | ACSS3 | 0.28 | No | Yes | No | No | te (Liver) | Yes |
| 12 | rs113501182 | 100147234 | 100147234 | 100147234 | 6.64E-03 | T | G | -5.30 | -2.07 | Yes | 1.19E-07 | 3.85E-02 | ANKS1B | 0.05 | Yes | Yes | No | No | ten (B.) | Yes |
| 12 | rs56159960 | 110994068 | 110272635 | 111274102 | 2.12E-03 | T | G | 5.23 | 2.12 | Yes | 1.69E-07 | 3.41E-02 | HVCN1 | 0.21 | No | Yes | No | No | ten (Choroid plexus) | Yes |
| 13 | rs7992016 | 55695261 | 55679486 | 55971068 | 2.03E-03 | T | C | 5.10 | 4.55 | Yes | 3.31E-07 | 5.48E-06 | N/A | N/A | Yes | No | No | No | N/A | N/A |
| 13 | rs12877345 | 59359099 | 59304838 | 59483583 | 9.69E-03 | A | T | -5.03 | -2.95 | Yes | 4.84E-07 | 3.14E-03 | N/A | N/A | No | Yes | No | No | N/A | N/A |
| 13 | rs59899558 | 89072227 | 89060548 | 89136916 | 1.38E-04 | G | A | -6.03 | -1.74 | Yes | 1.62E-09 | 8.06E-02 | N/A | N/A | No | Yes | No | No | N/A | N/A |
| 13 | rs9556559 | 96974352 | 96665151 | 97029792 | 9.09E-04 | G | A | -5.54 | -3.66 | Yes | 3.08E-08 | 2.29E-04 | HS6ST3 | 0.17 | No | Yes | No | No | te (Blood vessel, B.) | Yes |
| 13 | rs9557376 | 100729363 | 100648356 | 100787374 | 9.91E-03 | A | T | -4.76 | -1.99 | Yes | 1.93E-06 | 4.75E-02 | PCCA | 0.12 | Yes | Yes | No | No | te (Epid.) | Yes |
| 13 | rs4509879 | 107668152 | 107579465 | 107677487 | 9.58E-03 | A | G | 4.28 | 1.82 | Yes | 1.83E-05 | 6.80E-02 | ARGLU1 | 0.01 | Yes | Yes | No | No | lts | Yes |
| 13 | rs73591198 | 108213021 | 108213021 | 108232800 | 3.12E-03 | A | G | 5.60 | 2.32 | Yes | 2.20E-08 | 2.09E-02 | NALF1 | 0.05 | Yes | Yes | No | No | te (B., pit. gl) | Yes |
| 13 | rs9301483 | 111530373 | 111527756 | 111565706 | 5.63E-03 | C | T | 5.07 | 1.90 | Yes | 4.06E-07 | 5.72E-02 | ANKRD10 | 0.09 | Yes | Yes | No | No | lts | Yes |
| 14 | rs850254 | 57350427 | 57338977 | 57381487 | 3.05E-04 | T | C | 5.67 | 5.17 | Yes | 1.43E-08 | 2.58E-07 | OTX2 | 0.06 | No | Yes | No | No | ge (Choroid plexus, R.) | Yes |
| 14 | rs11628909 | 58819226 | 58664909 | 58856709 | 7.21E-05 | *G* | G | -6.23 | -3.23 | Yes | 4.74E-10 | 1.30E-03 | ACTR10 | 0.24 | No | Yes | No | No | lts | Yes |
| 14 | rs28479795 | 79943606 | 79833494 | 79945162 | 4.45E-03 | C | T | 5.36 | 1.18 | Yes | 8.32E-08 | 2.38E-01 | NRXN3 | 0.02 | Yes | Yes | No | No | te (B., R.) | Yes |
| 14 | rs1405238 | 99733954 | 99733954 | 99750520 | 4.17E-04 | C | T | -5.55 | -3.15 | Yes | 2.92E-08 | 1.70E-03 | BCL11B | 0.09 | No | Yes | No | No | ge (B., Lym. tis., Skin) | Yes |
| 14 | rs28533661 | 104305794 | 104188920 | 104327732 | 6.61E-03 | A | G | -5.28 | -1.09 | Yes | 1.29E-07 | 2.72E-01 | PPP1R13B | 0.36 | No | Yes | No | No | lts | Yes |
| 15 | rs73403005 | 47645174 | 47613403 | 47685504 | 1.47E-07 | C | T | -6.93 | -3.60 | Yes | 4.32E-12 | 3.22E-04 | SEMA6D | 0.12 | No | No | No | No | te (Int., Plac.) | Yes |
| 15 | rs7177599 | 83717246 | 83502787 | 83977166 | 1.64E-04 | A | G | 5.68 | 3.80 | Yes | 1.37E-08 | 1.42E-04 | BTBD1 | 0.35 | Yes | No | No | No | te (Skel. m., T.) | Yes |
| 16 | rs115690621 | 13587082 | 13580639 | 13630970 | 6.18E-03 | A | G | 4.84 | 2.14 | Yes | 1.28E-06 | 3.21E-02 | ERCC4 | N/A | No | Yes | No | No | te (Skel. m.) | Yes |
| 16 | rs11644459 | 30095236 | 29924422 | 30118345 | 1.08E-05 | A | T | 6.78 | 2.06 | Yes | 1.21E-11 | 3.98E-02 | INO80E | 0.37 | No | Yes | No | No | lts | Yes |
| 16 | rs57292959 | 53799279 | 53797908 | 53848561 | 7.89E-07 | C | G | 8.32 | 1.95 | Yes | 8.87E-17 | 5.27E-02 | FTO | 0.08 | No | Yes | No | No | lts | Yes |
| 16 | rs8045019 | 54639332 | 54612783 | 54673195 | 9.75E-04 | A | G | 5.07 | 3.42 | Yes | 3.97E-07 | 6.66E-04 | IRX3 | 0.03 | No | Yes | No | No | te (Skin) | Yes |
| 16 | rs12919399 | 65767853 | 65754739 | 65785122 | 8.57E-03 | T | *T* | -5.32 | -1.75 | Yes | 1.04E-07 | 8.12E-02 | N/A | N/A | Yes | Yes | No | No | N/A | N/A |
| 16 | rs10514405 | 77027686 | 76993424 | 77045916 | 3.76E-03 | T | C | 5.15 | 1.26 | Yes | 2.56E-07 | 2.06E-01 | MON1B | N/A | Yes | Yes | No | No | lts | Yes |
| 17 | rs854784 | 18040690 | 18040690 | 18128474 | 1.12E-04 | C | T | -6.33 | -3.07 | Yes | 2.42E-10 | 1.97E-03 | SHMT1 | 0.35 | No | Yes | No | No | te (Kidney, Liver) | Yes |
| 17 | rs2012581 | 29560800 | 29389026 | 29735829 | 3.45E-04 | G | A | 5.61 | 4.33 | Yes | 2.06E-08 | 1.49E-05 | RAB11FIP4 | 0.19 | No | Yes | No | No | te (B., Testis) | Yes |
| 17 | rs60908520 | 41854587 | 41840229 | 41947073 | 8.32E-03 | C | T | 4.54 | 2.02 | Yes | 5.62E-06 | 4.27E-02 | MPP3 | 0.31 | Yes | Yes | No | No | ge (B., H. m.) | Yes |
| 17 | rs28646281 | 43977846 | 43463493 | 44797919 | 7.11E-03 | C | A | 5.24 | -1.80 | No | 1.65E-07 | 7.10E-02 | MAPT | 0.42 | No | Yes | No | No | te (B., Skel. m.) | Yes |
| 18 | rs9953231 | 35150526 | 35125113 | 35185858 | 7.31E-03 | C | A | -4.91 | -3.13 | Yes | 9.26E-07 | 1.62E-03 | CELF4 | 0.07 | Yes | Yes | No | No | te (B., pit. gl) | Yes |
| 18 | rs12458015 | 53305735 | 53195249 | 53463661 | 2.06E-04 | A | G | -5.28 | -3.20 | Yes | 1.32E-07 | 1.34E-03 | TCF4 | 0.08 | No | No | No | No | lts | Yes |
| 19 | rs117623407 | 32204489 | 32200518 | 32208909 | 2.68E-04 | T | G | 6.08 | 4.32 | Yes | 1.19E-09 | 1.46E-05 | TSHZ3 | 0.02 | Yes | Yes | No | No | te (Ovary) | Yes |
| 19 | rs973579 | 49241976 | 49168942 | 49334991 | 5.25E-07 | A | G | -6.61 | -2.93 | Yes | 3.95E-11 | 3.57E-03 | RASIP1 | 0.29 | No | No | Yes | Yes | lts | Yes |
| 20 | rs1535375 | 31163048 | 31093514 | 31189411 | 3.73E-04 | A | G | -5.56 | -3.13 | Yes | 2.72E-08 | 1.66E-03 | NOL4L | 0.25 | No | No | No | No | te (R.) | Yes |
| 20 | rs6015611 | 37308301 | 37305421 | 37337985 | 2.38E-04 | C | A | 5.49 | 2.28 | Yes | 4.07E-08 | 2.28E-02 | ARHGAP40 | 0.09 | No | Yes | No | No | ge (Br., Epi., Eso., Skin) | Yes |
| 20 | rs7272308 | 48583726 | 48435408 | 48651061 | 1.49E-03 | T | A | -5.51 | -1.58 | Yes | 3.66E-08 | 1.16E-01 | SPATA2 | 0.14 | No | Yes | No | No | lts | Yes |
| 22 | rs73176689 | 41782633 | 41719469 | 42216326 | 3.46E-03 | T | C | 4.76 | -0.38 | No | 1.91E-06 | 6.99E-01 | CSDC2 | 0.36 | No | No | No | No | te (Adr. gl, H. m., Ova.) | Yes |

| **Table S4. Genomic loci associated with cannabis use disorder (CUD) at condFDR<0.01 given association with alcohol use disorder (AUD)** | | | | | | | | | | | | | | | | | | | | |
| --- | --- | --- | --- | --- | --- | --- | --- | --- | --- | --- | --- | --- | --- | --- | --- | --- | --- | --- | --- | --- |
| The most strongly associated lead SNPs in independent genomic loci are shown after merging regions <250 KB apart into a single locus. Also shown are p-values and effect sizes (z-scores) from the original summary statistics. zAUD=z-value in AUD, zCUD=z-value in CUD, ce=concordant effect, pAUD=p value in AUD, pCUD=p value in CUD, ogs= overall gene score, nlAUD=novel loci in AUD, nlCUD=novel loci in CUD, lgc=loci in GWAS catalog, lgcrt=loci_in_GWAScatalog_related_trait, eb=expressed in brain, rs1392816 (A1=AG), rs6452787 (A1=TA), lts=low tissue specificity, te=tissue enhanced, ten=tissue enriched, ge=group enriched, m.=muscle, tis=tissue, Skel.=Skeletal, gl.=gland, Pit.=Pituitary, Epi.=Epididymis, Par.=Parathyroid, Int. Intestine, Lym.=Lymphoid, Pla.=Placenta, Adr.=Adrenal, Eso.=Esophagus, T.=Tongue, R.=Retina, H.=Heart, BM=Bone marrow, Pr.=Prostate. B.=Brain, Br.=Breast, Ova.=Ovary. | | | | | | | | | | | | | | | | | | | | |
| **chr** | **lead SNP** | **leadbp** | **minbp** | **maxbp** | **fdr** | **A1** | **A2** | **zCUD** | **zAUD** | **cf** | **pCUD** | **pAUD** | **gene** | **ogs** | **nlCUD** | **nlAUD** | **lgc** | **lgcrt** | **tissue specificity** | **eb** |
| 1 | rs1392816 | 66481188 | 66370116 | 66485442 | 3.95E-05 | *A* | G | 3.60 | 4.87 | Yes | 3.15E-04 | 1.17E-06 | LEPROT | 0.08 | No | No | No | No | lte | Yes |
| 1 | rs12122743 | 91220791 | 91200757 | 91234126 | 2.35E-03 | T | G | -3.35 | -5.44 | Yes | 8.05E-04 | 5.86E-08 | BARHL2 | 0.06 | No | No | No | No | te (Brain) | Yes |
| 5 | rs159543 | 60486515 | 60135962 | 60550041 | 5.28E-04 | A | G | 4.83 | 4.80 | Yes | 1.33E-06 | 1.75E-06 | ELOVL7 | 0.24 | No | No | No | No | lte | Yes |
| 5 | rs6452787 | 87712831 | 87514778 | 87822672 | 1.71E-03 | T | *T* | -3.23 | 3.07 | No | 1.24E-03 | 2.13E-03 | TMEM161B | 0.05 | No | Yes | No | No | lte | Yes |
| 5 | rs12519058 | 163085586 | 163070267 | 163124522 | 5.20E-03 | G | A | -1.75 | -2.67 | Yes | 7.96E-02 | 7.99E-03 | MAT2B | 0.05 | Yes | Yes | No | No | lte | Yes |
| 6 | rs975303 | 19028788 | 19003340 | 19191459 | 1.70E-03 | C | T | 4.55 | 3.23 | Yes | 5.23E-06 | 1.17E-03 | N/A | N/A | Yes | No | No | No | N/A | N/A |
| 6 | rs6456732 | 26514445 | 26410800 | 26671135 | 5.33E-04 | C | T | -4.08 | -4.06 | Yes | 4.61E-05 | 5.40E-05 | HMGN4 | 0.19 | No | No | No | No | lte | Yes |
| 7 | rs13247874 | 73010442 | 72856430 | 73056750 | 7.78E-03 | C | T | -3.86 | 3.06 | No | 1.12E-04 | 2.29E-03 | MLXIPL | 0.33 | Yes | No | No | No | te (Liver) | Yes |
| 7 | rs7783012 | 114116881 | 114015707 | 114287116 | 6.43E-06 | C | A | -3.57 | -5.61 | Yes | 3.60E-04 | 1.68E-08 | FOXP2 | 0.03 | No | No | No | No | te (Intestine) | Yes |
| 8 | rs73169469 | 2104643 | 2073048 | 2214925 | 7.35E-04 | G | A | -4.50 | -3.31 | Yes | 6.78E-06 | 9.56E-04 | MYOM2 | 0.21 | Yes | No | No | No | ge (H. m., Skel. m.) | Yes |
| 8 | rs11783093 | 27425349 | 27405576 | 27467183 | 2.62E-06 | -1 | -1 | 3.51 | 7.44 | Yes | 4.44E-04 | 1.28E-13 | EPHX2 | 0.23 | No | Yes | No | No | te (Liver) | Yes |
| 10 | rs1637570 | 118619529 | 118607228 | 118778079 | 2.73E-03 | C | T | 3.88 | 5.45 | Yes | 1.06E-04 | 4.79E-08 | SHTN1 | 0.21 | No | Yes | No | No | te (Brain) | Yes |
| 11 | rs201282551 | 28256937 | 27828980 | 28543812 | 6.11E-03 | C | T | 2.98 | 3.22 | Yes | 2.88E-03 | 1.26E-03 | METTL15 | N/A | No | No | No | No | Lte | Yes |
| 11 | rs10896644 | 57563991 | 57409538 | 57731129 | 4.24E-03 | G | A | 4.81 | 3.27 | Yes | 1.52E-06 | 9.86E-04 | MED19 | 0.30 | No | No | No | No | Lte | Yes |
| 11 | rs11214687 | 113532067 | 113513936 | 113600513 | 3.18E-03 | A | C | 3.71 | 5.17 | Yes | 2.11E-04 | 2.34E-07 | TMPRSS5 | 0.27 | No | No | No | No | ge (Brain, Salivary gl.) | Yes |
| 15 | rs9806482 | 83503457 | 83502787 | 83514599 | 6.25E-04 | A | G | 4.56 | 4.74 | Yes | 5.02E-06 | 2.37E-06 | GOLGA6L10 | 0.22 | No | Yes | No | No | te (Testis) | Yes |
| 18 | rs12458015 | 53305735 | 53195249 | 53477784 | 2.83E-04 | A | G | -5.28 | -3.20 | Yes | 1.32E-07 | 1.34E-03 | TCF4 | 0.08 | No | No | No | No | lte | Yes |

| **Table S5. Genomic loci associated with alcohol use disorder (AUD) at condFDR<0.01 given association with opioid use disorder (OUD)** | | | | | | | | | | | | | | | | | | | | |
| --- | --- | --- | --- | --- | --- | --- | --- | --- | --- | --- | --- | --- | --- | --- | --- | --- | --- | --- | --- | --- |
| The most strongly associated lead SNPs in independent genomic loci are shown after merging regions <250 KB apart into a single locus. Also shown are p-values and effect sizes (z-scores) from the original summary statistics. zAUD=z-value in AUD, zOUD=z-value in OUD, ce=concordant effect, pAUD=p value in AUD, pOUD=p value in OUD, ogs=overall gene score. nlAUD=novel loci in AUD, nlOUD=novel loci in OUD, lgc=loci in GWAS catalog, lgcrt=loci_in_GWAScatalog_related_trait, eb=expressed in brain, rs13125440 (PMID:32451486.0, problematic alcohol use (MTAG)), rs7073987 (PMID: 32451486.0, AUD), rs11039216 (PMID: 33861876.0, AUD (consumption score)) lts=low tissue specificity, te=tissue enhanced, ten=tissue enriched, ge=group enriched, m.=muscle, tis=tissue. Skel.=Skeletal, gl.=gland. Pit.=Pituitary, Epi.=Epididymis, Par.=Parathyroid, Int. Intestine, Lym.=Lymphoid, Pla.=Placenta. Adr.=Adrenal, Eso.=Esophagus, T.=Tongue, R.=Retina, H.=Heart, BM=Bone marrow, Pr.=Prostate, B.=Brain, Br.=Breast, Ova.= Ovary. | | | | | | | | | | | | | | | | | | | | |
| **chr** | **lead SNP** | **leadbp** | **minbp** | **maxbp** | **fdr** | **A1** | **A2** | **zAUD** | **zOUD** | **cf** | **pAUD** | **pOUD** | **gene** | **ogs** | **nlAUD** | **nlOUD** | **lgc** | **lgcrt** | **tissue specificity** | **eb** |
| 4 | rs13125440 | 39422324 | 39413780 | 39425248 | 6.06E-03 | G | A | -8.20 | -0.72 | Yes | 2.40E-16 | 4.73E-01 | UGDH | 0.17 | No | Yes | Yes | Yes | te (Liver) | Yes |
| 4 | rs2213041 | 100247351 | 100246711 | 100291659 | 2.54E-06 | A | C | 8.40 | 1.49 | Yes | 4.46E-17 | 1.37E-01 | ADH1C | 0.20 | No | Yes | No | No | ge (Int., Liver, Stomach) | No |
| 5 | rs331757 | 124844266 | 124820663 | 124852240 | 1.33E-03 | G | A | 4.40 | 1.94 | Yes | 1.07E-05 | 5.28E-02 | N/A | N/A | No | Yes | No | No | N/A | N/A |
| 6 | rs7757112 | 154151020 | 153995311 | 154390607 | 3.12E-03 | C | T | -3.29 | -3.23 | Yes | 1.00E-03 | 1.25E-03 | OPRM1 | 0.04 | No | No | No | No | te (B., Testis) | Yes |
| 7 | rs2551777 | 135100476 | 135050259 | 135221170 | 6.48E-06 | C | T | 5.22 | 4.08 | Yes | 1.83E-07 | 4.60E-05 | STMP1 | 0.17 | No | No | No | No | lts | Yes |
| 8 | rs7460521 | 64971829 | 64882680 | 65069046 | 5.00E-03 | T | C | 4.84 | 3.49 | Yes | 1.33E-06 | 4.92E-04 | N/A | N/A | No | No | No | No | N/A | N/A |
| 8 | rs4735044 | 93057478 | 92976563 | 93180965 | 6.59E-03 | C | T | -3.43 | -3.77 | Yes | 6.10E-04 | 1.62E-04 | RUNX1T1 | 0.08 | No | No | No | No | te (B.) | Yes |
| 10 | rs7073987 | 110565868 | 110462847 | 110635222 | 6.73E-03 | T | C | -5.93 | -3.48 | Yes | 2.96E-09 | 5.11E-04 | N/A | N/A | No | No | Yes | Yes | N/A | N/A |
| 11 | rs11039216 | 47406592 | 47401448 | 47899030 | 3.92E-03 | C | T | -7.16 | -1.92 | Yes | 8.24E-13 | 5.47E-02 | SLC39A13 | 0.37 | No | Yes | Yes | Yes | lts | Yes |
| 15 | rs115583906 | 83534862 | 83523163 | 83977166 | 9.46E-03 | A | G | -4.53 | -2.75 | Yes | 5.85E-06 | 6.04E-03 | HOMER2 | 0.31 | Yes | Yes | No | No | te (Pancreas) | Yes |
| 16 | rs1547403 | 13593986 | 13580639 | 13630970 | 8.03E-05 | A | G | -4.11 | -2.34 | Yes | 3.93E-05 | 1.95E-02 | ERCC4 | 0.01 | No | Yes | No | No | te (Skel. m.) | Yes |
| 16 | rs3809627 | 30103160 | 30082508 | 30120442 | 3.30E-03 | A | C | 5.59 | 3.60 | Yes | 2.32E-08 | 3.24E-04 | INO80E | 0.31 | No | Yes | No | No | lts | Yes |
| 17 | rs74815784 | 42954517 | 42679912 | 42988177 | 7.58E-03 | C | T | 4.29 | 1.17 | Yes | 1.76E-05 | 2.43E-01 | EFTUD2 | 0.19 | Yes | Yes | No | No | lts | Yes |
| 19 | rs570794 | 49207651 | 49168942 | 49250239 | 5.84E-03 | C | T | 4.03 | 2.97 | Yes | 5.69E-05 | 3.00E-03 | MAMSTR | 0.27 | No | Yes | No | No | ge (Adr. gl., Skel. m., T.) | Yes |

| **Table S6. Genomic loci associated with opioid use disorder (OUD) at condFDR<0.01 given association with alcohol use disorder (AUD)** | | | | | | | | | | | | | | | | | | | | |
| --- | --- | --- | --- | --- | --- | --- | --- | --- | --- | --- | --- | --- | --- | --- | --- | --- | --- | --- | --- | --- |
| The most strongly associated lead SNPs in independent genomic loci are shown after merging regions <250 KB apart into a single locus. Also shown are p-values and effect sizes (z-scores) from the original summary statistics. zAUD=z-value in AUD, zOUD=z-value in OUD, ce=concordant effect, pAUD=p value in AUD, pOUD=p value in OUD, ogs=overall gene score, nlAUD=novel loci in AUD, nlOUD=novel loci in OUD, lgc=loci in GWAS catalog, lgcrt=loci_in_GWAScatalog_related_trait, eb=expressed in brain, lts=low tissue specificity, te=tissue enhanced, ten=tissue enriched, ge=group enriched, m.=muscle, tis=tissue, Skel.=Skeletal, gl.=gland, Pit.=Pituitary, Epi.=Epididymis, Par.=Parathyroid, Int.=Intestine, Lym.=Lymphoid, Pla.=Placenta, Adr.=Adrenal, Eso.=Esophagus, T.=Tongue, R.=Retina, H.=Heart, BM=Bone marrow, Pr.=Prostate, B.=Brain, Br.=Breast, Ova.= Ovary. | | | | | | | | | | | | | | | | | | | | |
| **chr** | **lead SNP** | **leadbp** | **minbp** | **maxbp** | **fdr** | **A1** | **A2** | **zAUD** | **zOUD** | **cf** | **pAUD** | **pOUD** | **gene** | **ogs** | **nlAUD** | **nlOUD** | **lgc** | **lgcrt** | **tissue specificity** | **eb** |
| 1 | rs12031155 | 53714139 | 53658317 | 53752134 | 1.00E-02 | T | C | -5.20 | -3.14 | Yes | 2.03E-07 | 1.68E-03 | CPT2 | 0.21 | No | Yes | No | No | te (Liver) | Yes |
| 2 | rs35942385 | 144208523 | 144145478 | 144263280 | 2.50E-03 | T | G | -4.51 | -5.58 | Yes | 6.61E-06 | 2.42E-08 | ARHGAP15 | 0.15 | No | No | No | No | ge (BM, Lym. tis.) | Yes |
| 6 | rs72828517 | 19036035 | 19023726 | 19191459 | 7.46E-03 | C | T | -4.35 | -2.63 | Yes | 1.36E-05 | 8.60E-03 | RNF144B | 0.00 | No | No | No | No | te (Skel. m.) | Yes |
| 6 | rs3094551 | 29354799 | 28674322 | 29354809 | 2.60E-04 | A | G | -5.26 | -2.40 | Yes | 1.42E-07 | 1.66E-02 | ZFP57 | 0.12 | No | Yes | No | No | ge (B., Heart m.) | Yes |
| 6 | rs1799971 | 154360797 | 154194081 | 154390607 | 2.07E-08 | G | A | -7.17 | -4.33 | Yes | 7.68E-13 | 1.50E-05 | OPRM1 | 0.21 | No | No | No | No | te (B., Testis) | Yes |
| 7 | rs3812281 | 135082751 | 135050259 | 135221170 | 7.41E-03 | C | T | 4.20 | 5.02 | Yes | 2.65E-05 | 5.17E-07 | STMP1 | 0.14 | No | No | No | No | lts | Yes |
| 8 | rs13262595 | 143316970 | 143309504 | 143340566 | 2.78E-04 | A | G | 5.15 | 3.48 | Yes | 2.65E-07 | 4.94E-04 | TSNARE1 | 0.20 | No | No | No | No | lts | Yes |
| 9 | rs405631 | 127968496 | 127780246 | 127987802 | 9.13E-03 | C | A | -5.14 | -4.80 | Yes | 2.73E-07 | 1.56E-06 | PPP6C | 0.35 | No | No | No | No | lts | Yes |
| 11 | rs1940701 | 112869404 | 112826867 | 112938783 | 5.62E-05 | C | T | 5.37 | 4.83 | Yes | 7.99E-08 | 1.37E-06 | NCAM1 | 0.16 | No | No | No | No | te (B., Heart m.) | Yes |
| 11 | rs11608185 | 113294976 | 113236199 | 113316102 | 6.80E-03 | T | C | 4.23 | 5.23 | Yes | 2.38E-05 | 1.73E-07 | TTC12 | 0.39 | No | No | No | No | te (Testis) | Yes |
| 15 | rs4702 | 91426560 | 91406146 | 91429042 | 1.19E-05 | G | A | 6.31 | 4.16 | Yes | 2.78E-10 | 3.13E-05 | FES | 0.38 | No | No | No | No | te (BM, Lym. tis.) | Yes |
| 16 | rs28567725 | 53826028 | 53797908 | 53845487 | 3.50E-04 | C | T | -4.98 | -7.98 | Yes | 6.32E-07 | 1.53E-15 | FTO | 0.09 | No | No | No | No | lts | Yes |
| 16 | rs61124172 | 61618296 | 61583172 | 61650572 | 1.74E-03 | G | A | 5.60 | 1.80 | Yes | 2.14E-08 | 7.24E-02 | CDH8 | 0.02 | No | Yes | No | No | ge (B., Seminal vesicle) | Yes |
| 20 | rs7272308 | 48583726 | 48435408 | 48604799 | 6.38E-03 | A | G | 5.14 | 0.77 | Yes | 2.74E-07 | 4.41E-01 | SPATA2 | 0.14 | No | No | No | No | lts | Yes |

| **Table S7. Genomic loci associated with alcohol use disorder (AUD) and cannabis use disorder (CUD) at conjFDR<0.05** | | | | | | | | | | | | | | | | | | | | | | |
| --- | --- | --- | --- | --- | --- | --- | --- | --- | --- | --- | --- | --- | --- | --- | --- | --- | --- | --- | --- | --- | --- | --- |
| The most strongly associated lead SNPs in independent genomic loci are shown after merging regions <250 KB apart into a single locus. Also shown are p-values and effect sizes (z-scores) from the original summary statistics. zAUD=z-value in AUD, zCUD=z-value in CUD, ce=concordant effect, pAUD=p value in AUD, pCUD=p value in CUD, ogs=overall gene score, nlAUD=novel loci in AUD, nlCUD=novel loci in CUD, lgc=loci in GWAS catalog, lgcrt=loci_in_GWAScatalog_related_trait, eb=expressed in brain, rs1624440 (A2=GA), rs2307018 (A2=AT) lts=low tissue specificity, te=tissue enhanced, ten=tissue enriched, ge=group enriched, m.=muscle, tis=tissue. Skel.=Skeletal, gl.=gland. Pit.=Pituitary, Epi.=Epididymis, Par.=Parathyroid, Int.=Intestine, Lym.=Lymphoid, Pla.=Placenta, Adr.=Adrenal, Eso.=Esophagus, T.=Tongue, R.=Retina, H.=Heart, BM=Bone marrow, Pr.=Prostate, B.=Brain, Br.=Breast, Ova.=Ovary, Kid.=Kidney. | | | | | | | | | | | | | | | | | | | | | | |
| **chr** | **lead SNP** | **leadbp** | **fdr** | **A1** | **A2** | **zCUD** | **zAUD** | **cf** | **pCUD** | **pAUD** | **gene** | **ogs** | **zCUDA** | **scCUDA** | **zAUDA** | **scAUDA** | **nlCUD** | **nlAUD** | **lgc** | **lgcrt** | **tissue specificity** | **eb** |
| 1 | rs7528604 | 66407352 | 4.17E-03 | T | C | 6.13 | 5.24 | Yes | 8.90E-10 | 1.67E-07 | LEPROT | 0.08 | 0.71 | Yes | N/A | N/A | No | No | No | No | lts | Yes |
| 1 | rs1526480 | 91209986 | 6.57E-03 | G | T | -4.75 | -6.18 | Yes | 2.03E-06 | 5.91E-10 | BARHL2 | 0.06 | -1.37 | Yes | -1.16 | Yes | No | No | No | No | tis. en. (B.) | Yes |
| 2 | rs112932070 | 27386676 | 4.00E-02 | T | C | 3.94 | 3.58 | Yes | 8.19E-05 | 3.49E-04 | KHK | 0.12 | 0.82 | Yes | 0.15 | Yes | Yes | No | No | No | ge (Int., Kid., Liver) | Yes |
| 2 | rs2588882 | 178087165 | 2.75E-02 | A | G | 4.30 | 4.19 | Yes | 1.68E-05 | 2.63E-05 | NFE2L2 | 0.31 | -0.24 | No | N/A | N/A | Yes | Yes | No | No | lts | Yes |
| 3 | rs59684465 | 49874246 | 2.35E-02 | C | G | -4.88 | -5.66 | Yes | 1.05E-06 | 1.49E-08 | RBM6 | 0.27 | -0.96 | Yes | N/A | N/A | No | No | No | No | lts | Yes |
| 3 | rs1463205 | 85596432 | 1.55E-02 | G | A | 5.15 | 5.66 | Yes | 2.55E-07 | 1.39E-08 | CADM2 | 0.13 | 0.56 | Yes | -0.15 | No | No | No | No | No | ge (B., Retina) | Yes |
| 5 | rs159544 | 60489247 | 1.24E-03 | T | C | 4.84 | 4.80 | Yes | 1.32E-06 | 1.77E-06 | ELOVL7 | 0.24 | 0.33 | Yes | 0.64 | Yes | No | No | No | No | lts | Yes |
| 6 | rs975303 | 19028788 | 3.49E-03 | C | T | 4.55 | 3.23 | Yes | 5.23E-06 | 1.17E-03 | N/A | N/A | 0.38 | Yes | 1.24 | Yes | Yes | No | No | No | N/A | N/A |
| 6 | rs1624440 | 26433329 | 3.66E-03 | G | *G* | 4.63 | 3.94 | Yes | 3.65E-06 | 8.07E-05 | HMGN4 | 0.39 | 0.33 | Yes | -0.01 | No | No | No | No | No | lts | Yes |
| 6 | rs2473569 | 40593860 | 2.84E-02 | C | T | -3.84 | -3.93 | Yes | 1.25E-04 | 8.96E-05 | LRFN2 | 0.14 | -0.20 | Yes | N/A | N/A | Yes | Yes | No | No | ge (B., Retina) | Yes |
| 7 | rs13225660 | 73006388 | 2.37E-02 | C | T | -3.91 | 2.99 | No | 9.19E-05 | 2.84E-03 | MLXIPL | 0.31 | 1.19 | Yes | -0.98 | Yes | Yes | No | No | No | tis. en. (Liver) | Yes |
| 7 | rs10249234 | 114129137 | 1.27E-02 | C | A | -4.15 | -5.28 | Yes | 3.36E-05 | 1.23E-07 | FOXP2 | 0.03 | N/A | N/A | N/A | N/A | No | No | No | No | te (Int.) | Yes |
| 8 | rs73169501 | 2127805 | 3.18E-03 | G | A | -4.79 | -3.71 | Yes | 1.70E-06 | 2.16E-04 | MYOM2 | 0.18 | 1.30 | No | 1.39 | No | Yes | No | No | No | ge (H. m., Skel. m.) | Yes |
| 8 | rs73229090 | 27442127 | 8.18E-03 | G | A | 4.29 | 6.63 | Yes | 1.75E-05 | 3.24E-11 | EPHX2 | 0.22 | 0.18 | Yes | 1.94 | Yes | No | Yes | No | No | te (Liver) | Yes |
| 9 | rs7867205 | 81436959 | 3.02E-02 | G | A | 4.00 | 3.94 | Yes | 6.33E-05 | 8.06E-05 | N/A | N/A | 1.78 | Yes | -0.44 | No | Yes | Yes | No | No | N/A | N/A |
| 10 | rs1637570 | 118619529 | 2.58E-02 | C | T | 3.88 | 5.45 | Yes | 1.06E-04 | 4.79E-08 | SHTN1 | 0.21 | 0.41 | Yes | -0.55 | No | No | Yes | No | No | tis. en. (B.) | Yes |
| 11 | rs1519480 | 27675712 | 2.83E-02 | A | G | 3.84 | 2.98 | Yes | 1.21E-04 | 3.00E-03 | LIN7C | 0.13 | N/A | N/A | 0.18 | Yes | No | No | No | No | lts | Yes |
| 11 | rs10896644 | 57563991 | 4.24E-03 | G | A | 4.81 | 3.27 | Yes | 1.52E-06 | 9.86E-04 | MED19 | 0.30 | -0.38 | No | 1.10 | Yes | No | No | No | No | lts | Yes |
| 11 | rs11214677 | 113477081 | 2.32E-02 | G | A | 4.28 | 5.05 | Yes | 1.88E-05 | 4.16E-07 | TMPRSS5 | 0.31 | 1.33 | Yes | 0.24 | Yes | No | No | No | No | ge (B., Sal. gl.) | Yes |
| 13 | rs9507729 | 27098237 | 4.93E-02 | A | C | -3.73 | -4.24 | Yes | 1.92E-04 | 2.09E-05 | WASF3 | 0.18 | -0.35 | Yes | 0.07 | No | Yes | Yes | No | No | te (B., Retina) | Yes |
| 15 | rs9806482 | 83503457 | 3.38E-03 | A | G | 4.56 | 4.74 | Yes | 5.02E-06 | 2.37E-06 | GOLGA6L10 | 0.22 | -0.11 | No | N/A | N/A | No | Yes | No | No | te (Testis) | Yes |
| 18 | rs12458015 | 53305735 | 2.83E-04 | A | G | -5.28 | -3.20 | Yes | 1.32E-07 | 1.34E-03 | TCF4 | 0.08 | -0.21 | Yes | -1.66 | Yes | No | No | No | No | lts | Yes |
| 19 | rs2307018 | 49244219 | 2.63E-02 | A | *A* | 5.47 | 3.88 | Yes | 4.37E-08 | 1.09E-04 | IZUMO1 | 0.35 | 1.12 | Yes | 1.19 | Yes | No | No | No | No | tis. en. (Testis) | Yes |

| **Table S8. Genomic loci associated with alcohol use disorder (AUD) and opioid use disorder (OUD) at conjFDR<0.05** | | | | | | | | | | | | | | | | | | | | | | |
| --- | --- | --- | --- | --- | --- | --- | --- | --- | --- | --- | --- | --- | --- | --- | --- | --- | --- | --- | --- | --- | --- | --- |
| The most strongly associated lead SNPs in independent genomic loci are shown after merging regions <250 KB apart into a single locus. Also shown are p-values and effect sizes (z-scores) from the original summary statistics. zAUD=z-value in AUD, zOUD=z-value in value in OUD, ce=concordant effect, pAUD=p value in AUD, pOUD=p value in OUD, ogs=overall gene score, nlAUD=novel loci in AUD, nlOUD=novel loci in OUD, lgc=loci in GWAS catalog, lgcrt=loci_in_GWAScatalog_related_trait, eb=expressed in brain, lts=low tissue specificity, te=tissue enhanced, ten=tissue enriched, ge=group enriched, m.=muscle, tis=tissue. Skel.=Skeletal, gl.=gland, Pit.=Pituitary, Epi.=Epididymis, Par.=Parathyroid, Int.=Intestine, Lym.=Lymphoid, Pla.=Placenta, Adr.=Adrenal, Eso.=Esophagus, T.=Tongue, R.=Retina, H.=Heart, BM=Bone marrow, Pr.=Prostate, B.=Brain, Br.=Breast, Ova.=Ovary, Kid.=Kidney. | | | | | | | | | | | | | | | | | | | | | | |
| **chr** | **lead SNP** | **leadbp** | **fdr** | **A1** | **A2** | **zCUD** | **zAUD** | **cf** | **pCUD** | **pAUD** | **gene** | **ogs** | **zCUDA** | **scCUDA** | **zAUDA** | **scAUDA** | **nlCUD** | **nlAUD** | **lgc** | **lgcrt** | **tissue specificity** | **eb** |
| 2 | rs13428598 | 144250487 | 1.04E-02 | T | C | -3.16 | -4.33 | Yes | 1.60E-03 | 1.49E-05 | ARHGAP15 | 0.14 | 2.84 | No | -59.66 | Yes | No | No | No | No | ge (BM, Lym. tis.) | Yes |
| 6 | rs9350100 | 19076417 | 3.38E-02 | C | T | -4.91 | -4.07 | Yes | 8.98E-07 | 4.74E-05 | RNF144B | 0.01 | N/A | N/A | N/A | N/A | No | No | No | No | te (Skel. m.) | Yes |
| 6 | rs1799971 | 154360797 | 6.02E-03 | G | A | -4.33 | -7.17 | Yes | 1.50E-05 | 7.68E-13 | OPRM1 | 0.21 | 0.88 | No | -0.25 | Yes | No | No | No | No | te (B., Testis) | Yes |
| 7 | rs3812281 | 135082751 | 7.41E-03 | C | T | 5.02 | 4.20 | Yes | 5.17E-07 | 2.65E-05 | STMP1 | 0.14 | -1.02 | No | 116.71 | Yes | No | No | No | No | lts | Yes |
| 8 | rs9297901 | 93036795 | 2.12E-02 | C | T | -3.45 | -3.87 | Yes | 5.53E-04 | 1.09E-04 | RUNX1T1 | 0.07 | 2.41 | No | -71.94 | Yes | No | No | No | No | te (B.) | Yes |
| 10 | rs2685484 | 110604592 | 3.36E-02 | C | T | -5.83 | -3.71 | Yes | 5.47E-09 | 2.06E-04 | N/A | N/A | N/A | N/A | N/A | N/A | No | No | No | No | N/A | N/A |
| 11 | rs4466874 | 112861434 | 2.37E-02 | C | T | 4.82 | 5.24 | Yes | 1.48E-06 | 1.57E-07 | NCAM1 | 0.16 | -2.13 | No | 103.49 | Yes | No | No | No | No | te (B., H. m.) | Yes |
| 11 | rs11608185 | 113294976 | 4.71E-02 | T | C | 5.23 | 4.23 | Yes | 1.73E-07 | 2.38E-05 | TTC12 | 0.39 | 3.55 | Yes | -54.73 | No | No | No | No | No | te (Testis) | Yes |
| 16 | rs200527 | 24759963 | 3.73E-02 | C | T | 3.19 | 3.77 | Yes | 1.41E-03 | 1.65E-04 | SLC5A11 | 0.15 | 0.23 | Yes | -6.02 | No | No | No | No | No | tis. en. (B.) | Yes |
| 16 | rs73530179 | 30107781 | 3.98E-02 | G | A | -5.93 | -3.76 | Yes | 3.12E-09 | 1.69E-04 | INO80E | 0.37 | N/A | N/A | N/A | N/A | No | Yes | No | No | lts | Yes |
| 16 | rs9922708 | 53831146 | 3.09E-02 | T | C | -7.86 | -4.43 | Yes | 3.76E-15 | 9.40E-06 | FTO | 0.09 | 0.71 | No | -51.08 | Yes | No | No | No | No | lts | Yes |

| **Table S9.** **GWAS tested for novelty** | | |
| --- | --- | --- |
| AUD=alcohol use disorder, CUD=cannabis use disorder, OUD=opioid use disorder. | | |
| **Trait** | **Study** | **PMID** |
| AUD | Kranzler et al 2019 | 37156939 |
|  | Wistrøm et al 2022 | 34472679 |
|  | Xu et al 2023 | 30940813 |
|  | Zhou et al 2023 | 38062264 |
|  | Icick et al 2024 | 40322774 |
|  | Davis et al 2025 | 40336358 |
|  |  |  |
| CUD | Johnson et al 2020 | 33096046 |
|  | Cheng et al 2023 | 37208114 |
|  | Xu et al 2023 | 30940813 |
|  | Levey et 2023 | 37985822 |
|  | Davis et al 2025 | 40336358 |
|  |  |  |
| OUD | Zhou et al 2020 | 32492095 |
|  | Deak 2022 | 35879402 |
|  | Kember 2022 | 36171425 |
|  | Xu et al 2023 | 30940813 |
|  | Holen et al 2023 | 37252880 |
|  | Holen et al 2024 | 38244365 |
|  | Davis et al 2025 | 40336358 |

| **Table S10. Previous associations with related substance use traits** | |
| --- | --- |
| **Term** |  |
| Addictive | |
| Addiction | |
| Alcohol | |
| Cannabis | |
| Heroin | |
| Lifetime cannabis use | |
| Nicotine | |
| Nicotine dependence | |
| Opioids | |
| Problematic alcohol use | |
| Problematic opioid prescription use | |
| Risky behaviour | |
| Smoking | |
| Substance | |
| Tobacco | |
